# The ratio of Zn to Cd supply as a determinant of metal-homeostasis gene expression in tobacco and its modulation by overexpressing the metal exporter AtHMA4

**DOI:** 10.1093/jxb/erw389

**Published:** 2016-10-17

**Authors:** Anna Barabasz, Maria Klimecka, Maria Kendziorek, Aleksandra Weremczuk, Anna Ruszczyńska, Ewa Bulska, Danuta Maria Antosiewicz

**Affiliations:** ^1^University of Warsaw, Faculty of Biology, Institute of Experimental Plant Biology and Biotechnology, Department of Plant Anatomy and Cytology, Miecznikowa str 1, 02-096 Warszawa, Poland; ^2^University of Warsaw, Faculty of Chemistry, Pasteura str. 1, 02-093 Warszawa, Poland

**Keywords:** AtHMA4, cadmium, SSH, tobacco, transformation, zinc.

## Abstract

This study links changes in the tobacco endogenous metal-homeostasis network caused by transgene expression with engineering of novel features. It also provides insight into the concentration-dependent mutual interactions between Zn and Cd, leading to differences in the metal partitioning between wild-type and transgenic plants. In tobacco, expression of the export protein AtHMA4 modified Zn/Cd root/shoot distribution, but the pattern depended on their concentrations in the medium. To address this phenomenon, the expression of genes identified by suppression subtractive hybridization and the Zn/Cd accumulation pattern were examined upon exposure to six variants of low/high Zn and Cd concentrations. Five tobacco metal-homeostasis genes were identified: *NtZIP2*, *NtZIP4*, *NtIRT1*-like, *NtNAS*, and *NtVTL.* In the wild type, their expression depended on combinations of low/high Zn and Cd concentrations; co-ordinated responses of *NtZIP1*, *NtZIP2*, and *NtVTL* were shown in medium containing 4 µM Cd, and at 0.5 µM versus 10 µM Zn. In transgenics, qualitative changes detected for *NtZIP1*, *NtZIP4*, *NtIRT1*-like, and *NtVTL* are considered crucial for modification of Zn/Cd supply-dependent Zn/Cd root/shoot distribution. Notwithstanding, *NtVTL* was the most responsive gene in wild-type and transgenic plants under all concentrations of Zn and Cd tested; thus it is a candidate gene for the regulation of metal cross-homeostasis processes involved in engineering new metal-related traits.

## Highlight

Modifications of endogenous metal-homeostasis traits, in particular co-ordinated regulation *of NtZIP1*, *NtZIP4*, *NtIRT1*-like, and *NtVTL*, contribute to the generation of the phenotype of *AtHMA4*-expressing tobacco.

## Introduction

As a micronutrient, zinc (Zn) is essential for plant growth and development; however, its excess could be harmful. The toxic metal cadmium (Cd) is non-essential for plants, but it is taken up by transporters for Zn, and also for other nutrient elements such as Fe or Ca. Once in the food chain, Cd causes health problems for consumers. On the other hand, plants with the ability to accumulate high amounts of Cd or Zn in their shoots are useful in phytoremediation of metal-contaminated soil (Palmgren *et al.*, 2008). Therefore, numerous attempts to engineer plants with altered metal root/shoot partitioning for phytoremediation or biofortification purposes have focused on modification of processes controlling root to shoot translocation of metals (Palmgren *et al.*, 2008; White and Broadley, 2009).

To alter a defined metal-related trait, a gene controlling the chosen characteristic is cloned from a plant and transformed into a selected species. Thus to increase the transfer of Zn and Cd from the roots to the shoots, AtHMA4 (a P_1B_-ATPase from *Arabidopsis thaliana*), which controls Zn/Cd root to shoot translocation by pumping metals into xylem vessels (Mills *et al.*, 2003, 2005; Verret *et al.*, 2004; Hanikenne *et al.*, 2008), was ectopically expressed in tobacco under the constitutive *Cauliflower mosaic virus* (CaMV) 35S promoter. However, the resulting transformants displayed certain features unrelated to the physiological function that this gene performs in *A. thaliana*.

First, contrary to expectations, Cd accumulation in the shoots was reduced (Siemianowski *et al.*, 2011). Microarray and biochemical analysis demonstrated that ectopic expression of *AtHMA4* in tobacco induced lignification of the external cell layer in the roots, which as a physical barrier was suggested to contribute to reduction of Cd accumulation. Expression analysis performed on whole roots did not show down-regulation of genes known to be involved in Cd uptake (Siemianowski *et al.*, 2014). It is generally accepted that young apical root segments (differentiation/root hair zone) play a pivotal role in the uptake of metals and are involved in xylem loading (Puig and Peñarrubia, 2009). Thus, genes differentially expressed in the apical root segments might not have been identified by expression analysis performed on mRNA isolated from the whole roots (Siemianowski *et al.*, 2014). Secondly, expression of *AtHMA4* modified Zn translocation to the shoots but the pattern depended on the Zn concentration in the medium. Recently, it has become evident that one of the major reasons for the appearance of unanticipated features of transgenic plants is modification of the host plant endogenous metal cross-homeostasis network due to transgene expression (Antosiewicz *et al.*, 2014). These processes are very poorly understood.

In this study, the goal was to better understand the mechanisms underlying generation of the metal-related phenotype of tobacco expressing 35S::*AtHMA4.* For that purpose, endogenous metal-homeostasis pathways specifically induced by expression of *AtHMA4* in the apical root segments were determined. Only some metal-homeostasis genes have been cloned and characterized in tobacco; therefore, to identify those differentially expressed in the root tips of transgenic plants [relative to the wild type (WT)] upon long exposure to Cd, analysis based on suppression subtractive hybridization (SSH) was performed. To address in transgenic plants the phenomenon of metal supply-dependent modifications of metal(s) distribution, *AtHMA4*-expressing and WT plants exposed to six combinations of Zn and Cd concentrations in the medium were compared for their Zn, Cd, and Fe accumulation/distribution ability, and for expression of tobacco metal homeostasis genes identified by SSH.

## Materials and methods

### Plant materials and growth conditions

Tobacco (*Nicotiana tabacum* cv. Xanthi) WT and *AtHMA4*-expressing plants (homozygous line 5 and line 9) generated previously (Siemianowski *et al.*, 2011) were used for the study.

Quarter-strength Knop’s medium was the control medium for experiments (Barabasz *et al.*, 2010). Surface-sterilized seeds [8% (v/v) sodium hypochloride, 2 min] were germinated on Petri dishes containing control medium supplemented with 2% (w/v) sucrose solidified with 1% (w/v) agar. Following 3 weeks of growth, the plantlets were transferred to 2.4 litre pots (six plants per pot) containing aerated liquid reference medium and kept for 1 week to allow them to adjust to hydroponic conditions (the medium was changed twice during this period). Depending on the experiment, 4-week-old seedlings (3 weeks on plates and 1 week on hydroponics) were further exposed for 11 d to a range of Zn and Cd (as CdCl_2_) concentrations added to the control medium (details are given in sections describing a given experiment). The medium was renewed every second day. The growth conditions applied were described by Kendziorek *et al.* (2014).

### Suppression subtractive hybridization (SSH)

#### Growth of plants

Four-week-old WT and *AtHMA4*-expressing tobacco (line 5) were further grown for 11 d under control conditions and in the presence of 0.25 µM Cd. At the end of the experiment, roots were collected for analysis. From each plant, half of the roots were dried and used to determine the Cd, Zn, and Fe concentration. From the other half, the apical root fragments (2.5 cm) were cut out, frozen in the liquid nitrogen, and used later for SSH analysis. The material was collected from three independently running replicate experiments. In each of them, root samples were collected from a total of 52 plants for each line.

#### RNA isolation

Total RNA was extracted using Trizol (TRI Reagent, Sigma) according to the manufacturer’s protocol followed by treatment with an RNase-Free DNase Set (Qiagen), and purification by a Syngen RNA Clean-up Kit (Syngen). RNA quality and quantity were determined by gel electrophoresis and spectrophotometric measurement using a NanoDrop 2000 (Nanodrop, Wilmington, DE, USA).

#### SSH procedures

To identify genes with differential expression upon exposure to Cd (both up- and down-regulated) in transgenic plants relative to the WT, two SSH cDNA libraries were constructed. For SSH, double-stranded cDNA was obtained by using the SMART-PCR cDNA synthesis Kit (Clontech) and Phusion Hot-Start II High-Fidelity DNA Polymerase with GC buffer (Thermo Scientific) from 800 ng of total RNA isolated from Cd-treated root samples from transgenic and WT tobacco. For the forward-subtracted cDNA library (T library), amplified cDNA from transgenic tobacco was used as the ‘tester’, whereas amplified cDNA from the WT was used as the ‘driver’. The reverse-subtracted cDNA library (WT library) was made with WT cDNA as the ‘tester’ and transgenic cDNA as the ‘driver’. PCR-selected cDNA subtraction was performed according to the instructions of the PCR-Select™ cDNA subtraction Kit (Clontech).

Following SSH, PCR products from subtracted samples were inserted into pGEM-T easy vector (Promega, USA) and then transferred into chemically competent *Escherichia coli* cells (JM109, >10^8^ cfu µl^–1^, Promega) to generate libraries. For the T library, 1704 colonies, and for the WT library, 1824 colonies were stored.

Obtained recombinant clones were used for PCR-based amplification of inserts. The differential screening of subtracted cDNA libraries was performed by reverse northern blot analysis to identify genes differentially expressed in transgenic tobacco (as compared with the WT) upon exposure to Cd. Each subtracted library was hybridized with forward- and reverse-subtracted probes according to the guidelines in the ‘PCR-Selected Differential Screening Kit’ (Clontech). The probes were digoxigenin labelled by using the ‘PCR DIG Probe Synthesis Kit’ (Roche). The results from the two hybridizations (two membranes containing the same clones hybridized with forward- and reverse-subtracted probes) were compared for each clone. Clones showing differential expression were selected for sequencing then subjected to bioinformatics analysis. The SSH procedure complemented with all details is given in Supplementary Protocol S1 at *JXB* online)

### DNA sequencing and sequence analysis

The positive clones obtained through reverse northern blot were sequenced by outsourced services (Genomed, Poland) from the SP6 promoter primer. Raw cDNA sequences were trimmed to eliminate vectors, adaptor sequences, and low quality regions. The insert sequences were manually assessed for similarities against the non-redundant (nr) public database at NCBI (http://blast.ncbi.nlm.nih.gov/Blast.cgi), using the BLASTN algorithm. The search was repeated when annotated sequences from *Nicotiana tomentosiformis* and *Nicotiana sylvestris* were made available. Obtained EST sequences were also re-entered using the BLASTX algorithm according to *A. thaliana* sequences. Because many obtained EST sequences contain a 5'- or 3'-untranslated region (UTR) and best hits from *A. thaliana* were not found, whole ‘best hits’ from *Nicotiana* species were also re-entered according to *A. thaliana* sequences using the BLASTX algorithm with threshold e-10.

### Real-time PCR-based gene expression analysis

RNA was isolated from root apical fragments stored at –80 °C using a Syngen Plant RNA Mini Kit (Syngen, Poland). RNA isolation, cDNA synthesis, and expression analysis were performed according to Kendziorek *et al.* (2014) with minor modifications. The reaction was carried out in a volume of 15 µl, containing 0.2 µM primers and 6 µl of 80× diluted reaction mixture obtained after cDNA synthesis. For real-time PCR, Luminaris HiGreen qPCR Master Mix (Thermo Scientific) was used. The tobacco PP2A gene was used as a reference/internal control and was amplified in parallel with the target gene allowing normalization of gene expression and providing quantification. All primers used in reactions are listed in Supplementary Table S1.

### Experiments using a different Zn, Fe, and Cd supply

#### Exposure to Zn and Fe deficiency conditions

Four-week-old WT plants, grown as described in the section ‘Plant materials and growth conditions’, were further cultivated for 11 d in a control medium lacking Zn or Fe (Zn or Fe were not added) and in parallel in a reference medium. At the end of the experiment, apical root fragments (2.5 cm) were cut off, frozen in liquid nitrogen, and stored at –80 °C until expression analysis. The experiment was done in triplicate with 14 plants for one biological replicate.

#### Exposure to a range of Cd and Zn concentrations

Four-week-old WT and transgenic plants (line 5 and line 9), grown as described in the section ‘Plant materials and growth conditions’, were exposed for 11 d to: (i) 0.25 µM and 4 µM Cd added to the liquid control medium (containing 0.5 µM Zn) or to the control medium supplemented with 10 µM Zn; or (ii) to control medium without Cd containing either 0.5 µM or 10 µM Zn. At the end of the experiment, roots were collected for the following analyses. (i) Expression analysis: the apical root fragments (2.5 cm) were cut off, frozen in liquid nitrogen, and stored at –80 °C until mRNA isolation. (ii) Determination of Zn, Cd, and Fe concentrations and their root/shoot distribution: roots were washed according to Barabasz *et al.* (2012); apical root fragments (2.5 cm) as well as the whole roots were collected and dried until analysis, (iii) Determination of Zn and Cd concentration in the apoplastic wash fluid (AWF): whole roots were washed as above, and immediately subjected to the extraction procedure (see below). Each experiment was done in triplicate with up to 10 plants for one biological replicate.

### Determination of Zn, Cd, and Fe concentrations

Elemental concentrations were determined according to Wojas *et al.* (2009). Measurements were done by atomic absorption spectrophotometry (TJA Solutions Solar M, Thermo Electron Manufacturer Ltd, Cambridge, UK).

### Apoplastic wash fluid analysis

The AWF was isolated from the whole roots of 5-week-old transgenic line 9 and WT plants grown as described in the section ‘Exposure to a range of Cd and Zn concentrations’. The methodology described by Yu *et al.* (1999) and Li *et al.* (2008) with minor modifications was applied. The whole roots were divided into 5 cm long segments. They were arranged in a 10 ml pipette tip with the cut ends facing down placed in a 1.5 ml Eppendorf tube, then in a 50 ml Falcon tube. The whole ensemble was first centrifuged at 500 *g* at 4 °C for 5 min. Afterwards, the pipette tips containing root segments were transferred to a new 1.5 ml Eppendorf tube, placed in a 50 ml Falcon tube again, and the AWF was collected after centrifugation at 3000 *g* at 4 °C for 20 min. It was stored at −20 °C. AWF isolation was repeated three times using at least three plants from each line per treatment. Cytoplasmic contamination was assessed by determination of K concentrations (Cosio *et al.*, 2005). The AWF was mixed 1:1 with 69% HNO_3_ and kept overnight at room temperature, then at 55 °C for 30 min. The concentration of Zn, Cd, and K was examined according to Barabasz *et al.* (2012).

### Statistical analysis

All data shown are from one experiment representative of a total of 3–5 independent experiments. For one biologically independent experiment (from an independent growth cycle), the ‘*n*’ value indicates the mean calculated from 3–10 samples. Statistical significance was determined by Student’s *t*-test and accepted at *P*≤0.05. Analysis was performed using one-way ANOVA followed by Tukey’s test using the R program.

## Results

### Suppression subtractive hybridization

In previous studies, it was shown that expression of *AtHMA4* in tobacco decreased the Cd concentration in roots and shoots (Siemianowski *et al.*, 2011). To learn more about the underlying mechanisms specific for the apical root fragments, SSH-based analysis was performed to identify tobacco metal-homeostasis genes differentially expressed in transgenic tobacco exposed to Cd.

Moreover, we looked in transgenic plants for differentially expressed Zn–Cd cross-homeostasis genes that could be associated with the Zn/Cd-dependent modifications of Zn/Cd/Fe uptake, accumulation, and distribution.

#### Cd concentration in the roots of plants used for SSH analysis

Following 11 d of treatment with 0.25 µM Cd, its concentration in the root samples from plants used for SSH analysis was 2-fold lower in transgenic than in WT plants (Supplementary Fig. S1). The same modification pattern in root Cd concentration due to *AtHMA4* expression in tobacco was detected after a short, 4 d exposure to 0.25 µM Cd (Siemianowski *et al.*, 2011). The Fe concentration did not differ from that in the WT, but the Zn concentration was lower (Supplementary Fig. S1).

#### Identification of genes with differential expression in transgenic tobacco

The quality and efficiency of the key steps of the SSH procedure are shown in Supplementary Protocol S1. Identified clones were deposited in the NCBI database under number LIBEST_028636. A total of 3450 cDNA clones with differential expression in transgenic roots were identified, of which 1650 had higher and 1800 had lower expression. To eliminate false clones prior to sequencing, the resulting subtractive clones were differentially screened by reverse northern blot analysis. Colony PCR was performed to amplify the insert within every clone. The PCR products from all 3450 clones were blotted onto nylon membranes in duplicate. The differentially expressed genes were identified by screening with either tester or driver cDNA as the probe. A representative northern blot is shown in Supplementary Protocol S1. In transgenic roots, northern blot analysis confirmed the presence of 527 and 501 clones with transcript levels higher and lower, respectively, than in the WT. Clones with a single insert >100 bp were sequenced and subjected to bioinformatics analysis to assign annotations. The final list of identified differentially expressed genes and new sequences that were categorized here based on defined similarity to genes from other organisms is available in (Supplementary Table S2. A total of 487 up-regulated (316 independent clones) and 485 down-regulated (354 independent clones) genes/sequences were identified in transgenic roots exposed to Cd. The focus of this study was on metal-homeostasis and related genes. Among transporters of ions and low molecular weight molecules, 32 clones (corresponding to 23 independent clones) were identified as up-regulated, and 37 clones (corresponding to 27 independent clones) as down-regulated. Within genes involved in cell wall modification processes, there were 89 up-regulated (48 independent clones) and 11 down-regulated (eight independent clones) Genes.

**Fig. 1.**
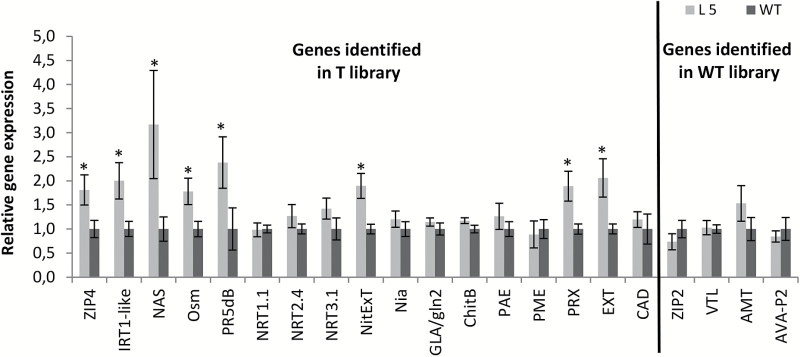
Confirmation of differential expression of 17 genes identified by SSH in the T library and four genes in the WT library. Analysis was performed on RNA used for construction of SSH libraries, isolated from roots of *AtHMA4*-expressing plants line 5 (L5) and the wild type (WT). Genes involved in the following processes were included in the analysis: Zn/Fe homeostasis (*ZIP4*, *IRT1*-like, *NAS*, *ZIP2*, and *VTL*), biotic/abiotic stress (*Osm* and *PR5dB*), nitrate metabolism (*NRT1.1*, *NRT2.4*, *NRT3.1*, *NitExT*, *Nia*, and *AMT*), cell wall metabolism (*GLA/gln2*, *ChitB*, *PAE*, *PME*, *PRX*, *EXT*, and *CAD*), and proton transport (*AVA-P2*). The clone signatures corresponding to individual genes are shown in Supplementary Table S3. Gene expression was normalizsed to the PP2A level. Values correspond to means ±SD (*n*=3); those significantly different from the WT (Student’s *t*-test) are indicated by an asterisk (*P*≤0.05).

Then, from among genes selected by SSH (Supplementary Table S2), for confirmation of differential expression, those involved in the regulation of Zn–Cd–Fe cross-homeostasis were chosen. This involvement could be direct (metal-homeostasis genes) or indirect (genes involved in cell wall metabolism, responses to biotic/abiotic stresses, and in nitrogen metabolism). Seventeen up-regulated (detected in the T library) and four down-regulated (detected in the WT library) genes were selected (Supplementary Table S3). The expression analysis was performed on RNA isolated from the material used for SSH analysis. Higher expression was confirmed for eight genes (Fig. 1). They represented several functional categories. Two Zn/Fe/Cd transporters were identified: the first was *NtZIP4* with 99% homology to *ZIP4* (ZRT-IRT-like Protein) from *N. tormentosiformis*. ZIP proteins mediate the transport of different metals, including Zn, Mn, Cd, and Fe (Ishimaru *et al.*, 2005; Sinclair and Krämer, 2012). The second was *NtIRT1*-like (Iron Regulated Transporter 1-like). It is known that *NtIRT1* encodes the Fe, Zn, Cd, and Mn uptake protein (Hodoshima *et al.*, 2007; Milner *et al.*, 2013). Moreover, *NtNAS* encoding nicotianamine synthase, involved in the biosynthesis of a metal chelator, nicotianamine, was also up-regulated. Nicotianamine is a non-proteinogenic amino acid that plays a key role in the regulation of Zn–Fe–Cd cross-homeostasis (Curie *et al.*, 2009). The next class included two osmotin genes involved in a plant’s response to biotic/abiotic stresses, *NtOsm* and *NtPR5dB*, and higher expression was confirmed for both. Five nitrate metabolism genes were identified by SSH as up-regulated, though real-time PCR analysis showed an expression level higher than in the WT only for *NtNitExT*. The last group included seven cell wall metabolism-related genes. However, a higher transcript level was confirmed for only two. One was *PrxSub2* (suberization-associated anionic peroxidase 2-like) involved in suberization (Valério *et al.*, 2004). The second, *NtExt*, encodes extensins implicated in fortification of the cell wall to respond to pathogens, wounding, and other environmental signals (Skinner and Gasser, 2009; Tenhaken, 2015).

Four down-regulated genes in transgenic plants were selected for analysis (Supplementary Table S3). However, expression analysis showed a slightly lower transcript level only for *NtZIP2* (Fig. 1).

#### AtHMA4 expression in tobacco modifies Zn, Cd, and Fe accumulation in a Zn/Cd supply-dependent fashion.

**Fig. 2.**
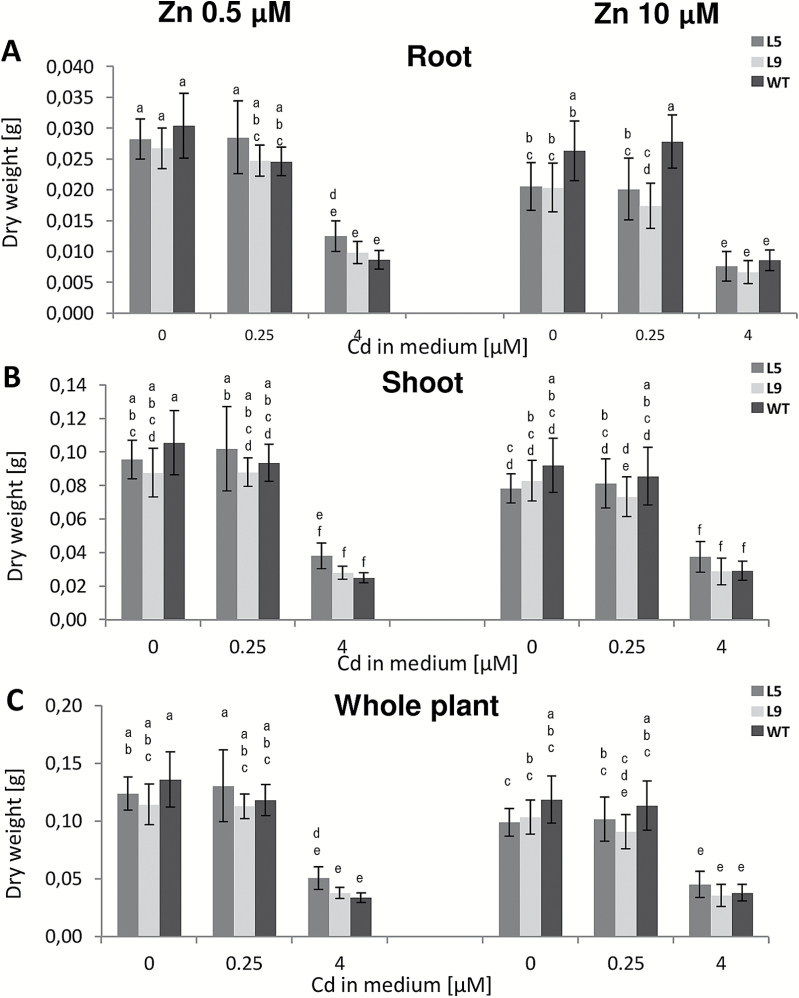
Dry weight of *AtHMA4*-expressing tobacco (line 5 and 9) and wild-type (WT) plants exposed to a range of Cd and Zn supply. Four-week-old plants were grown for 11 d in the presence of 0.25 µM or 4 µM Cd added to the control medium (0.5 µM Zn) or to the control medium supplemented with 10 µM Zn. (A) Dry weight of roots; (B) dry weight of shoots; (C) dry weight of entire plants. Values correspond to arithmetic means ±SD (*n*=5–10). For each trait, means followed by different letters are significantly different from each other according to one-way ANOVA followed by Tukey’s test (*P*<0.05) using the R program.

To determine to what extent Zn, Cd, and Fe accumulation and root/shoot distribution were affected in tobacco by expression of *AtHMA4*, and whether they depended on the tested combinations of Zn and Cd concentrations in the medium, two Cd concentrations (0.25 µM or 4 µM Cd) were added to the medium containing either 0.5 µM or 10 µM Zn. Exposure of both WT and transgenic plants to 0.25 µM Cd present in both types of media did not significantly affect the biomass of the roots and shoots as compared with the control conditions. In the presence of higher 4 µM Cd, dry weight was decreased, however, to the same extent for all tested plant lines (Fig. 2).

**Fig. 3.**
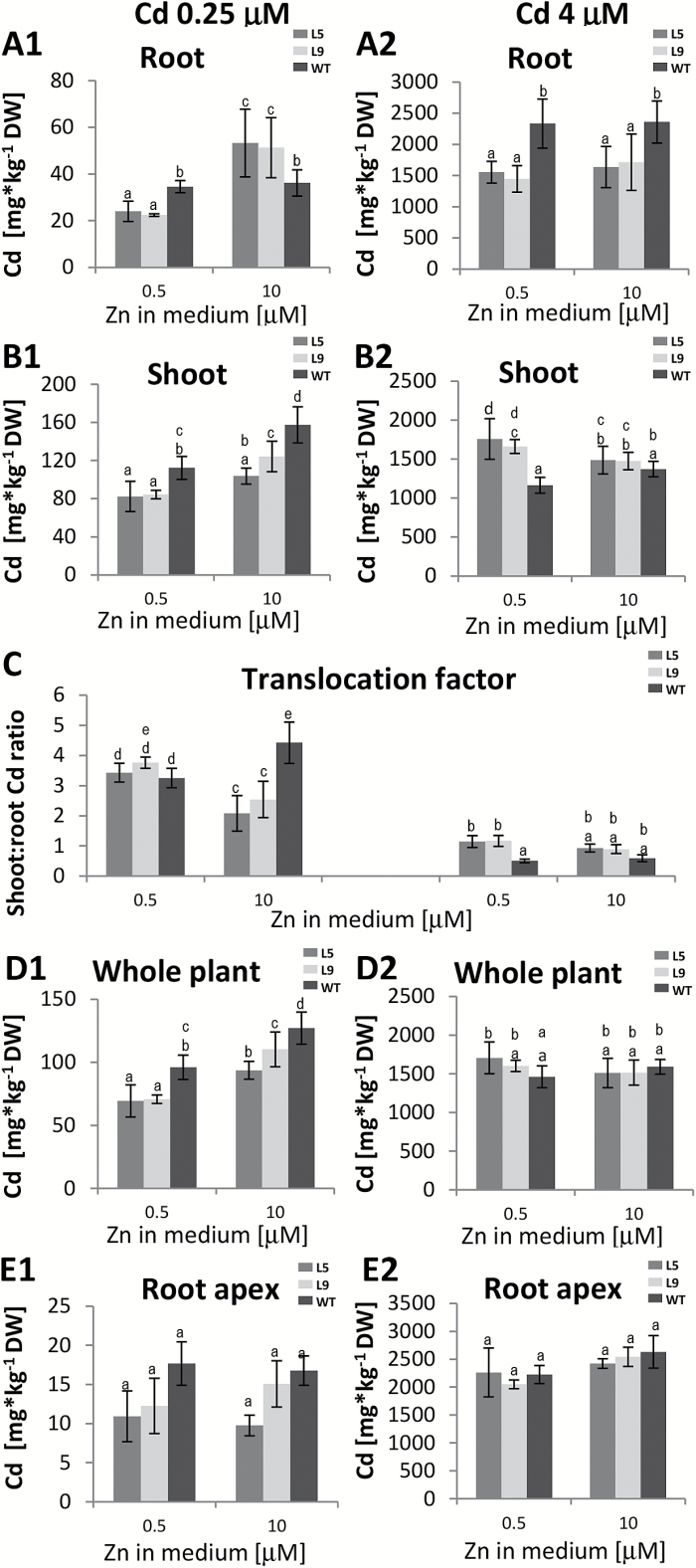
Cd concentration in *AtHMA4*-expressing tobacco (line 5 and 9) and wild-type (WT) plants exposed to a range of Cd and Zn supply. Four-week-old plants were grown for 11 d in the presence of 0.25 µM or 4 µM Cd added to the control medium (0.5 µM Zn) or to the control medium supplemented with 10 µM Zn. (A1, A2) Cd concentration in roots; (B1, B2) Cd concentration in shoots; (C) shoot:root Cd concentration ratio; (D1, D2) Cd concentration in entire plants; (E1, E2) Cd concentration in the 2.5 cm apical segments of the roots. Values correspond to arithmetic means ±SD (*n*= 5–10). For each trait, means followed by different letters are significantly different from each other according to one-way ANOVA followed by Tukey’s test (*P*<0.05) using the R program.

In WT plants, Cd accumulation depended on both Zn and Cd concentrations in the medium (Fig. 3). When exposed to 0.25 µM Cd, it was higher in shoots in the presence of 10 µM Zn than in the presence of 0.5 µM Zn (Fig. 3B1), and the higher shoot:root Cd ratio (Fig. 3C) indicated more efficient Cd translocation to shoots.

Expression of *AtHMA4* modified Cd accumulation but the pattern depended on the concentration of both Cd and Zn in the medium. In plants grown in medium containing 0.5 µM Zn, the concentration of Cd in shoots was lower than in the WT in the presence of 0.25 µM Cd, and higher at 4 µM Cd (Fig. 3B1, B2). Modification of the efficiency of Cd translocation to shoots due to *AtHMA4* expression was also Cd and Zn dependent (Fig. 3C). Furthermore, the whole-plant Cd concentration was lower in transgenic plants than in the WT upon exposure to 0.25 µM Cd (Fig. 3D1), indicating reduced Cd uptake. It is noteworthy that in the apical root segments from transgenic plants, the Cd concentration remained at the WT level (Fig. 3E1, E2), whereas in the whole roots it was lower for most of the treatments (Fig. 3A1, A2).

**Fig. 4.**
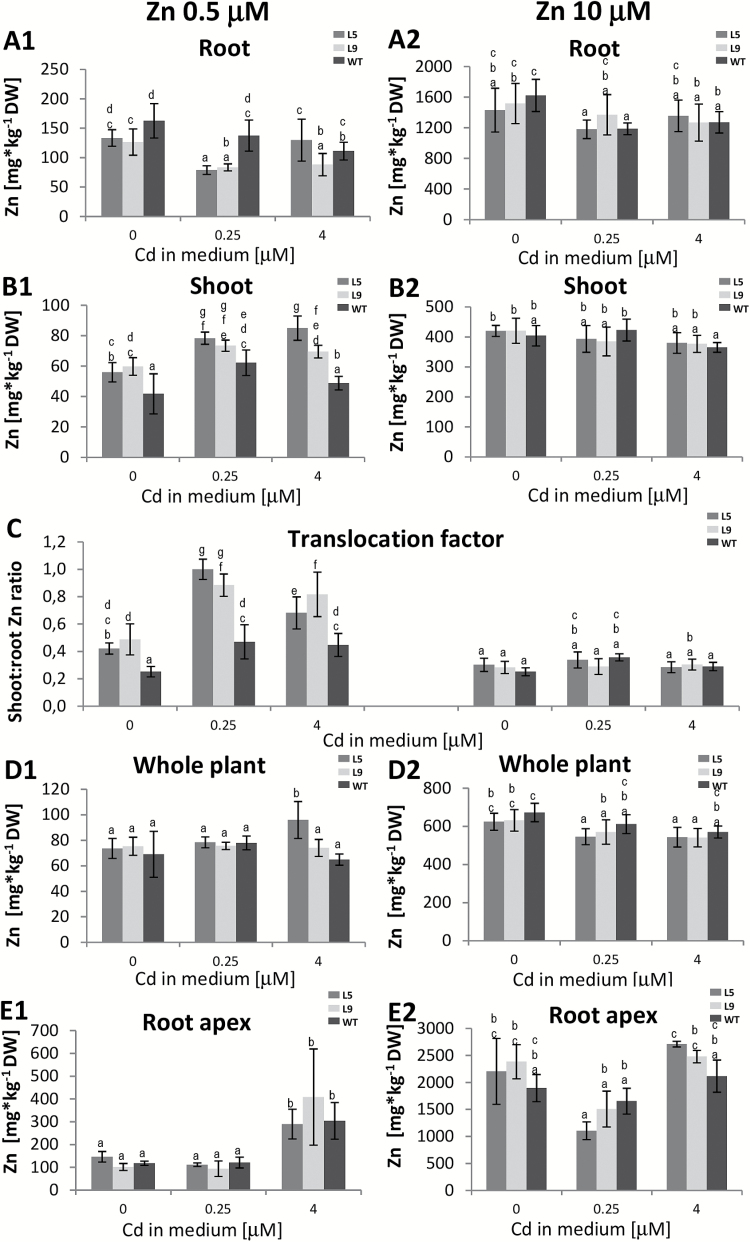
Zn concentration in *AtHMA4*-expressing tobacco (line 5 and 9) and wild-type (WT) plants exposed to a range of Cd and Zn supply. Four-week-old plants were grown for 11 d in the presence of 0.25 µM or 4 µM Cd added to the control medium (0.5 µM Zn) or to the control medium supplemented with 10 µM Zn. (A1, A2) Zn concentration in roots; (B1, B2) Zn concentration in shoots; (C) shoot:root Zn concentration ratio; (D1, D2) Zn concentration in entire plants; (E1, E2) Zn concentration in the 2.5 cm apical segments of the roots. Values correspond to arithmetic means ±SD (*n*= 5–10). For each trait, means followed by different letters are significantly different from each other according to one-way ANOVA followed by Tukey’s test (*P*<0.05) using the R program.

In WT plants, Zn accumulation also depended on the Cd concentration in the medium (Fig. 4). Interestingly, in plants grown at 0.5 µM Zn in the presence of Cd, facilitation of Zn translocation to shoots was observed (Fig. 4C).

Expression of *AtHMA4* changed Zn accumulation, but most significantly in plants grown at 0.5 µM Zn in the medium compared with 10 µM Zn (Fig. 4). Zn translocation to shoots was facilitated, primarily in the presence of Cd (Fig. 4C). Expression of *AtHMA4* did not substantially modify the Zn concentration in the apical fragments (Fig. 4E1, E2).

**Fig. 5.**
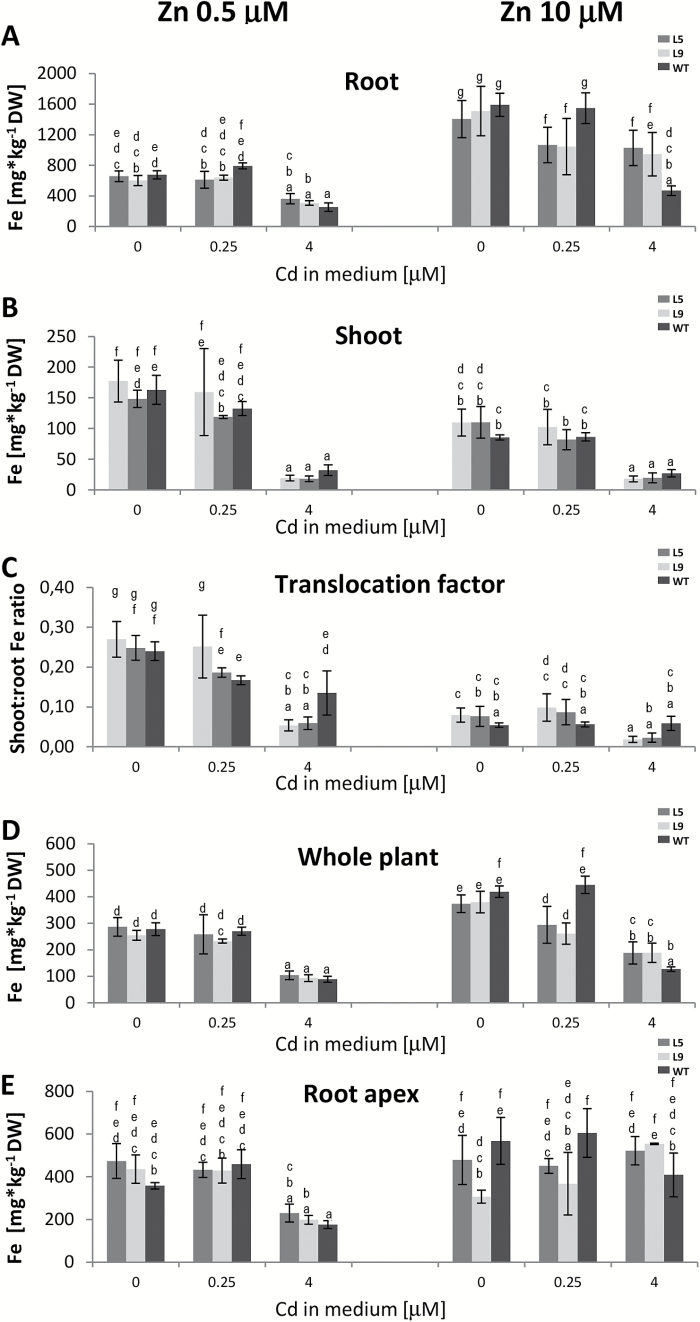
Fe concentration in *AtHMA4*-expressing tobacco (line 5 and 9) and wild-type (WT) plants exposed to a range of Cd and Zn supply. Four-week-old plants were grown for 11 d in the presence of 0.25 µM or 4 µM Cd added to the control medium (0.5 µM Zn) or to the control medium supplemented with 10 µM Zn. (A) Fe concentration in roots; (B) Fe concentration in shoots; (C) shoot:root Fe concentration ratio; (D) Fe concentration in entire plants; (E) Fe concentration in the 2.5 cm apical segments of the roots. Values correspond to arithmetic means ±SD (*n*=5–10). For each trait, means followed by different letters are significantly different from each other according to one-way ANOVA followed by Tukey’s test (*P*<0.05) using the R program.

In the WT, the Fe concentration strongly depended on Zn as well as Cd concentrations in the medium (Fig. 5). Exposure to Cd reduced the Fe concentration in the whole roots, apical parts of roots, and in shoots of the WT and transgenic plants, primarily at the higher (4 µM) Cd concentration (Fig. 5A, B , D, E). Moreover, in the presence of higher (10 µM) Zn, translocation of Fe to shoots was less efficient in all the tested media (Fig. 5C). The expression of *AtHMA4* did not alter Fe accumulation except for an increase at 10 µM Zn+4 µM Cd (Fig. 5A).

To summarize, the results indicate metal status-dependent effects of the expression of the transgene on the metal accumulation pattern. To explore this phenomenon in more detail, the transcript abundance of selected metal-homeostasis genes was evaluated under the different media tested.

### Differential expression of metal-homeostasis genes accompanies Zn/Cd supply-dependent modifications of Zn, Cd, and Fe accumulation in transgenic tobacco

For analysis, the following genes tested in the previous stage of the research (Fig. 1) were used: *NtZIP2*, *NtZIP4*, *NtIRT1A*, *NtNAS*, and *NtVTL*. Their sequences were aligned with *A. thaliana* homologues for best annotated hits and compared with annotated and EST sequences from *Nicotiana* species (Supplementary Dataset S1).

**Fig. 6.**
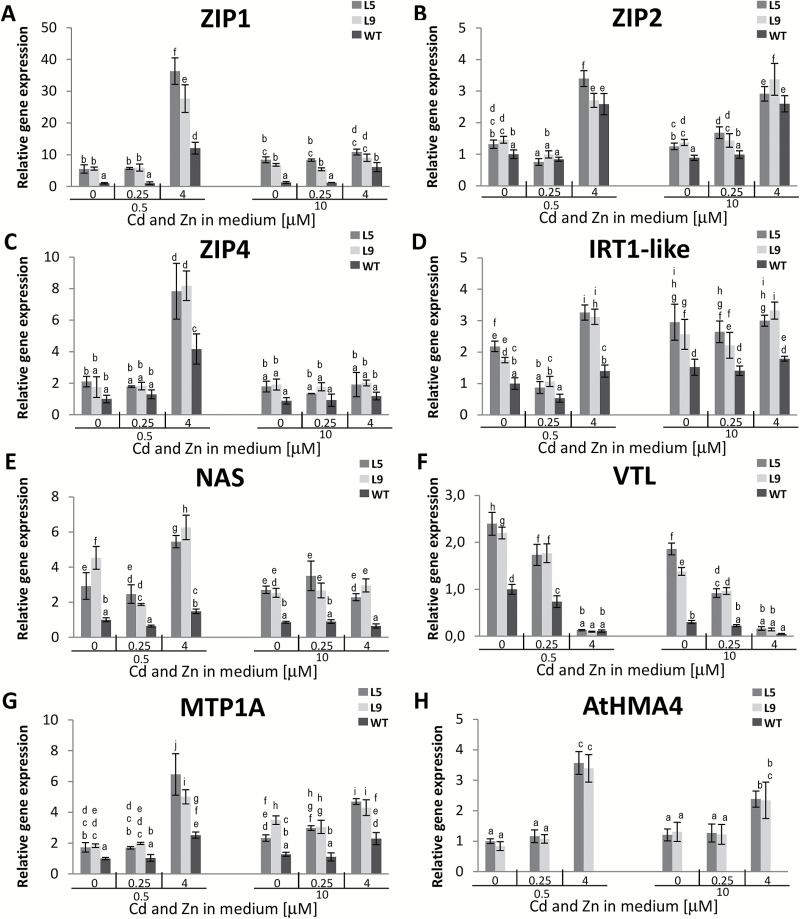
Expression of *NtZIP1* (A), *NtZIP2* (B), *NtZIP4* (C), *NtIRT1*-like (D), *NtNAS* (E), *NtVTL* (F) *NtMTP1A* (G), and *AtHMA4* (H) in 2.5 cm apical parts of roots of *AtHMA4*-expressing tobacco (line 5 and 9) and the wild type (WT) exposed to a range of Cd and Zn concentrations. Four-week-old plants were grown for 11 d in the presence of 0.25 µM or 4 µM Cd added to the control medium (0.5 µM Zn) or to the control medium supplemented with 10 µM Zn. Gene expression was normalized to the PP2A level. Values correspond to means ±SD (*n*=3); For each trait, means followed by different letters are significantly different from each other according to one-way ANOVA followed by Tukey’s test (*P*<0.05) using the R program.

In WT plants, the expression of *NtZIP1*, *NtZIP2*, and *NtMTP1A* was enhanced in the presence of high (4 µM) Cd, but for *NtZIP4* only at 0.5 µM Zn+4 µM Cd (Fig. 6A–C, G). Their transcript level was modulated in transgenic plants, except for *NtZIP2* (Fig. 6B). For *NtZIP1* it was 2- to 6-fold higher than in the WT under all applied conditions except at 10 µM Zn+4 µM Cd (Fig. 6A), whereas for *NtZIP4* specifically at 0.5 µM Zn+4 µM Cd (Fig. 6C). The expression of *NtMTP1A* significantly increased to a similar level under all treatments (Fig. 6G).

*IRT1* also belongs to the ZIP family. In WT tobacco, the amount of *NtIRT1*-like transcript was at a comparable level in all tested media, except for a decrease at 0.5 µM Zn+0.25 µM Cd (Fig. 6D). In transgenic plants it remained unmodified only at 0.5 µM Zn+0.25 µM Cd, whereas under all other tested conditions it was higher.

The expression of *NtNAS* in the WT was similar in Zn/Cd treatments, except that it was higher in plants grown at 0.5 µM Zn+4 µM Cd. In transgenic plants, it was significantly elevated under all tested conditions (Fig. 6E).

The expression of *NtVTL* significantly depended on the concentrations of Zn and Cd in both WT and transgenic plants (Fig. 6F). In the WT its expression was strongly down-regulated by Cd and also by high Zn. In transgenic plants, it was up-regulated in all media tested, except in plants exposed to 4 µM Cd.

Interestingly, even though *AtHMA4* was expressed in tobacco under the constitutive 35S promoter, its expression in the roots was significantly higher at 4 µM Cd compared with other treatments (Fig. 6H).

To summarize, *AtHMA4* expression in tobacco significantly modified expression of endogenous metal-homeostasis genes. The factors that differentiate the expression are, first, the concentration of Zn in the medium, and, secondly, a high (4 µM) Cd concentration, which is accompanied by increased expression of the transgene.

### Assessment of the regulation of identified metal-homeostasis genes by Zn and Fe deficiency

The physiological function of *NtZIP1*, *NtZIP2*, *NtZIP4*, *NtIRT1*-like, *NtNAS*, *NtVTL*, and *NtMTP1A* in tobacco, and the regulation of these genes by Zn and Fe, is not known for the majority of them. Their homologues in other organisms are primarily Zn and/or Fe deficiency inducible. Therefore, as a next step we have determined their expression in tobacco in plants grown under Zn and Fe deficiency for 11 d (the same period as exposure to Cd).

**Fig. 7.**
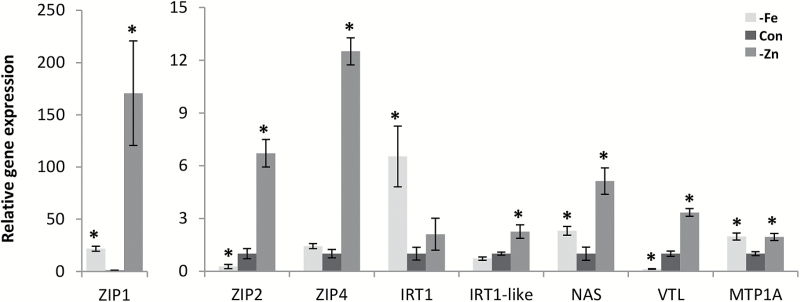
Expression of selected genes in 2.5 cm apical parts of roots of wild-type plants exposed to Zn or Fe deficiency. Four-week-old plants were grown for 11 d under control condition (Con), under Zn deficiency (–Zn), or under Fe deficiency (–Fe). Gene expression was normalized to the PP2A level. Values correspond to means ±SD (*n*=3); those significantly different from the control conditions (Student’s *t*-test) are indicated by an asterisk (*P*≤0.05).

All tested genes were regulated by Zn and/or Fe deficiency to a different extent (Fig. 7). All of them appeared to be Zn deficiency inducible. Strong up-regulation was found for *NtZIP1*, also for *NtZIP2* and *NtZIP4*, and to a lesser extent for *NtNAS1*, *NtVTL*, *NtIRT1*-like, and *NtMTP1A*. Fe deficiency-inducible genes were *NtZIP1*, *NtIRT1* (but not *NtIRT1*-like), *NtNAS1*, and *NtMTP1A*. Fe deficiency down-regulated the expression of *NtZIP2* and *NtVTL*.

### AtHMA4 expression in tobacco increased Zn concentration in the root apoplastic wash fluid

**Fig. 8.**
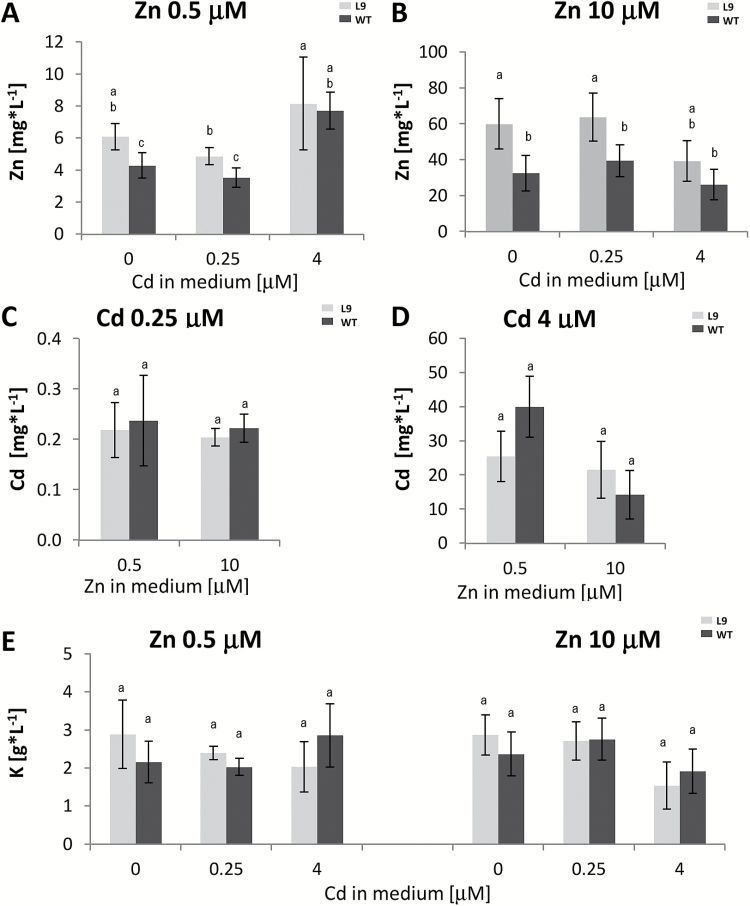
Zn, Cd, and K concentrations in root apoplastic fluid. Apoplastic fluid was isolated from roots of 4-week-old plants grown for 11 d in the presence of 0.25 µM or 4 µM Cd added to the control medium (0.5 µM Zn) or to the control medium supplemented with 10 µM Zn. Zn concentrations (A, B); Cd concentrations (C, D); K concentrations (E). Values correspond to means ±SD (*n* = 3); those significantly different from the WT (Student’s *t*-test) are indicated by an asterisk (*P*≤0.05).

In *A. thaliana,* AtHMA4 exports Zn and Cd from the symplast to the apoplast (Mills *et al.*, 2003, 2005) and, when ectopically expressed in tobacco, overloads the apoplast of the leaves with Zn (Siemianowski *et al.*, 2013). Here it was shown that in the roots of tobacco expressing *35S::AtHMA4* grown in a medium containing 0.5 µM and 10 μM Zn, with and without 0.25 μM Cd, the Zn concentration in the AWF was higher than in the WT (Fig. 8A, B). However, in the presence of 4 μM Cd, Zn in the transgenic roots was exported to the apoplast less efficiently, and its concentration in both tested lines was not significantly different (Fig. 8A). *AtHMA4* expression did not significantly modify the Cd concentration in the roots’ AWF (Fig. 8C, D). The K concentration used as an indicator for cytoplasmic contamination was at a comparable level for the studied lines (Fig. 8E).

## Discussion

It has already been shown in numerous plant species that a metal’s root/shoot distribution pattern depends on its concentration in the medium, and on the medium’s overall mineral composition (Krupa *et al.*, 2002). The molecular basis of this phenomenon, apparently involving metal cross-homeostasis mechanisms, is poorly understood. Moreover, studies to date demonstrate that it is difficult to engineer a desired metal root/shoot distribution pattern that would remain stable upon exposure to low, medium, and high metal concentrations (Korenkov *et al.*, 2007; Wojas *et al.*, 2009; Antosiewicz *et al.*, 2014). Herein we demonstrate that in tobacco, *AtHMA4* expression modified Zn and Cd, and to a lesser extent Fe, root/shoot distribution, and that its pattern depended on the mutual relationship between the Zn and Cd concentrations in the growth medium. According to current knowledge, the apical part of the root plays a pivotal role in metal uptake and regulation of root to shoot transfer, in which metal uptake, efficiency of radial transport, and loading into xylem vessels are key processes (Palmgren *et al.*, 2008; Puig and Peñarrubia, 2009). Therefore, we focused on genes specifically expressed only in the apical root region (and not in the entire roots), and identified seven metal-homeostasis genes (*NtZIP1*, *NtZIP2*, *NtZIP4*, *NtIRT1*-like, *NtNAS*, *NtVTL*, and *NtMTP1A*) whose expression pattern: (i) in the WT depended on combinations of Zn and Cd in the medium; and (ii) in transgenics also depended on combinations of Zn and Cd in the medium, but differed from the WT expression pattern.

It is noteworthy that there were differences in the accumulation patterns of Zn, Cd, and Fe in the apical root segments and whole roots when plants subjected to the same combination of low/high concentrations of Zn and Cd were compared (Figs 3–5). This suggests that the detected alterations in the expression pattern of metal transport genes identified in the root apices from plants grown in the tested medium variants probably contributed to the changes found in metal root/shoot partitioning, and that its basal part has a yet unidentified role in this process.

### Zn–Cd–Fe cross-homeostasis in wild-type tobacco

#### Cd concentration-dependent alterations of Zn–Cd–Fe cross-homeostasis

In the WT, the presence of Cd (primarily at 4 µM) decreased Fe root to shoot translocation and its concentration in both organs, and moderately increased translocation of Zn. The efficiency of Cd translocation to shoots also depended on the Cd concentration in the medium, and was lower in plants grown at the higher 4 µM Cd. These changes were accompanied by higher expression of *NtZIP1*, *NtZIP2*, and *NtMTP1A*, and lower expression of *NtVTL1* in the root apical segments (Figs 3, 5, 6).

ZIPs are transporters with broad substrate specificity, including Zn, Fe, and Cd (Guerinot, 2000). In tobacco their function remains largely unknown. The only up to date study was performed on NtZIP1 and suggests its involvement in Fe transport (Sano *et al.*, 2012). The most extensive analysis has been performed on Arabidopsis. The tonoplast- localized AtZIP1 and plasma membrane-localized AtZIP2 were shown to mediate Zn and Mn transport. Moreover, the Zn transport mediated by both transporters (and also by *MtZIP1*) was significantly inhibited by Cd, indicating that it could also be a substrate (Grotz *et al.*, 1998; Stephens *et al.*, 2011; Milner *et al.*, 2013). It was suggested that AtZIP1 and AtZIP2 could contribute to controlling root to shoot translocation of metals by regulating their radial movement to xylem parenchyma for subsequent xylem loading (Milner *et al.*, 2013). More analyses are thus necessary to determine the substrates for NtZIP1 and NtZIP2 to prove their contribution to the regulation of Zn/Cd/Fe root/shoot partitioning.

The next gene with enhanced expression on exposure to 4 µM Cd was *NtMTP1A.* Its up-regulation by Cd was also shown by Bovet *et al.* (2006). It encodes a tonoplast-localized protein, which mediates loading of Zn and Co (but not Cd) into vacuoles. Thus, there was a suggestion that *NtMTP1A* participates in regulating the pool of Zn available for translocation to the shoots (Shingu *et al.*, 2005; Bazihizina *et al.*, 2014).

The opposite effect, down-regulation in the presence of 4 µM Cd, was detected for *NtVTL* (Fig. 6), the first *VTL*-related sequence identified in tobacco (Supplementary Dataset S1). In Arabidopsis, *AtVTL1* encodes the tonoplast transporter mediating Fe sequestration in vacuoles. Its involvement in Fe–Zn cross-homeostasis has been suggested by the study on the *pye-1* knock-out mutant showing that it is under the control of the PYE regulatory network involved in regulating *AtNAS4*, *AtZIF1*, and *AtFRO3* (Gollhofer *et al.*, 2014). Thus, it is not excluded that *NtVTL* could contribute, directly or indirectly, to the regulation of metal root/shoot partitioning by keeping the amount of metals in vacuolar stores under control.

Expression analysis has shown that in WT plants, all seven of the examined genes (*NtZIP1*, *NtZIP2*, *NtZIP4*, *NtIRT1*-like, *NtNAS*, *NtVTL*, and *NtMTP1A*) were up-regulated by long-term Zn deficiency (Fig. 7). On the other hand, Fe deficiency up-regulated *NtZIP1* and *NtMTP1*, and down-regulated *NtZIP2* and *NtVTL* expression (Fig. 6). These results are in agreement with the study on BY-2 tobacco cells that indicated down-regulation of *NtZIP1* by Fe excess (Sano *et al.*, 2012), and with known regulation of *AtVTL1* by Fe deficiency in Arabidopsis (Gollhofer *et al.*, 2014). Thus enhanced expression of *NtZIP1*, *NtZIP2*, and *NtMTP1A* and decreased expression of *NtVTL* in the apical segments of roots grown at 4 µM Cd might suggest a low Zn and/or Fe status at the cellular level, although in these root fragments a decrease in the total metal concentration was noted only for Fe, and only in segments from roots exposed to 4 µM Cd+0.5 µM Zn (Fig. 5).

Interestingly, at just that medium composition, up-regulation of *NtZIP4* was noted in the WT (Fig. 6C); it was accompanied not only by a decreased Fe concentration in the root apices, but also by an increased Zn concentration (Figs 4E1, 5E). In tobacco, *NtZIP4* was shown to be Zn and Fe deficiency responsive (Fig.7). In Arabidopsis, *AtZIP4* is inducible by Zn deficiency, but also by Cd exposure (Assunção *et al.*, 2010; Jain *et al.*, 2013). Thus, here the exposure to 4 µM Cd could contribute to the detected increased expression of *NtZIP4.*

#### Zn concentration-dependent alteration of Zn–Cd–Fe cross-homeostasis

In the WT, the medium Zn concentration was also a factor contributing to changes in Zn, Fe, and Cd root/shoot partitioning. The efficiency of Zn and Fe translocation to shoots was higher at the lower concentration of 0.5 µM Zn (Figs 4C, 5C). For Cd, however, it was lower but only when plants exposed to 0.25 µM Cd were compared (Fig. 3C).

The most significant differences in the expression of the examined genes between two variants of the medium containing 0.5 µM or 10 µM Zn (with and without two Cd concentrations) were found for *NtVTL*. Its expression in the WT was higher on 0.5 µM Zn relative to 10 µM Zn and depended on the presence of Cd (Fig. 6F). The detected expression pattern suggests a key role for *NtVTL* in the regulation of Fe–Cd–Zn cross-homeostasis at a range of combinations of Zn/Cd concentrations in the medium, and a contribution to the regulation of the efficiency of transfer of metals to shoots. To verify this hypothesis, *NtVTL* has to be cloned and functionally characterized.

The opposite relationship, lower expression at 0.5 µM Zn compared with 10 µM Zn, was noted for *NtIRT1*-like (Fig. 6D). The *NtIRT1*-like partial sequence identified in this study has 96% nucleotide homology to *NtIRT1* (AB263746.1; Supplementary Dataset S1). Fe deficiency-inducible *NtIRT1* is the only *IRT* gene cloned in tobacco. It belongs to the Strategy I Fe deficiency uptake system (Yoshihara *et al.*, 2006; Hodoshima *et al.*, 2007). Here, in tobacco exposed for 11 d to Fe deficiency, expression of *NtIRT1*-like remained at the control level, whereas *NtIRT1* was highly up-regulated (Fig. 7). The identified NtIRT1-like partial amino acid sequence covers the sequence of NtIRT1 from position 257 up to the end of the protein (355 amino acidss in total). This includes transmembrane (TM) domains VI–VIII, cytosolic loops between the V–VI and VII–VIII TMs, the extracellular region between the VI–VII TM, and the C-terminus (Supplementary Dataset S1; Rogers *et al.*, 2000; Hodoshima *et al.*, 2007). The identified NtIRT1-like sequence differs from NtIRT1 only at position 271. For AtIRT1, key amino acid residues decisive in metal selectivity were identified in the first third of the protein (Rogers *et al.*, 2000). Until the entire *NtIRT1*-like sequence is known, it is not possible to draw conclusions about why it is not regulated by Fe deficiency (typical for *IRT1* genes). It could result from a difference in the promoter region or from altered selectivity of the protein itself due to substitution of the amino acids key for substrate specificity. On the other hand, the level of up-regulation of both *NtIRT1*-like and *NtIRT1* by low Zn was similar (Fig. 7).

To summarize, the results indicate a contribution of the identified tobacco genes to the Zn/Cd supply-dependent modifications of Zn, Cd, and Fe root/shoot distribution in WT plants through their metal status-dependent co-ordinated responses. As their role in tobacco is largely unknown, future cloning and detailed characterization will verify the above suggestions. These Zn/Cd supply-specific expression patterns constitute a molecular background, distinct at applied variants of tested combinations of low/high concentrations of Zn and Cd, against which expression of *AtHMA4* takes place.

### Expression of *AtHMA4* in tobacco alters Zn/Cd supply-dependent Zn–Cd–Fe cross-homeostasis

Considering the role of *AtHMA4* in Arabidopsis in loading Zn (and additionally Cd) into xylem vessels in roots, the expectation was that its expression in tobacco might increase translocation of both metals to shoots. Indeed, the concentration of Zn and Cd in shoots increased as well as the efficiency of metal root to shoot translocation; however, not at all medium compositions (Figs 3–5). Changes in Zn/Cd/Fe distribution (as compared with the WT), which depended on the combinations of low/high concentrations of Zn/Cd in the medium, probably resulted from involvement of tobacco metal cross-homeostasis mechanisms.

Interestingly, the expression of identified tobacco metal-homeostasis genes in roots of transgenic plants grown on six tested differnt media was predominantly higher than in the WT (Fig. 6), and all genes appeared to be Zn deficiency inducible (Fig. 7). This points to Zn deficiency status as an important factor contributing in transgenic tobacco to changes in the expression of the identified metal-homeostasis genes. This is in agreement with the previous study performed on leaves which showed that due to ectopic expression of *AtHMA4* in tobacco, Zn export mediated by AtHMA4 protein leads to overloading of the apoplast with Zn, and generates the status of Zn deficiency (Siemianowski *et al.*, 2013). Here we showed that also in roots of *AtHMA4*-expressing plants, the Zn concentration in the apoplastic fluid was higher than in the WT except when exposed to high 4 µM Cd (Fig. 8A, B). It is not excluded that a high concentration of Cd reduces the AtHMA4-dependent transport of Zn, despite a higher expression level of AtHMA4 at 4 µM Cd compared with other tested medium variants (Fig. 6H). It should be emphasized that, in contrast to leaves, roots of transgenic and WT plants under comparison are immersed in the medium of the same composition, which probably decreases the difference in metal concentration in the apoplastic fluid.

Detected modifications in the expression pattern of *NtZIP2*, *NtMTP1A*, and *NtNAS* in transgenic plants were only quantitative in all the different medium variants tested (the transcript levels of a given gene increased proportionately relative to the WT Fig. 6). However, for *NtZIP4*, *NtVTL*, *NtZIP1*, and *NtIRT1*-like, changes were highly specific at a given medium composition. The most significant change was detected for *NtZIP4*. Its expression was 2-fold higher than in the WT just in the medium containing 0.5 µM Zn+4 µM Cd, whereas for all other treatments it remained at the WT level (Fig. 6C). Thus, *NtZIP4* responding to the specific metal status generated due to *AtHMA4* expression at that experimental combination accompanies increased Cd and Zn and reduced Fe concentration in the shoots. Thus, *NtZIP4* might contribute to the modification of Zn/Cd/Fe root/shoot partitioning in transgenic tobacco grown at 4 µM Cd (Figs 3–5).

The changes in the expression of *NtVTL* were different from those noted for *NtZIP4.* The *NtVTL* expression in transgenic plants was significantly higher at 0.5 µM Zn than at 10 µM Zn (with or without 0.25 µM Cd), but not in both medium variants containing 4 µM Cd (Fig. 6F). The more marked increase in *NtVTL* expression at 0.5 µM Zn (with or without 0.25 µM Cd) accompanied more efficient translocation of Zn to shoots and less efficient translocation of Cd (Figs 3, 4). On the other hand, exposure to 4 µM Cd significantly reduced the Fe concentration, which corresponded to lower expression of *NtVTL*, shown to be not only Zn deficiency inducible, but also down-regulated by low Fe (Fig. 7). Next, a substantial increase of *NtZIP1* was noted primarily in plants grown on medium with and without 0.25 µM Cd at both 0.5 µM and 10 µM Zn in the medium (Fig. 6A). Thus *NtZIP1* seems not to participate in the regulation of the metal partitioning at high 4 µM Cd exposure.

Finally, the transcript abundance of *NtIRT1*-like increased in transgenic plants at all combinations of Zn/Cd concentrations except 0.5 µM Zn+0.25 µM Cd (Fig. 6D). Interestingly, under this condition, Cd uptake in transgenic plants was reduced (Fig. 3A1, B1, D1). Considering the putative role of *NtIRT1*-like in Cd uptake, which might depend on the concentration of other competitive cations, including Zn (similarly to *NtIRT1*; Hodoshima *et al.*, 2007) it is not excluded that it could contribute to the detected lower Cd in transgenic plants (Fig. 3D1). The expression of *AtHMA4* in tobacco leads to an increased concentration of Zn in the roots’ apoplastic fluid, also in the presence of 0.25 µM Cd (Fig. 8A, B).

In summary, improved understanding of the molecular background of the regulation of Zn/Cd/Fe root/shoot partitioning in transgenic plants that depends on the Zn/Cd concentration in the medium (and differs from the WT) is crucial for future engineering of metal accumulation in plants. The importance of the current research lies in identification of the co-ordinated contribution of several endogenous tobacco metal-homeostasis genes to this phenomenon in *AtHMA4*-expressing tobacco plants. Genes identified by SSH remain largely uncharacterized in tobacco, and five of the seven tested genes had not been previously cloned (Supplementary Dataset S1). Their homology to other organisms indicates that encoded proteins are probably localized to different membranes of various organelles. Co-ordinated responses of the identified genes to six combinations of low/high concentrations of Zn and Cd were very specific in transgenics for each medium composition, and included metal import (*NtZIP1*, *NtZIP2*, *NtZIP4*, *NtIRT1*-like, and *VTL*), metal export from the cytoplasm to vacuoles (*NtMTP1A* and *NtVTL*), and the regulation of the nicotianamine level (*NAS*). Among them, the most specific response (medium containing 4 µM Cd+0.5 µM Zn) was noted for *NtZIP4.* In contrast, *NtVTL* was the most broadly responsive to all variants of Zn and Cd in the medium, thus it is a candidate gene for the regulation of Zn/Cd supply-dependent modification of Zn/Cd root/shoot distribution.

## Supplementary data

Supplementary data are available at *JXB* online.

Table S1. List of primers.

Table S2. List of cDNA clones identified by SSH.

Table S3. List of genes selected for confirmation of differential expression.

Figure S1. Cd, Zn, and Fe concentration in roots used for SSH analysis.

Protocol S1. Supplementary protocol for SSH procedures.

Dataset S1. Alignments of sequences of metal-homeostasis genes used for expression analysis with homologues from other plants

Supplementary Data
