# Supplementary material for: The ratio of Zn to Cd supply as a determinant of metal-homeostasis gene expression in tobacco and its modulation by overexpressing the metal exporter AtHMA4
Source: J Exp Bot. 2016 Oct 17;67(21):6201–14. doi: 10.1093/jxb/erw389 (PMC5100030; doi:10.1093/jxb/erw389)
Supplement: Supplementary Data [file supp_67_21_6201__index.html]

The ratio of Zn to Cd supply as a determinant of metal-homeostasis gene expression in tobacco and its modulation by overexpressing the metal exporter AtHMA4 — The ratio of Zn to Cd supply as a determinant of metal-homeostasis gene expression in tobacco and its modulation by overexpressing the metal exporter AtHMA4 — Supplementary Data 

# The ratio of Zn to Cd supply as a determinant of metal-homeostasis gene expression in tobacco and its modulation by overexpressing the metal exporter AtHMA4

## Supplementary Data

Data files

- supplementary\_figure\_S1.pdf - Supplementary Data
- supplementary\_table\_S1.pdf - Supplementary Data
- supplementary\_table\_S2.xlsx - Supplementary Data
- supplementary\_table\_S3.xlsx - Supplementary Data
- supplementary\_protocol\_S1.pdf - Supplementary Data
- supplementary\_dataset\_S1.pdf - Supplementary Data
