# Supplementary material for: The ratio of Zn to Cd supply as a determinant of metal-homeostasis gene expression in tobacco and its modulation by overexpressing the metal exporter AtHMA4
Source: J Exp Bot. 2016 Oct 17;67(21):6201–14. doi: 10.1093/jxb/erw389 (PMC5100030; doi:10.1093/jxb/erw389)
Supplement: Supplementary Data [file supp_erw389_supplementary_figure_S1.pdf]

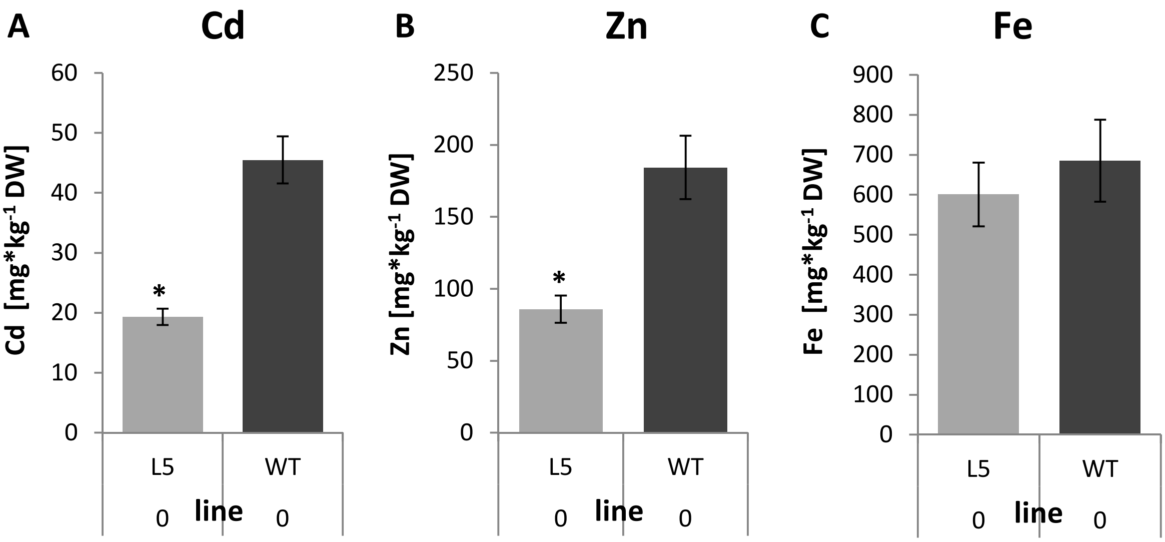

Figure S1: Cd, Zn and Fe concentration in roots used for SSH analysis. 4-week-old *AtHMA4*-expressing tobacco (line 5) and wild type (WT) plants were grown for 11 days in the presence of 0.25 µM Cd. Cd concentration (A), Zn concentration (B), Fe concentration (C). Values correspond to arithmetic means ±SD (n=11) from one independent running replicate experiments. Values correspond to means ± SD (n=11) from one independent running replicate experiments; those significantly different from wild type (WT) (Student's t-test) are indicated by asterisk (P≤0.05).
