# Supplementary material for: The ratio of Zn to Cd supply as a determinant of metal-homeostasis gene expression in tobacco and its modulation by overexpressing the metal exporter AtHMA4
Source: J Exp Bot. 2016 Oct 17;67(21):6201–14. doi: 10.1093/jxb/erw389 (PMC5100030; doi:10.1093/jxb/erw389)
Supplement: Supplementary Data [file supp_erw389_supplementary_table_S1.pdf]

Table S1: List of primers

gene	primers sequences	lenght of amplicon
NtAMT	F: GTACCAATGGGCTTTTGCT<br>R: CAAAACCAATGAGAAACAACC	138
NtAVA-P2	F: ACAGCGAAAAGTGGGGTAGG<br>R: CCAAAACTCCAGCCATAACAAC	97
NtChitB	F: CACCAAAACCTTCTTGCCACG<br>R: CCTCCTGTAAAACCCAATGCGA	176
NtCAD	F: TACAAGGCTGTGAGAATACTAAAC<br>R: ATTTCAAAATCGTCACCAACATCC	167
NtEXT	F: TAGCTCTCTCCCTGTAAAACC<br>R: CCTTCGACATATTTTCCTTCC	135
NtGLA/gln2	F: TAGCCCAAGAAAGCCTGGAC<br>R: CTGTCCCAAACTCCACCAGA	159
NtIRT1	F: CGCAATAACAACTCCATTCG<br>R: AAGCCATATAGATCAGAAGGC	134
NtIRT1-like	F: CTTCTTCGCAGTAACAACC<br>R: AGCCATGTAAATAAGAAGACC	139
NtMTP1A	F: GTGACGGTGAAGGTGTGC<br>R: CATGACCCCTATGCTCTGA	113
NtNAS	F: CTTGCTTGAGTTCAACATCC<br>R: AGAGAAGTGAGAGGGAGAGG	93
NtNia	F: CAGAAGCCATTTTGAGAGAAC<br>R: GATTAACAGCAAATTGAATCATAGG	96
NtNitExT	F: GTCATTGTCGCTGCCATTAG<br>R: GGAGAGGCTGTGATTGTG	101
NtNRT1.1	F: GTATTGCAGTTGGCTTAATCC<br>R: CTACTAGCAAGAATTGTGGG	162
NtNRT2.4	F: GCTCAAAATACTCGACAGC<br>R: CATAATAATGTTCCTCACTTCC	156
NtNRT3.1	F: GATGTTTCTGCTTCACACAGG<br>R: CTTTCTTGTAGCTTGAGTCTGC	115
NtOsm	F: CTTGGCCGAATACGCATTGG<br>R: CTAAGTTCTCGGGGACATTCAC	171
NtPAE	F: CATAAGACGCTACCAAGAACG<br>R: CAGCAGGAAAAGAGGGGTCT	102
NtPME	F: GGACCTGGAGCTGGAATTAGT<br>R: CCACTGTGAATTTCGTTGCC	85
NtPP2A	F: GCACATTCATTCAGTTTGAACC<br>R: GTAGCATATAAAGCAGTCAGC	142
NtPR5dB	F: GTGTTTGAGGTCCACAACAACTG<br>R: GTTCGAGACGTTTGCCACCC	79
NtPRX	F: CCTAAATAAACAACGTATGGCTG<br>R: GATTCTCCGCTCAAGACGAG	84
NtVTL	F: GAGACAAAACAACAGGAGGAC<br>R: GCAATAGCTGAGGCTACG	109

NtZIP1	F: TGGTGGCTCAGTCTGGAGAT	94
	R: CGAAGGAGCTCAGAACTGGAA	
NtZIP2	F: CACCATGTTTAGTGACTGC	136
	R: CTTGAGAAAAGGATTTGCTTCC	
NtZIP4	F: AGAACAAGGGGCATGTCAAG	176
	R: CTGAAACACCCAATGCTATGC	
AtHMA4	F:TGCCAAGCTGGTTTGTTGTG	169
	R:CGTGGAACAATTCGTGCTCTC	
