# Supplementary material for: The ratio of Zn to Cd supply as a determinant of metal-homeostasis gene expression in tobacco and its modulation by overexpressing the metal exporter AtHMA4
Source: J Exp Bot. 2016 Oct 17;67(21):6201–14. doi: 10.1093/jxb/erw389 (PMC5100030; doi:10.1093/jxb/erw389)
Supplement: Supplementary Data [file supp_erw389_supplementary_protocol_S1.pdf]

Supplementary Protocol S1

This file details the protocol for the Suppression Subtractive Hybridization (SSH) used in this study by complementing the description of the basic steps with all details for each procedure.

Construction of SSH cDNA libraries

To identify genes with differential expression upon exposure to Cd (both up- and down-regulated) in transgenic plants relative to the wild-type, two SSH cDNA libraries were constructed. For SSH, double-stranded cDNA was obtained by using the SMART-PCR cDNA synthesis Kit (Clontech, compatible with the PCR-Select^{TM} cDNA Subtraction Kit, used in the later stage of the experiments) and Phusion Hot-Start II High-Fidelity DNA Polymerase with GC buffer (Thermo Scientific) from 800 ng of total RNA isolated from Cd-treated root samples from transgenic and wild-type tobacco (Fig. SP1). For the forward-subtracted cDNA library (T library), amplified cDNA (Fig. SP2-3) from transgenic tobacco was used as the "tester", whereas amplified cDNA from the wild-type as the "driver". The reverse-subtracted cDNA library (WT library) was made with wild-type (WT) cDNA as the "tester" and transgenic cDNA as the driver". PCR-selected cDNA subtraction was performed according to the instructions of the PCR-Select^{TM} cDNA subtraction Kit (Clontech). Amplified and purified cDNA (2 µg) was digested with the enzyme, RsaI (Fig. SP4). The reaction mixture was then purified (MinElute Reaction Cleanup Kit, Qiagen). Each cDNA from the tester samples was then separated into two portions, and adapter 1 and 2R were each added to one of the two parts. The fragment of the tac9 gene encoding *N. tabacum* actin was amplified with specific primers using adapter-ligated cDNA as the template in order to test ligation efficiency (as recommended by the manufacturer) (Fig. SP5).

In the first hybridization, the tester samples were hybridized with excess drivers at a tester:driver ratio of 1:30 at 68 °C for 9 h. In the second hybridization, 1 µl of the driver mixture (1 µl hybridization buffer, 1 µl driver, 2 µl water) was hybridized with the first hybridization solution at 68 °C overnight. PCR amplification was then performed using PCR Primer 1 (19 cycles), followed by nested PCR using primer 1 and primer 2R (12 cycles). The quality and efficiency of the consecutive reactions were monitored (Fig. SP6). Following SSH, PCR products from subtracted samples were inserted into pGEM-T easy vector (Promega, USA) and then transferred into chemically competent *Escherichia coli*

cells (JM109, $>10^8$ cfu/µl, Promega) to generate libraries. All transformed bacteria (the libraries) were plated on LB medium containing 100 µg/ml ampicillin 50 µg/ml 5-bromo-4-chloro-3-indolyl b D-galactopyranoside (X-Gal), and 0.5 mM isopropyl-β-D-thiogalactopyranoside (IPTG). The plates were kept at 37 °C overnight to obtain subtracted recombinant clones. Each single colony was then grown overnight in 2.5 ml of liquid LB medium in the presence of 100 µg/ml ampicillin, and further stored at –80 °C as glycerol stocks for PCR amplification of the cDNA inserts. For library T, 1704 colonies, and for library WT, 1824 colonies were stored.

PCR amplification of cDNA inserts

All recombinant clones were used for PCR-based amplification of inserts. Bacteria from glycerol stocks were recovered on LB plates with ampicillin. Next, using a sterile toothpick, a small portion of bacteria from one colony was suspended in 10 µl of water and heated at 98 °C for 5 min. Then 10 µl of 2x PCR stock (2x PCR buffer, 4 mM $MgCl_2$, 0.4 µM of each primer, 0.4 mM dNTPs, 0.5u Taq polymerase – Thermo Scientific) was added. The cDNA inserts were amplified with nested Primer 1 and 2R provided in the SSH construction kit. The following PCR amplifying conditions were used: 95 °C for 2 min; 30 cycles of 95 °C for 30 s, 68 °C for 30s, 72 °C for 90s; and a final extension at 72 °C for 7 min. PCR products were analyzed by 1.2% (w/v) agarose gel electrophoresis and photographed (Fig. SP7). The remaining part was used for Northern blot hybridization.

Reverse Northern hybridization of subtracted cDNA libraries

The differential screening of subtracted cDNA libraries was performed by reverse Northern blot analysis to identify genes differentially expressed in transgenic tobacco (as compared with the wild-type) upon exposure to Cd. All amplified fragments of cDNA from forward- and reverse-subtracted libraries were used for hybridization. PCR products were denatured at 98 °C for 5 min, cooled, centrifuged, and 1 µl of each amplified fragment was spotted on positively charged Hybon Nylon membranes (GE Healthcare). Two identical membranes containing cDNA inserts were prepared. The DNA was fixed to the membrane by heating the blots for 2 h at 80 °C in an oven.

Each subtracted library was hybridized with forward- and reverse-subtracted probes (Fig. SP8) according to the guidelines in the “PCR-Selected Differential Screening Kit” (Clontech). The probes were digoxygenin labelled by using the “PCR DIG Probe Synthesis Kit” (Roche). Six nanograms of subtracted DNA was used to probe synthesis in one tube.

The first 10 cycles of amplification were performed without labeled dNTP (20 µl of reaction mixture, duration of synthesis,1 min 30 s), the second 10 cycles were performed with labeled dNTP (10 µl of PCR mixture with labeled dNTP was added, duration of synthesis, 2 min 30 s). Six microliters of PCR product was loaded to the gel, 24 µl was used as a probe for hybridization. Hybridization and detection were performed using DIG Easy Hyb, DIG Wash and Block Buffer Set, Anti-Digoxigenen-AP, Fab fragments, and NBT/BCIP Stock Solution reagents (all from Roche, Germany) according to procedures described in the manufacturer's manual. The pre-hybridization solution for two blots contained: DIG Easy Hyb buffer, 20 ml, salmon sperm DNA, 100 µl (10 mg/ml), Nested 1 and Nested 2R primers, and oligomers complemented to primers, 25µl each (100 µM). The hybridization solution for two blots contained: DIG easy Hyb buffer, 7 ml, salmon sperm DNA, 70 µl (10 mg/ml), Nested 1 and Nested 2R and oligomers complemented to primers, 8.75 µl each (100 µM) and 24 µl denatured probe. The results from the two hybridizations (two membranes containing the same clones hybridized with forward- and reverse-subtracted probes) were compared for each clone (Fig. SP9). Clones showing differential expression were selected for sequencing.

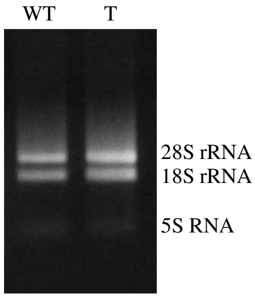

Figure SP1 – Electrophoresis of total RNA used for cDNA synthesis.
WT – RNA from wild type plants, T – RNA from *AtHMA4*-expressing plants.

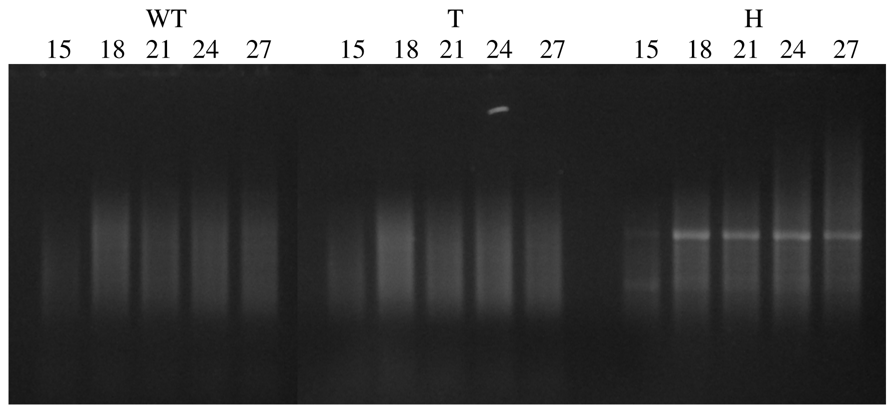

Figure SP2 Analysis for optimizing PCR parameters for cDNA amplification. WT - cDNA amplification of cDNA from WT plants, T – cDNA amplification of cDNA from *AtHMA4*-expressing plants, H – cDNA amplification of cDNA synthetized from control poly A (from human skeletal muscle). A range of PCR cycles were performed (15, 18, 21, 24, 27). 5 µl of PCR products collected after 15, 21, 24 and 27 cycle and 7,5 µl of PCR products collected after 17 cycle was loaded.

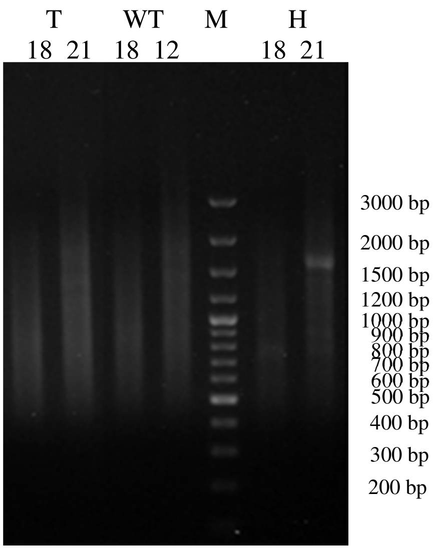

Figure SP3 cDNA amplification for SSH library construction. WT - cDNA amplification of cDNA from wild type plants, T – cDNA amplification of cDNA from *AtHMA4*-expressing plants, M – size marker, H – cDNA amplification of cDNA synthetized from control poly A (from human skeletal muscle). cDNA amplified at 21 cycles was chosen for future analysis. Results of parallel amplification at 18 cycles are shown also.

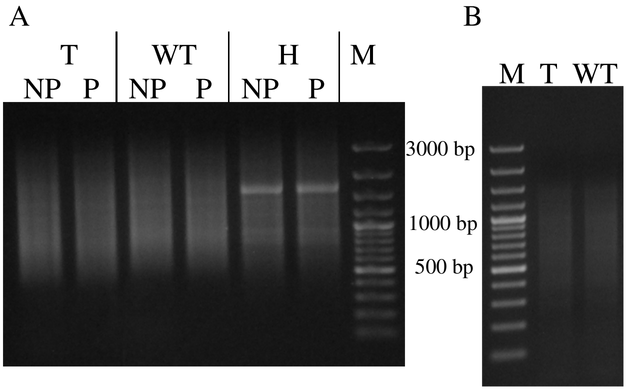

Figure SP4 *RsaI* digestion of amplified cDNA. (A) amplified cDNA, (B) – digested cDNA. T – amplified cDNA from *AtHMA4*-expressing plants, WT – amplified cDNA from wild type plants, H – cDNA amplification of cDNA synthetized from control poly A (human skeletal muscle), M – size marker, NP – cDNA after amplification and before purification, P – cDNA after amplification and purification.

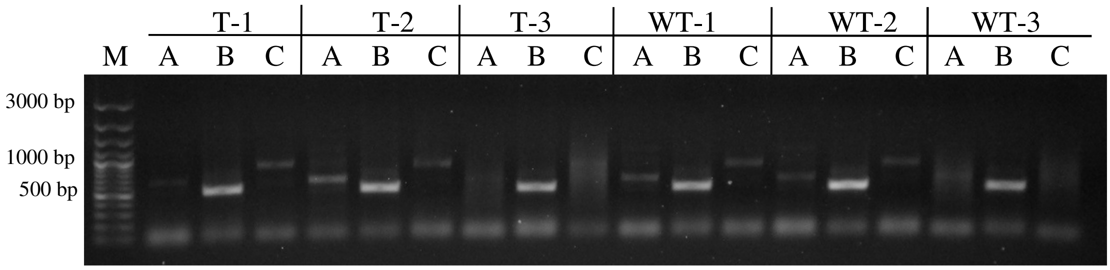

Figure SP5 Adapter ligation efficiency test. T – cDNA from *AtHMA4*-expressing plants as tester, WT – cDNA from wild type plants as tester, 1 – ligation with adapter1, 2 – ligation with adapter2, 3 – ligation with adapter1 and adapter2, A – PCR reaction using forward primer for internal gene and adapter primer , B – PCR reaction using forward and reverse primer for internal gene, C – PCR reaction using reverse primer for iternal gene and adapter primer

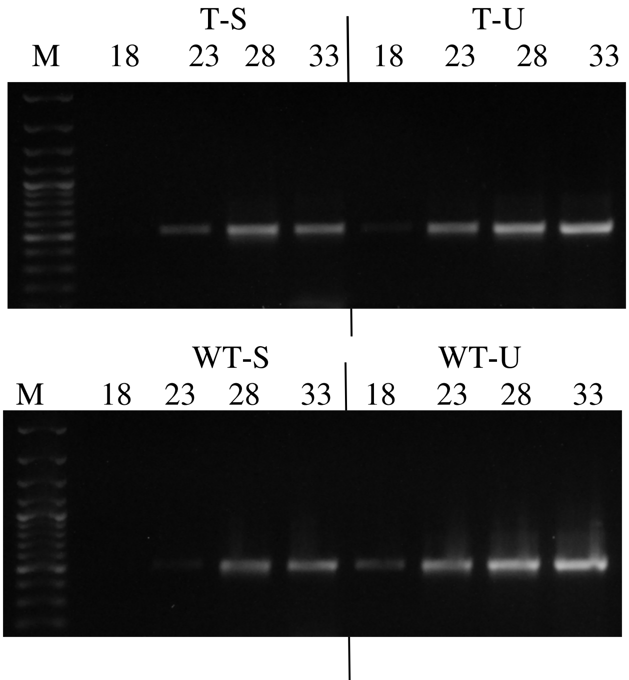

Figure SP6 – SSH subtraction efficiency detection. T-S – forward subtracted cDNA library, T-U – forward unsubtracted cDNA library, WT-S – reverse subtracted cDNA library, WT-U – reverse unsubtracted cDNA library, 18, 23, 28, 33 – number of PCR cycles.

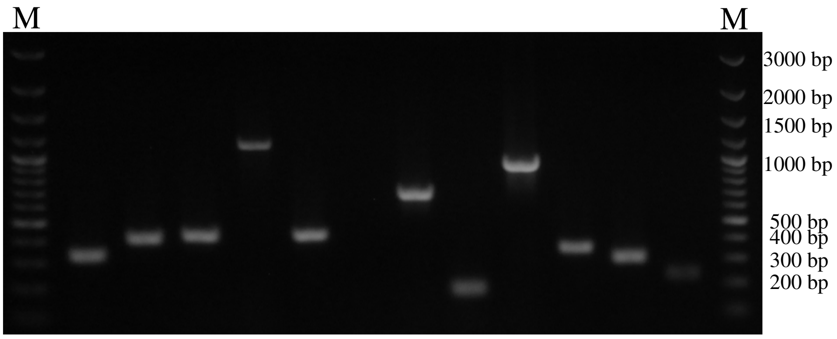

Figure SP7 Screening of the inserts in clones from SSH cDNA library – representative electrophoresis result. M – size marker

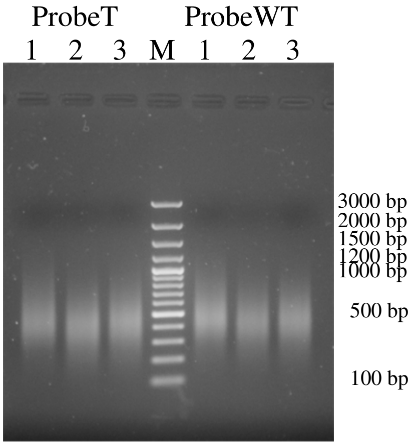

Figure SP8 – Electrophoresis of labeled forward- and reverse subtracted probe. ProbeT – forward subtracted probe**,** ProbeWT – reverse subtracted probe**,** 1,2,3 – independent synthesis reactions**,** M – size marker

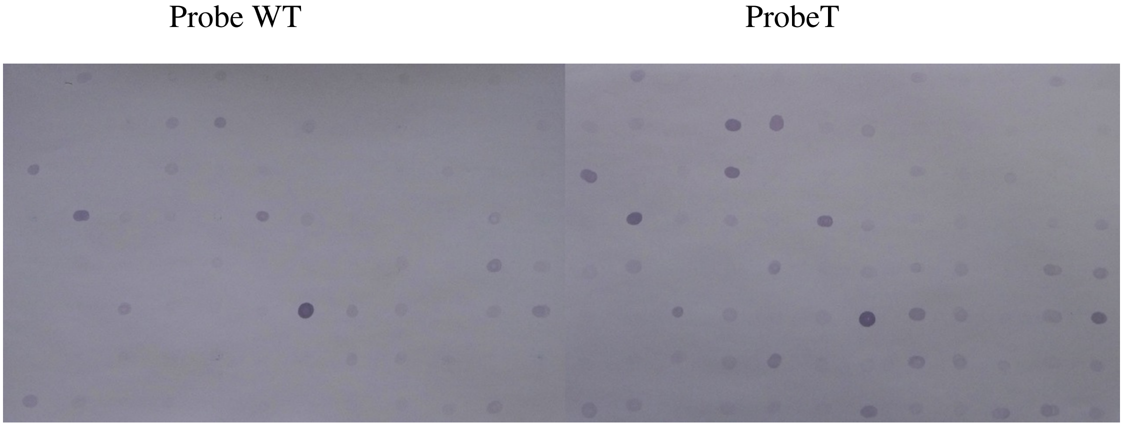

Figure SP9 Reverse Northern dot blot screening of SSH clones – example membranes with T library. ProbeWT – reverse subtracted probe, ProbeT – forward subtracted probe.
