# Supplementary material for: The ratio of Zn to Cd supply as a determinant of metal-homeostasis gene expression in tobacco and its modulation by overexpressing the metal exporter AtHMA4
Source: J Exp Bot. 2016 Oct 17;67(21):6201–14. doi: 10.1093/jxb/erw389 (PMC5100030; doi:10.1093/jxb/erw389)
Supplement: Supplementary Data [file supp_erw389_supplementary_dataset_S1.pdf]

Suplementary dataset S1: Alignment of sequences of metal homeostasis genes used for expression analysis with homologs from other plants

Alignment of: (i) nucleotide sequences of *N. tabacum* (v. Xanthi) selected by SSH (*ZIP2*, *ZIP4*, *IRT1*-like, *NAS*, *VTL*), with best hits from *N. tabacum*, *N. tomentosiformis*, *N. sylvestris*, *N. tabacum* EST and *Arabidopsis thaliana*; (ii) and deduced amino acid sequences,
Order – according to decreasing score.
In red – nucleotide (and predicted amino acid) sequence identified in *N. tabacum* (v. Xanthi) based on SSH analysis.

ZIP2

```
X-II-I1                    700 bp + poliA
GTACCCCTTTTCCTTCATGTTGGCTTCTGCAGGCTACCTTCTCACCATGTTTAGTGACTGCATTATCATGTTTGTGACAAAGGGTCA
TGAATCAAGTGAGGCCAAAGTTGAAGTAGAAGAAGGAAGGTCAGGTAATAATACTGAGGAGGGACATGGGGAAGCAAATCCTTTTCT
CAAGACAACTTCACTTGGGGACACAATACTTCTCATTCTTGCATTGTGTTTTCACTCTATTTTTGAAGGCATTGCTGTTGGAGTATC
AGCTACAAAGGGAGAAGCATGGAGAAATTTATGGACAATATCATTACACAAGATATTTGCAGCGATTGCAATGGGAATTGCACTTCT
AAGAATGATACCAAAGAGGCCATTTCTACTTACTTGTGCTTACTCTTTTGCCTTTGCTATTTCAAGCCCTATAGGTGTTGGAATAGG
AATAGCAATTGATGCTACAAGTGAAGGAAAAACAGCAGATCGGACTTATGCCATTTCCATGGCAATTGCTTGTGGAGTTTTCATATA
TGTGGCAATAAATCACCTTATATTAAAAGGTTTTAGGCCAAAGAACAAATGTTATTTTGACACTCAATTCTTCAAGTTTTTGCTGT
GTTTTCTGGAGTTGGGACTATTGCTATAGTCATGATATGGGACTAAGATCATTCGTTTTCTTTAATTTTACCGGCTTTGATTTGTTA
ACCCAAAAAAAAAAAAAAAAAAAAAA
```

Predicted amino acid sequence

```
X-II-I1 translated
YPFSFMLASAGYLLTMFSDCIIMFVTKGHESSEAKVEVEEGRSGNNTEEGHGEANPFLKTTSLGDTILLILALCFHSIFEGIAVGVS
ATKGEAWRNLWTISLHKIFAAIAMGIALLRMIPKRPFLLTCAYSFAFAISSPIGVGIGIAIDATSEGKTADRTYAISMAIACGVFIY
VAINHLILKGFRPKNKCYFDTQFFKFFAVFSGVGTIAIVMIWD
```

ALIGNMENT OF NUCLEOTIDE SEQUENCES

PREDICTED: Nicotiana sylvestris zinc transporter 2 (LOC104211888), mRNA
Sequence ID: ref|XM_009761025.1| **Length: 1237** Number of Matches: 1
Range 1: 393 to 1091 GenBank Graphics Next Match Previous Match

Alignment statistics for match #1

Score	Expect	Identities	Gaps	Strand
1280 bits(693)	0.0	**697/699(99%)**	0/699(0%)	Plus/Plus

```
Query  1    GTACCCCTTTTCCTTCATGTTGGCTTCTGCAGGCTACCTTCTCACCATGTTTAGTGACTG  60
            ||||||||||||||||||||||||||||||||||||||||||||||||||||||||||||
Sbjct  393  GTACCCCTTTTCCTTCATGTTGGCTTCTGCAGGCTACCTTCTCACCATGTTTAGTGACTG  452

Query  61   CATTATCATGTTTGTGACAAAGGGTCATGAATCAAGTGAGGCCAAAGTTGAAGTAGAAGA  120
            ||||||||||||||||||||||||||||||||||||||||||||||||||||||||||||
Sbjct  453  CATTATCATGTTTGTGACAAAGGGTCATGAATCAAGTGAGGCCAAAGTTGAAGTAGAAGA  512

Query  121  AGGAAGGTCAGGTAATAATACTGAGGAGGGACATGGGGAAGCAAATCCTTTTCTCAAGAC  180
            ||||||||||||||||||||||||||||||||||||||||||||||||||||||||||||
Sbjct  513  AGGAAGGTCAGGTAATAATACTGAGGAGGGACATGGGGAAGCAAATCCTTTTCTCAAGAC  572

Query  181  AACTTCACTTGGGGACACAATACTTCTCATTCTTGCATTGTGTTTTCACTCTATTTTTGA  240
            ||||||||||||||||||||||||||||||||||||||||||||||||||||||||||||
Sbjct  573  AACTTCACTTGGGGACACAATACTTCTCATTCTTGCATTGTGTTTTCACTCTATTTTTGA  632

Query  241  AGGCATTGCTGTTGGAGTATCAGCTACAAAGGGAGAAGCATGGAGAAATTTATGGACAAT  300
            ||||||||||||||||||||||||||||||||||||||||||||||||||||||||||||
Sbjct  633  AGGCATTGCTGTTGGAGTATCAGCTACAAAGGGAGAAGCATGGAGAAATTTATGGACAAT  692

Query  301  ATCATTACACAAGATATTTGCAGCGATTGCAATGGGAATTGCACTTCTAAGAATGATACC  360
            ||||||||||||||||||||||||| ||||||||||||||||||||||||||||||||||
Sbjct  693  ATCATTACACAAGATATTTGCAGCAATTGCAATGGGAATTGCACTTCTAAGAATGATACC  752

Query  361  AAAGAGGCCATTTCTACTTACTTGTGCTTACTCTTTTGCCTTTGCTATTTCAAGCCCTAT  420
```

```
              ||||||||||||||||||||||||||||||||||||||||||||||||||||||||||||
Sbjct  753    AAAGAGGCCATTTCTACTTACTTGTGCTTACTCTTTTGCCTTTGCTATTTCAAGCCCTAT  812

Query  421    AGGTGTTGGAATAGGAATAGCAATTGATGCTACAAGTGAAGGAAAAACAGCAGATCGGAC  480
              ||||||||||||||||||||||||||||||||||||||||||||||||||||||| ||||
Sbjct  813    AGGTGTTGGAATAGGAATAGCAATTGATGCTACAAGTGAAGGAAAAACAGCAGATTGGAC  872

Query  481    TTATGCCATTTCCATGGCAATTGCTTGTGGAGTTTTCATATATGTGGCAATAAATCACCT  540
              ||||||||||||||||||||||||||||||||||||||||||||||||||||||||||||
Sbjct  873    TTATGCCATTTCCATGGCAATTGCTTGTGGAGTTTTCATATATGTGGCAATAAATCACCT  932

Query  541    TATATTAAAAGGTTTAGGCCAAAGAACAAATGTTATTTTGACACTCAATTCTTCAAGTT  600
              ||||||||||||||||||||||||||||||||||||||||||||||||||||||||||||
Sbjct  933    TATATTAAAAGGTTTAGGCCAAAGAACAAATGTTATTTTGACACTCAATTCTTCAAGTT  992

Query  601    TTTTGCTGTGTTTTCTGGAGTTGGGACTATTGCTATAGTCATGATATGGGACTAAGATCA  660
              ||||||||||||||||||||||||||||||||||||||||||||||||||||||||||||
Sbjct  993    TTTTGCTGTGTTTTCTGGAGTTGGGACTATTGCTATAGTCATGATATGGGACTAAGATCA  1052

Query  661    TTCGTTTTCTTTAATTTTACCGGCTTTGATTTGTTAACC  699
              |||||||||||||||||||||||||||||||||||||||
Sbjct  1053   TTCGTTTTCTTTAATTTTACCGGCTTTGATTTGTTAACC  1091
```

PREDICTED: Nicotiana tomentosiformis zinc transporter 2 (LOC104101946), mRNA

Sequence ID: reflXM_009609507.1l Length: 1095 Number of Matches: 1

Range 1: 385 to 1078GenBankGraphics Next Match Previous Match

Alignment statistics for match #1

Score	Expect	Identities	Gaps	Strand
1138 bits(616)	0.0	**668/694(96%)**	0/694(0%)	Plus/Plus

```
Query  1      GTACCCCTTTTCCTTCATGTTGGCTTCTGCAGGCTACCTTCTCACCATGTTTAGTGACTG  60
              |||||||||||| |||||||||||||||||||||||||||||||||||||||||||||||
Sbjct  385    GTACCCCTTTTCTTTCATGTTGGCTTCTGCAGGCTACCTTCTCACCATGTTTAGTGACTG  444

Query  61     CATTATCATGTTTGTGACAAAGGGTCATGAATCAAGTGAGGCCAAAGTTGAAGTAGAAGA  120
              |||||||||||||||||||||||||||||||||||||||||||||||||||||||||||
Sbjct  445    CATTATCATGTTTGTGACAAAGGGTCATGAATCAAGTGAGGCCAAAGTTGAAGTAGAAGG  504

Query  121    AGGAAGGTCAGGTAATAATACTGAGGAGGGACATGGGGAAGCAAATCCTTTTCTCAAGAC  180
              ||||| ||||| ||||||||||||| |||||||||||||| |||||||||||||||||||
Sbjct  505    AGGAATGTCAGCTAATAATACTGAGAAGGGACATGGGGAAACAAATCCTTTTCTCAAGAC  564

Query  181    AACTTCACTTGGGGACACAATACTTCTCATTCTTGCATTGTGTTTTCACTCTATTTTTGA  240
              ||||||| ||||||||||||||||||||||||||||||||||||| ||||||||||||||
Sbjct  565    AACTTCATTTGGGGACACAATACTTCTCATTCTTGCATTGTGTTTCCACTCTATTTTTGA  624

Query  241    AGGCATTGCTGTTGGAGTATCAGCTACAAAGGGAGAAGCATGGAGAAATTTATGGACAAT  300
              ||||||||||||||| ||||||||||||||||||||||||||||||||| ||||||||||
Sbjct  625    AGGCATTGCTGTTGGGGTATCAGCTACAAAGGGAGAAGCATGGAGAAATCTATGGACAAT  684

Query  301    ATCATTACACAAGATATTTGCAGCGATTGCAATGGGAATTGCACTTCTAAGAATGATACC  360
               ||||| |||||||||||||||||  ||||||||||||||||||||||||| ||||||||
Sbjct  685    TTCATTGCACAAGATATTTGCAGCCGTTGCAATGGGAATTGCACTTCTAAGGATGATACC  744

Query  361    AAAGAGGCCATTTCTACTTACTTGTGCTTACTCTTTTGCCTTTGCTATTTCAAGCCCTAT  420
               ||||| |||||||||||||||||||||||||||||||||||||||||||||||||||||
Sbjct  745    TAAGAGACCATTTCTACTTACTTGTGCTTACTCTTTTGCCTTTGCTATTTCAAGCCCTAT  804

Query  421    AGGTGTTGGAATAGGAATAGCAATTGATGCTACAAGTGAAGGAAAAACAGCAGATCGGAC  480
              |||||||||||||||||| |||||||||||||||||||||||||||||||||||| ||||
Sbjct  805    AGGTGTTGGAATAGGAATTGCAATTGATGCTACAAGTGAAGGAAAAACAGCAGATTGGAC  864

Query  481    TTATGCCATTTCCATGGCAATTGCTTGTGGAGTTTTCATATATGTGGCAATAAATCACCT  540
              ||||||||||||||||| ||||||||||||||||||||||||||||||||||||||||||
Sbjct  865    TTATGCCATTTCCATGGGAATTGCTTGTGGAGTTTTCATATATGTGGCAATAAATCACCT  924

Query  541    TATATTAAAAGGTTTAGGCCAAAGAACAAATGTTATTTTGACACTCAATTCTTCAAGTT  600
              ||||| ||||||||||||||| ||||||||||||||||||||||||||||||||||||||
Sbjct  925    TATATCAAAAGGTTTAGGCCACAGAACAAATGTTATTTTGACACTCAATTCTTCAAGTT  984

Query  601    TTTTGCTGTGTTTTCTGGAGTTGGGACTATTGCTATAGTCATGATATGGGACTAAGATCA  660
              ||||||||||||||  ||||||||||||||||||||||||||||||||||||||||||||
Sbjct  985    TTTTGCTGTGTTTTTAGGAGTTGGGACTATTGCTATAGTCATGATATGGGACTAAGATCA  1044

Query  661    TTCGTTTTCTTTAATTTTACCGGCTTTGATTTGT  694
              |||||||||| |||||||||||||||| ||||||
Sbjct  1045   TTCGTTTTCTGTAATTTTACCGGCTTTAATTTGT  1078
```

Arabidopsis thaliana zinc transporter 2 mRNA, complete cds (AT5G59520)
Sequence ID: ref|NM_125344.2| **Length: 1292** Number of Matches: 1
Range 1: 632 to 1055GenBankGraphics Next Match Previous Match

Alignment statistics for match #1

Score	Expect	Identities	Gaps	Strand
174 bits(192)	9e-42	**293/424(69%)**	0/424(0%)	Plus/Plus

```
Query  189   TTGGGGACACAATACTTCTCATTCTTGCATTGTGTTTTCACTCTATTTTTGAAGGCATTG  248
             |||| ||||||    | || ||| ||||  | ||||||||||| || ||||| || || |
Sbjct  632   TTGGAGACACAGCTTTGCTGATTTTTGCTCTTTGTTTTCACTCCATCTTTGAGGGAATCG  691

Query  249   CTGTTGGAGTATCAGCTACAAAGGGAGAAGCATGGAGAAATTTATGGACAATATCATTAC  308
             |  ||||  | ||||  || ||  | || || ||||||||  ||||||||||||| || |
Sbjct  692   CCATTGGTCTCTCAGACACTAAAAGCGACGCTTGGAGAAACCTATGGACAATATCGTTGC  751

Query  309   ACAAGATATTTGCAGCGATTGCAATGGGAATTGCACTTCTAAGAATGATACCAAAGAGGC  368
             ||||| | ||||| ||  | ||||||||||| || ||||| |   | || || ||  | |
Sbjct  752   ACAAGGTCTTTGCGGCCGTAGCAATGGGAATAGCTCTTCTCAAGCTAATCCCTAAACGTC  811

Query  369   CATTTCTACTTACTTGTGCTTACTCTTTTGCCTTTGCTATTTCAAGCCCTATAGGTGTTG  428
             ||||  | || |||   |  ||||| || |||||||  || || || || |||||||| |
Sbjct  812   CATTCTTCCTCACTGTCGTCTACTCCTTCGCCTTTGGGATATCGAGTCCCATAGGTGTCG  871

Query  429   GAATAGGAATAGCAATTGATGCTACAAGTGAAGGAAAAACAGCAGATCGGACTTATGCCA  488
             | || || |||| |||  |||| || ||  |||||      |  ||  |||| || || |
Sbjct  872   GGATTGGCATAGGAATCAATGCCACTAGCCAAGGAGCTGGTGGTGACTGGACCTACGCGA  931

Query  489   TTTCCATGGCAATTGCTTGTGGAGTTTTCATATATGTGGCAATAAATCACCTTATATTAA  548
             | || ||||   |||| |||||||||||  | || || ||  | || || || || | ||
Sbjct  932   TCTCTATGGGCCTTGCGTGTGGAGTTTTTGTGTACGTTGCGGTTAACCATCTCATCTCAA  991

Query  549   AAGGTTTTAGGCCAAAGAACAAATGTTATTTTGACACTCAATTCTTCAAGTTTTTTGCTG  608
             |||| | || |||     |  ||||||| || ||||  | | ||| ||||||| |||| |
Sbjct  992   AAGGGTATAAGCCTCGTGAGGAATGTTACTTCGACAAGCCAATCTACAAGTTTATTGCCG  1051

Query  609   TGTT  612
             | ||
Sbjct  1052  TCTT  1055
```

ALIGNMENT OF AMINO ACID SEQUENCES

Alignment of predicted amino acid sequence X-II-I1 from *N. tabacum* v. Xanthi (nucleotide sequence identified by SSH) with selected proteins from *Nicotiana* species and from *A. thaliana*.

PREDICTED: zinc transporter 2 [Nicotiana sylvestris]
Sequence ID: ref|XP_009759327.1| **Length: 332** Number of Matches: 1
Range 1: 116 to 332GenPeptGraphics Next Match Previous Match

Alignment statistics for match #1

Score	Expect	Method	Identities	Positives	Gaps
440 bits(1131)	1e-155	Compositional matrix adjust.	**216/217(99%)**	**216/217(99%)**	0/217(0%)

```
Query  1    YPFSFMLASAGYLLTMFSDCIIMFVTKGHESSEAKVEVEEGRSGNNTEEGHGEANPFLKT  60
            YPFSFMLASAGYLLTMFSDCIIMFVTKGHESSEAKVEVEEGRSGNNTEEGHGEANPFLKT
Sbjct  116  YPFSFMLASAGYLLTMFSDCIIMFVTKGHESSEAKVEVEEGRSGNNTEEGHGEANPFLKT  175

Query  61   TSLGDTILLILALCFHSIFEGIAVGVSATKGEAWRNLWTISLHKIFAAIAMGIALLRMIP  120
            TSLGDTILLILALCFHSIFEGIAVGVSATKGEAWRNLWTISLHKIFAAIAMGIALLRMIP
Sbjct  176  TSLGDTILLILALCFHSIFEGIAVGVSATKGEAWRNLWTISLHKIFAAIAMGIALLRMIP  235

Query  121  KRPFLLTCAYSFAFAISSPIGVGIGIAIDATSEGKTADRTYAISMAIACGVFIYVAINHL  180
            KRPFLLTCAYSFAFAISSPIGVGIGIAIDATSEGKTAD TYAISMAIACGVFIYVAINHL
Sbjct  236  KRPFLLTCAYSFAFAISSPIGVGIGIAIDATSEGKTADWTYAISMAIACGVFIYVAINHL  295
```

```
Query  181   ILKGFRPKNKCYFDTQFFKFFAVFSGVGTIAIVMIWD  217
             ILKGFRPKNKCYFDTQFFKFFAVFSGVGTIAIVMIWD
Sbjct  296   ILKGFRPKNKCYFDTQFFKFFAVFSGVGTIAIVMIWD  332
```

PREDICTED: zinc transporter 2 [Nicotiana tomentosiformis]
Sequence ID: ref|XP_009607802.1| **Length: 343** Number of Matches: 1
Range 1: 127 to 343GenPeptGraphics Next Match Previous Match

Alignment statistics for match #1

Score	Expect	Method	Identities	Positives	Gaps
419 bits(1078)	2e-147	Compositional matrix adjust.	**205/217(94%)**	**208/217(95%)**	0/217(0%)

```
Query  1     YPFSFMLASAGYLLTMFSDCIIMFVTKGHESSEAKVEVEEGRSGNNTEEGHGEANPFLKT  60
             YPFSFMLASAGYLLTMFSDCIIMFVTKGHESSEAKVEVE G S NNTE+GHGE NPFLKT
Sbjct  127   YPFSFMLASAGYLLTMFSDCIIMFVTKGHESSEAKVEVEGGMSANNTEKGHGETNPFLKT  186

Query  61    TSLGDTILLILALCFHSIFEGIAVGVSATKGEAWRNLWTISLHKIFAAIAMGIALLRMIP  120
             TS GDTILLILALCFHSIFEGIAVGVSATKGEAWRNLWTISLHKIFAA+AMGIALLRMIP
Sbjct  187   TSFGDTILLILALCFHSIFEGIAVGVSATKGEAWRNLWTISLHKIFAAVAMGIALLRMIP  246

Query  121   KRPFLLTCAYSFAFAISSPIGVGIGIAIDATSEGKTADRTYAISMAIACGVFIYVAINHL  180
             KRPFLLTCAYSFAFAISSPIGVGIGIAIDATSEGKTAD TYAISM IACGVFIYVAINHL
Sbjct  247   KRPFLLTCAYSFAFAISSPIGVGIGIAIDATSEGKTADWTYAISMGIACGVFIYVAINHL  306

Query  181   ILKGFRPKNKCYFDTQFFKFFAVFSGVGTIAIVMIWD  217
             I KGFRP+NKCYFDTQFFKFFAVF GVGTIAIVMIWD
Sbjct  307   ISKGFRPQNKCYFDTQFFKFFAVFLGVGTIAIVMIWD  343
```

zinc transporter 2 [Arabidopsis thaliana] (AT5G59520 = ZIP2)
Sequence ID: ref|NP_200760.1| **Length: 353** Number of Matches: 1
Range 1: 127 to 353GenPeptGraphics Next Match Previous Match

Alignment statistics for match #1

Score	Expect	Method	Identities	Positives	Gaps
255 bits(651)	5e-83	Compositional matrix adjust.	**135/227(59%)**	**168/227(74%)**	10/227(4%)

```
Query  1     YPFSFMLASAGYLLTMFSDCIIMFVTKGHESSEAKVEVEEGRSGNNT---EEGHGE----  53
             YP++FMLA+AGY LTM +D  + FV  G  ++     V E R  ++    EEG  E
Sbjct  127   YPYAFMLAAAGYCLTMLADVAVAFVAAGSNNNHVGASVGESREDDDVAVKEEGRREIKSG  186

Query  54    ---ANPFLKTTSLGDTILLILALCFHSIFEGIAVGVSATKGEAWRNLWTISLHKIFAAIA  110
                +   ++T+  GDT LLI ALCFHSIFEGIA+G+S TK +AWRNLWTISLHK+FAA+A
Sbjct  187   VDVSQALIRTSGFGDTALLIFALCFHSIFEGIAIGLSDTKSDAWRNLWTISLHKVFAAVA  246

Query  111   MGIALLRMIPKRPFLLTCAYSFAFAISSPIGVGIGIAIDATSEGKTADRTYAISMAIACG  170
             MGIALL++IPKRPF LT  YSFAF ISSPIGVGIGI I+ATS+G   D TYAISM +ACG
Sbjct  247   MGIALLKLIPKRPFFLTVVYSFAFGISSPIGVGIGIGINATSQGAGGDWTYAISMGLACG  306

Query  171   VFIYVAINHLILKGFRPKNKCYFDTQFFKFFAVFSGVGTIAIVMIWD  217
             VF+YVA+NHLI KG++P+ +CYFD   +KF AVF GV  +++VMIWD
Sbjct  307   VFVYVAVNHLISKGYKPREECYFDKPIYKFIAVFLGVALLSVVMIWD  353
```

ZIP4

```
FL-XIII-K12       480 bp
GTACCGGTTGAACCAAAGGCAAGGAATGAGAAATTGTTTGGTGAAGAAGACGGTGGTGCAATACACATTGTTGGGATGCATGCACAT
GCAGCTCATCACAGACATAGCCATTCACAAGAACAAGGGGCATGTCAAGGGAACGTGGGGGAGCATTCCCATGGTCATTCGCACTCC
CATAGCTTTGGTGGTGGAGATGAGGAAGGTGGAGGGAGGCATGTTGTTGTTTCTCAGGTCTTGGAGCTGGGGATAGTATCACATTCT
CTCATAATAGGCATAGCATTGGGTGTTTCAGAAAGTCCGTGCACAATTAGACCCTTGCTTGTGGCCTTGTCGTTCCACCAGTTCTTC
GAAGGTTTTGCGTTAGGAGGCTGCATCTCACAGGCACAGTTCAATTCCCTCCGTTCCACTATAATGGCAACGTTTTTCGCCGTAACA
ACACCCTTGGGAATTGCTATAGGGATTCTAGCTTCTTCGTCGTAC

FL-XIII-K12 translated
VPVEPKARNEKLFGEEDGGAIHIVGMHAHAAHHRHSHSQEQGACQGNVGEHSHGHSHSHSFGGGDEEGGGRHVVVSQVLELGIVSHS
LIIGIALGVSESPCTIRPLLVALSFHQFFEGFALGGCISQAQFNSLRSTIMATFFAVTTPLGIAIGILASSSY
```

ALIGNMENT OF NUCLEOTIDE SEQUENCES

KR2B.108H16F.051229T7 KR2B Nicotiana tabacum cDNA clone KR2B.108H16, mRNA sequence.
Sequence ID: gb|EB445526.1| **Length: 871** Number of Matches: 1
Range 1: 16 to 495GenBankGraphics Next Match Previous Match

Alignment statistics for match #1

Score	Expect	Identities	Gaps	Strand
876 bits(474)	0.0	**478/480(99%)**	0/480(0%)	Plus/Plus

```
Query  1    GTACCGGTTGAACCAAAGGCAAGGAATGAGAAATTGTTTGGTGAAGAAGACGGTGGTGCA  60
            ||||||||||||||||||||||||||||||||||||||||||||||||||||||||||||
Sbjct  16   GTACCGGTTGAACCAAAGGCAAGGAATGAGAAATTGTTTGGTGAAGAAGACGGTGGTGCA  75

Query  61   ATACACATTGTTGGGATGCATGCACATGCAGCTCATCACAGACATAGCCATTCACAAGAA  120
            ||||||||||||||||||||||||||||||||||||||||||||||||||||||||||||
Sbjct  76   ATACACATTGTTGGGATGCATGCACATGCAGCTCATCACAGACATAGCCATTCACAAGAA  135

Query  121  CAAGGGGCATGTCAAGGGAACGTGGGGGAGCATTCCCATGGTCATTCGCACTCCCATAGC  180
            |||||||||||||||||||||||| |||||||| ||||||||||||||||||||||||||
Sbjct  136  CAAGGGGCATGTCAAGGGAACGTGAGGGAGCATCCCCATGGTCATTCGCACTCCCATAGC  195

Query  181  TTTGGTGGTGGAGATGAGGAAGGTGGAGGGAGGCATGTTGTTGTTTCTCAGGTCTTGGAG  240
            ||||||||||||||||||||||||||||||||||||||||||||||||||||||||||||
Sbjct  196  TTTGGTGGTGGAGATGAGGAAGGTGGAGGGAGGCATGTTGTTGTTTCTCAGGTCTTGGAG  255

Query  241  CTGGGGATAGTATCACATTCTCTCATAATAGGCATAGCATTGGGTGTTTCAGAAAGTCCG  300
            ||||||||||||||||||||||||||||||||||||||||||||||||||||||||||||
Sbjct  256  CTGGGGATAGTATCACATTCTCTCATAATAGGCATAGCATTGGGTGTTTCAGAAAGTCCG  315

Query  301  TGCACAATTAGACCCTTGCTTGTGGCCTTGTCGTTCCACCAGTTCTTCGAAGGTTTTGCG  360
            ||||||||||||||||||||||||||||||||||||||||||||||||||||||||||||
Sbjct  316  TGCACAATTAGACCCTTGCTTGTGGCCTTGTCGTTCCACCAGTTCTTCGAAGGTTTTGCG  375

Query  361  TTAGGAGGCTGCATCTCACAGGCACAGTTCAATTCCCTCCGTTCCACTATAATGGCAACG  420
            ||||||||||||||||||||||||||||||||||||||||||||||||||||||||||||
Sbjct  376  TTAGGAGGCTGCATCTCACAGGCACAGTTCAATTCCCTCCGTTCCACTATAATGGCAACG  435

Query  421  TTTTTCGCCGTAACAACACCCTTGGGAATTGCTATAGGGATTCTAGCTTCTTCGTCGTAC  480
            ||||||||||||||||||||||||||||||||||||||||||||||||||||||||||||
Sbjct  436  TTTTTCGCCGTAACAACACCCTTGGGAATTGCTATAGGGATTCTAGCTTCTTCGTCGTAC  495
```

PREDICTED: Nicotiana tomentosiformis zinc transporter 4, chloroplastic-like (LOC104108183), mRNA
Sequence ID: ref|XM_009617170.1| **Length: 1988** Number of Matches: 1
Range 1: 959 to 1438GenBankGraphics Next Match Previous Match

Alignment statistics for match #1

Score	Expect	Identities	Gaps	Strand
870 bits(471)	0.0	**477/480(99%)**	0/480(0%)	Plus/Plus

```
Query  1    GTACCGGTTGAACCAAAGGCAAGGAATGAGAAATTGTTTGGTGAAGAAGACGGTGGTGCA  60
            ||||| ||||||||||||| ||||||||||||||||||||||||||||||||||||||||
Sbjct  959  GTACCAGTTGAACCAAAGGTAAGGAATGAGAAATTGTTTGGTGAAGAAGACGGTGGTGCA  1018
```

```
Query  61    ATACACATTGTTGGGATGCATGCACATGCAGCTCATCACAGACATAGCCATTCACAAGAA  120
             ||||||||||||||||||||||||||||||||||||||||||||||||||||||||||||
Sbjct  1019  ATACACATTGTTGGGATGCATGCACATGCAGCTCATCACAGACATAGCCATTCACAAGAA  1078

Query  121   CAAGGGGCATGTCAAGGGAACGTGGGGGAGCATTCCCATGGTCATTCGCACTCCCATAGC  180
             |||||||||||||||||||||||| |||||||||||||||||||||||||||||||||||
Sbjct  1079  CAAGGGGCATGTCAAGGGAACGTGAGGGAGCATTCCCATGGTCATTCGCACTCCCATAGC  1138

Query  181   TTTGGTGGTGGAGATGAGGAAGGTGGAGGGAGGCATGTTGTTGTTTCTCAGGTCTTGGAG  240
             ||||||||||||||||||||||||||||||||||||||||||||||||||||||||||||
Sbjct  1139  TTTGGTGGTGGAGATGAGGAAGGTGGAGGGAGGCATGTTGTTGTTTCTCAGGTCTTGGAG  1198

Query  241   CTGGGGATAGTATCACATTCTCTCATAATAGGCATAGCATTGGGTGTTTCAGAAAGTCCG  300
             ||||||||||||||||||||||||||||||||||||||||||||||||||||||||||||
Sbjct  1199  CTGGGGATAGTATCACATTCTCTCATAATAGGCATAGCATTGGGTGTTTCAGAAAGTCCG  1258

Query  301   TGCACAATTAGACCCTTGCTTGTGGCCTTGTCGTTCCACCAGTTCTTCGAAGGTTTTGCG  360
             ||||||||||||||||||||||||||||||||||||||||||||||||||||||||||||
Sbjct  1259  TGCACAATTAGACCCTTGCTTGTGGCCTTGTCGTTCCACCAGTTCTTCGAAGGTTTTGCG  1318

Query  361   TTAGGAGGCTGCATCTCACAGGCACAGTTCAATTCCCTCCGTTCCACTATAATGGCAACG  420
             ||||||||||||||||||||||||||||||||||||||||||||||||||||||||||||
Sbjct  1319  TTAGGAGGCTGCATCTCACAGGCACAGTTCAATTCCCTCCGTTCCACTATAATGGCAACG  1378

Query  421   TTTTTCGCCGTAACAACACCCTTGGGAATTGCTATAGGGATTCTAGCTTCTTCGTCGTAC  480
             ||||||||||||||||||||||||||||||||||||||||||||||||||||||||||||
Sbjct  1379  TTTTTCGCCGTAACAACACCCTTGGGAATTGCTATAGGGATTCTAGCTTCTTCGTCGTAC  1438
```

PREDICTED: Nicotiana sylvestris zinc transporter 4, chloroplastic (LOC104214739), mRNA

Sequence ID: ref|XM_009764447.1| **Length: 1705** Number of Matches: 1

Range 1: 934 to 1413GenBankGraphics Next Match Previous Match

Alignment statistics for match #1

Score	Expect	Identities	Gaps	Strand
809 bits(438)	0.0	**466/480(97%)**	0/480(0%)	Plus/Plus

```
Query  1     GTACCGGTTGAACCAAAGGCAAGGAATGAGAAATTGTTTGGTGAAGAAGACGGTGGTGCA  60
             ||||| ||||||||||||||| |||||||||||||||||||||||||||| |||||||||
Sbjct  934   GTACCAGTTGAACCAAAGGCAGGGAATGAGAAATTGTTTGGTGAAGAAGATGGTGGTGCA  993

Query  61    ATACACATTGTTGGGATGCATGCACATGCAGCTCATCACAGACATAGCCATTCACAAGAA  120
             ||||||||||||||||||||||||||||||||||||||||||||||||||||||||||||
Sbjct  994   ATACACATTGTTGGGATGCATGCACATGCAGCTCATCACAGACATAGCCATTCACAAGAA  1053

Query  121   CAAGGGGCATGTCAAGGGAACGTGGGGGAGCATTCCCATGGTCATTCGCACTCCCATAGC  180
             |||||||||||||||||||||||| |||||||||||||||||||||||||||||||||||
Sbjct  1054  CAAGGGGCATGTCAAGGGAACGTGAGGGAGCATTCCCATGGTCATTCGCACTCCCATAGC  1113

Query  181   TTTGGTGGTGGAGATGAGGAAGGTGGAGGGAGGCATGTTGTTGTTTCTCAGGTCTTGGAG  240
             ||||||||||||||||||||||||||||||||||||||||||||||||||||||||||||
Sbjct  1114  TTTGGTGGTGGAGATGAGGAAGGTGGAGGGAGGCATGTTGTTGTTTCTCAGGTCTTGGAG  1173

Query  241   CTGGGGATAGTATCACATTCTCTCATAATAGGCATAGCATTGGGTGTTTCAGAAAGTCCG  300
             ||||| ||||||||||||||||||||||||||||||||||||||||||||||||||||| 
Sbjct  1174  CTGGGAATAGTATCACATTCTCTCATAATAGGCATAGCATTGGGTGTTTCAGAAAGTCCA  1233

Query  301   TGCACAATTAGACCCTTGCTTGTGGCCTTGTCGTTCCACCAGTTCTTCGAAGGTTTTGCG  360
             |||||||||||||||||||| |||||||| ||||||||||||||||||||||||||||||
Sbjct  1234  TGCACAATTAGACCCTTGCTCGTGGCCTTATCGTTCCACCAGTTCTTCGAAGGTTTTGCG  1293

Query  361   TTAGGAGGCTGCATCTCACAGGCACAGTTCAATTCCCTCCGTTCCACTATAATGGCAACG  420
             |||||||| |||||||| ||||||||||||||||||||||||||||||||||||||||||
Sbjct  1294  TTAGGAGGTTGCATCTCGCAGGCACAGTTCAATTCCCTCCGTTCCACTATAATGGCAACG  1353

Query  421   TTTTTCGCCGTAACAACACCCTTGGGAATTGCTATAGGGATTCTAGCTTCTTCGTCGTAC  480
             |||||||| ||||||||||||||||||||||||||||| |||||||||||||| || |||
Sbjct  1354  TTTTTCGCGGTAACAACACCCTTGGGAATTGCTATAGGAATTCTAGCTTCTTCATCTTAC  1413
```

Arabidopsis thaliana zinc transporter 4 precursor mRNA, complete cds

Sequence ID: ref|NM_100972.4| **Length: 1547** Number of Matches: 1

Range 1: 789 to 1137GenBankGraphics Next Match Previous Match

Alignment statistics for match #1

Score	Expect	Identities	Gaps	Strand
197 bits(218)	6e-49	**262/362(72%)**	14/362(3%)	Plus/Plus

```
Query  31    AAATTGTTTGGTGAAGAAGACGGTGGTGCAATACACATTGTTGGGATGCATGCACATGCA  90
             ||| |||||||||||||||||||||| |  || |||||||| || || | |||||||||
Sbjct  789   AAAGTGTTTGGTGAAGAAGACGGTGGCGGGATTCACATTGTCGGCATTCGTGCACATGCT  848

Query  91    GCTCATCACAGACATAGCCATTCACAAGAACAAGGGGCATGTCAAGGGA-ACGTGGGGGA  149
             ||||| || || ||||| || ||     || |        |||| || | | |||  |||
Sbjct  849   GCTCACCATAGGCATAGTCACTCT----AATA--------GTCATGGTACATGTGATGGA  896

Query  150   GCATTCCCATGGTCATTCGCACTCCCATAGCTTTGGTGGTGGAGATGAGGAAGGTGGAGG  209
              ||| | ||||| ||||| |||   ||||     ||    |  |||||  |||  |||||
Sbjct  897   -CATGCTCATGGACATTCACACGGACATATGCACGGGAATTCAGATGTTGAAAATGGAGC  955

Query  210   GAGGCATGTTGTTGTTTCTCAGGTCTTGGAGCTGGGGATAGTATCACATTCTCTCATAAT  269
              ||||||||||||||||||||| | |||||||| ||||| || || || ||  |||| ||
Sbjct  956   TAGGCATGTTGTTGTTTCTCAGATATTGGAGCTCGGGATTGTGTCGCACTCAATCATCAT  1015

Query  270   AGGCATAGCATTGGGTGTTTCAGAAAGTCCGTGCACAATTAGACCCTTGCTTGTGGCCTT  329
              ||  || |  |||| || ||| |   ||| ||||| || || ||  |  |||  ||  |
Sbjct  1016  CGGTTTATCCCTGGGGGTATCACAGTCTCCATGCACGATCAGGCCTCTTATTGCAGCTCT  1075

Query  330   GTCGTTCCACCAGTTCTTCGAAGGTTTTGCGTTAGGAGGCTGCATCTCACAGGCACAGTT  389
             ||| || ||||||||||| ||||| |||||| | |||||||||||||| |||||||||||
Sbjct  1076  GTCATTTCACCAGTTCTTTGAAGGATTTGCGCTCGGAGGCTGCATCTCTCAGGCACAGTT  1135

Query  390   CA  391
             ||
Sbjct  1136  CA  1137
```

ALIGNMENT OF AMINO ACID SEQUENCES

Alignment of predicted amino acid sequence FL-XIII-K12 from *N. tabacum* v. Xanthi (nucleotide sequence identified by SSH) with selected proteins from *Nicotiana* species and from *A. thaliana*.

PREDICTED: zinc transporter 4, chloroplastic-like [Nicotiana tomentosiformis]
Sequence ID: ref|XP_009615465.1| **Length: 412** Number of Matches: 1
Range 1: 185 to 344GenPeptGraphics Next Match Previous Match

Alignment statistics for match #1

Score	Expect	Method	Identities	Positives	Gaps
319 bits(818)	3e-108	Compositional matrix adjust.	**158/160(99%)**	158/160(98%)	0/160(0%)

```
Query  1    VPVEPKARNEKLFGEEDGGAIHIVGMHAHAAHHRHSHSQEQGACQGNVGEHSHGHSHSHS  60
            VPVEPK RNEKLFGEEDGGAIHIVGMHAHAAHHRHSHSQEQGACQGNV EHSHGHSHSHS
Sbjct  185  VPVEPKVRNEKLFGEEDGGAIHIVGMHAHAAHHRHSHSQEQGACQGNVREHSHGHSHSHS  244

Query  61   FGGGDEEGGGRHVVVSQVLELGIVSHSLIIGIALGVSESPCTIRPLLVALSFHQFFEGFA  120
            FGGGDEEGGGRHVVVSQVLELGIVSHSLIIGIALGVSESPCTIRPLLVALSFHQFFEGFA
Sbjct  245  FGGGDEEGGGRHVVVSQVLELGIVSHSLIIGIALGVSESPCTIRPLLVALSFHQFFEGFA  304

Query  121  LGGCISQAQFNSLRSTIMATFFAVTTPLGIAIGILASSSY  160
            LGGCISQAQFNSLRSTIMATFFAVTTPLGIAIGILASSSY
Sbjct  305  LGGCISQAQFNSLRSTIMATFFAVTTPLGIAIGILASSSY  344
```

PREDICTED: zinc transporter 4, chloroplastic [Nicotiana sylvestris]
Sequence ID: ref|XP_009762749.1| **Length: 412** Number of Matches: 1
Range 1: 185 to 344GenPeptGraphics Next Match Previous Match

Alignment statistics for match #1

Score	Expect	Method	Identities	Positives	Gaps
317 bits(813)	2e-107	Compositional matrix adjust.	**158/160(99%)**	**158/160(98%)**	0/160(0%)

```
Query  1    VPVEPKARNEKLFGEEDGGAIHIVGMHAHAAHHRHSHSQEQGACQGNVGEHSHGHSHSHS  60
            VPVEPKA NEKLFGEEDGGAIHIVGMHAHAAHHRHSHSQEQGACQGNV EHSHGHSHSHS
Sbjct  185  VPVEPKAGNEKLFGEEDGGAIHIVGMHAHAAHHRHSHSQEQGACQGNVREHSHGHSHSHS  244

Query  61   FGGGDEEGGGRHVVVSQVLELGIVSHSLIIGIALGVSESPCTIRPLLVALSFHQFFEGFA  120
            FGGGDEEGGGRHVVVSQVLELGIVSHSLIIGIALGVSESPCTIRPLLVALSFHQFFEGFA
Sbjct  245  FGGGDEEGGGRHVVVSQVLELGIVSHSLIIGIALGVSESPCTIRPLLVALSFHQFFEGFA  304

Query  121  LGGCISQAQFNSLRSTIMATFFAVTTPLGIAIGILASSSY  160
            LGGCISQAQFNSLRSTIMATFFAVTTPLGIAIGILASSSY
Sbjct  305  LGGCISQAQFNSLRSTIMATFFAVTTPLGIAIGILASSSY  344
```

zinc transporter 4 precursor [Arabidopsis thaliana] (AT1G10970 = ZIP4)

Sequence ID: ref|NP_172566.2| **Length: 408** Number of Matches: 1

Range 1: 184 to 340GenPeptGraphics Next Match Previous Match

Alignment statistics for match #1

Score	Expect	Method	Identities	Positives	Gaps
175 bits(444)	1e-52	Compositional matrix adjust.	**110/161(68%)**	127/161(78%)	5/161(3%)

```
Query  1    VPVE-PKARNEKLFGEEDGGAIHIVGMHAHAAHHRHSHSQEQGACQGNVGEHSHGHSHSH  59
            VPV   +  + K+FGEEDGG IHIVG+ AHAAHHRHSHS   G C G+   HSHGH H +
Sbjct  184  VPVVGERVTDNKVFGEEDGGGIHIVGIRAHAAHHRHSHSNSHGTCDGHAHGHSHGHMHGN  243

Query  60   SFGGGDEEGGGRHVVVSQVLELGIVSHSLIIGIALGVSESPCTIRPLLVALSFHQFFEGF  119
            S    D E G RHVVVSQ+LELGIVSHS+IIG++LGVS+SPCTIRPL+ ALSFHQFFEGF
Sbjct  244  S----DVENGARHVVVSQILELGIVSHSIIIGLSLGVSQSPCTIRPLIAALSFHQFFEGF  299

Query  120  ALGGCISQAQFNSLRSTIMATFFAVTTPLGIAIGILASSSY  160
            ALGGCISQAQF +  +TIMA FFA+TTPLGI IG   +SS+
Sbjct  300  ALGGCISQAQFRNKSATIMACFFALTTPLGIGIGTAVASSF  340
```

IRT1-like

FL-XIII-A9 insert at the nucleotide level is more closely related to AM839195 sequence (EST sequence from *N. tabacum*) than to AB263746 sequence (NtIRT1 gene) – 99% identity vs 96% identity respectively. Deduced amino acid sequence FL-XIII-A9 differs from NtIRT1 in one amino acid.

```
FL-XIII-A9         448 bp + poliA
GTACAAGTTCTTGAAGAAAGCAATAATGGCATTCTTCTTCGCAGTAACAACCCCATTTGGTATAGCACTTGGGATAGCACTTTCAAG
CACTTACGAGGAGAACAGTCCTCGGGCATTAATAACCGTTGGATTGCTCAATGCGTCATCTGCTGGTCTTCTTATTTACATGGCTTT
AGTAGATCTTCTTGCTGCAGATTTTATGGGTGACAAGTTACAAGGCAGCATTAAGCTACAGATCAAGTCTTACATGGCTGTTCTTCT
TGGTGCCGGTGGCATGTCTCTTATGGCCAAATGGGCCTAAGATTTGTTACCTTGATTGTTTTAGGAAATAAGTTTTTGTGTGAATCC
TCCCCCCTTTTTTTCTGTCCCTCCTTTCCTCCAATTTTAAAATTGTAATTTGGTATCCTTTTCATTTTCGTGACTCATTGTGAGTTT
CAACATATTGTGTAAAAAAAAAAAAAAAAAAAAAAAAAAAAAAA

FL-XIII-A9 translated
YKFLKKAIMAFFFAVTTPFGIALGIALSSTYEENSPRALITVGLLNASSAGLLIYMALVDLLAADFMGDKLQGSIKLQIKSYMAVLL
GAGGMSLMAKWA
```

ALIGNMENT OF NUCLEOTIDE SEQUENCES

(1) AM839195 DL, diurnal library Nicotiana tabacum cDNA clone nt005017008, mRNA sequence.

Sequence ID: emb|AM839195.1| **Length: 572** Number of Matches: 1

Range 1: 102 to 549GenBankGraphics Next Match Previous Match

Alignment statistics for match #1

Score	Expect	Identities	Gaps	Strand
803 bits(890)	0.0	**447/448(99%)**	0/448(0%)	Plus/Plus

```
Query  1    GTACAAGTTCTTGAAGAAAGCAATAATGGCATTCTTCTTCGCAGTAACAACCCCATTTGG  60
            ||||||||||||||||||||||||||||||||||||||||||||||||||||||||||||
Sbjct  102  GTACAAGTTCTTGAAGAAAGCAATAATGGCATTCTTCTTCGCAGTAACAACCCCATTTGG  161

Query  61   TATAGCACTTGGGATAGCACTTTCAAGCACTTACGAGGAGAACAGTCCTCGGGCATTAAT  120
            ||||||||||||||||||||||||||||||||||||||||||||||||||||||||||||
Sbjct  162  TATAGCACTTGGGATAGCACTTTCAAGCACTTACGAGGAGAACAGTCCTCGGGCATTAAT  221

Query  121  AACCGTTGGATTGCTCAATGCGTCATCTGCTGGTCTTCTTATTTACATGGCTTTAGTAGA  180
            ||||||||||||||||||||||||||||||||||||||||||||||||||||||||||||
Sbjct  222  AACCGTTGGATTGCTCAATGCGTCATCTGCTGGTCTTCTTATTTACATGGCTTTAGTAGA  281

Query  181  TCTTCTTGCTGCAGATTTTATGGGTGACAAGTTACAAGGCAGCATTAAGCTACAGATCAA  240
            ||||||||||||||||||||||||||||||||||||||||||||||||||||||||||||
Sbjct  282  TCTTCTTGCTGCAGATTTTATGGGTGACAAGTTACAAGGCAGCATTAAGCTACAGATCAA  341

Query  241  GTCTTACATGGCTGTTCTTCTTGGTGCCGGTGGCATGTCTCTTATGGCCAAATGGGCCTA  300
            ||||||||||||||||||||||||||||||||||||||||||||||||||||||||||||
Sbjct  342  GTCTTACATGGCTGTTCTTCTTGGTGCCGGTGGCATGTCTCTTATGGCCAAATGGGCCTA  401

Query  301  AGATTTGTTACCTTGATTGTTTTAGGAAATAAGTTTTTGTGTGAATCCTCCCCCCttttt  360
            ||||||||||||||||||||||||||||||||||||||||||||||||||||||||||||
Sbjct  402  AGATTTGTTACCTTGATTGTTTTAGGAAATAAGTTTTTGTGTGAATCCTCCCCCCTTTTT  461

Query  361  ttCTGTCCCTCCTTTCCTCCAATTTTAAAATTGTAATTTGGTATCCTTTTCATTTTCGTG  420
            ||||||||||||||||||||||||||||||||||||||||||||||||||||||||||||
Sbjct  462  TTCTGTCCCTCCTTTCCTCCAATTTTAAAATTGTAATTTGGTATCCTTTTCATTTTCGTG  521

Query  421  ACTCATTGTGAGTTTCAACATATTGTGT  448
            |||||| |||||||||||||||||||||
Sbjct  522  ACTCATCGTGAGTTTCAACATATTGTGT  549
```

(2) Nicotiana tabacum IRT1 mRNA for iron transporter protein IRT1, complete cds

Sequence ID: dbj|AB263746.1| **Length: 1326** Number of Matches: 1

Range 1: 838 to 1282GenBankGraphics Next Match Previous Match

Alignment statistics for match #1

Score	Expect	Identities	Gaps	Strand
717 bits(794)	0.0	**428/448(96%)**	3/448(0%)	Plus/Plus

```
Query  1     GTACAAGTTCTTGAAGAAAGCAATAATGGCATTCTTCTTCGCAGTAACAACCCCATTTGG  60
             |||||||||||||||||| |||||||||||||||||||||||| ||||||| ||||| ||
Sbjct  838   GTACAAGTTCTTGAAGAAGGCAATAATGGCATTCTTCTTCGCAATAACAACTCCATTCGG  897

Query  61    TATAGCACTTGGGATAGCACTTTCAAGCACTTACGAGGAGAACAGTCCTCGGGCATTAAT  120
             ||||||||||||||||||||||||||||||||||||||||||||| ||||||||||||||
Sbjct  898   TATAGCACTTGGGATAGCACTTTCAAGCACTTACGAGGAGAACAGCCCTCGGGCATTAAT  957

Query  121   AACCGTTGGATTGCTCAATGCGTCATCTGCTGGTCTTCTTATTTACATGGCTTTAGTAGA  180
             ||||||||||||||||||||||||||||||||| ||||| || || ||||||||||||||
Sbjct  958   AACCGTTGGATTGCTCAATGCGTCATCTGCTGGCCTTCTGATCTATATGGCTTTAGTAGA  1017

Query  181   TCTTCTTGCTGCAGATTTTATGGGTGACAAGTTACAAGGCAGCATTAAGCTACAGATCAA  240
             |||||||||||||||||||||||||||||||||||||||||| |||||||||||||||||
Sbjct  1018  TCTTCTTGCTGCAGATTTTATGGGTGACAAGTTACAAGGCAGTATTAAGCTACAGATCAA  1077

Query  241   GTCTTACATGGCTGTTCTTCTTGGTGCCGGTGGCATGTCTCTTATGGCCAAATGGGCCTA  300
             ||||||||||||||||||||||||||||||||||||||||||||||||||||||||||||
Sbjct  1078  GTCTTACATGGCTGTTCTTCTTGGTGCCGGTGGCATGTCTCTTATGGCCAAATGGGCCTA  1137

Query  301   AGATTTGTTACCTTGATTGTTTTAGGAAATAAGTTTTTGTGTGAATCCTCCCCCCttttt  360
             ||||| ||| |||||||||||||||||||||||||||||||||||||||||||   ||||
Sbjct  1138  AGATTCGTTTCCTTGATTGTTTTAGGAAATAAGTTTTTGTGTGAATCCTCCCC---TTTT  1194

Query  361   ttCTGTCCCTCCTTTCCTCCAATTTTAAAATTGTAATTTGGTATCCTTTTCATTTTCGTG  420
             |||  ||||||||||||||||||||||||||||||||||||||||||||||| |||||||
Sbjct  1195  TTCCCTCCCTCCTTTCCTCCAATTTTAAAATTGTAATTTGGTATCCTTTTCAATTTCGTG  1254

Query  421   ACTCATTGTGAGTTTCAACATATTGTGT  448
             |||||||||||| ||||| |||||||||
Sbjct  1255  ACTCATTGTGAGCTTCAAAATATTGTGT  1282
```

(3) PREDICTED: Nicotiana sylvestris probable zinc transporter 10 (LOC104247764), mRNA

Sequence ID: ref|XM_009803858.1| **Length: 1317** Number of Matches: 1

Range 1: 807 to 1251GenBankGraphics Next Match Previous Match

Alignment statistics for match #1

Score	Expect	Identities	Gaps	Strand
717 bits(794)	0.0	428/448(96%)	3/448(0%)	Plus/Plus

```
Query  1     GTACAAGTTCTTGAAGAAAGCAATAATGGCATTCTTCTTCGCAGTAACAACCCCATTTGG  60
             |||||||||||||||||| |||||||||||||||||||||||| ||||||| ||||| ||
Sbjct  807   GTACAAGTTCTTGAAGAAGGCAATAATGGCATTCTTCTTCGCAATAACAACTCCATTCGG  866

Query  61    TATAGCACTTGGGATAGCACTTTCAAGCACTTACGAGGAGAACAGTCCTCGGGCATTAAT  120
             ||||||||||||||||||||||||||||||||||||||||||||| ||||||||||||||
Sbjct  867   TATAGCACTTGGGATAGCACTTTCAAGCACTTACGAGGAGAACAGCCCTCGGGCATTAAT  926

Query  121   AACCGTTGGATTGCTCAATGCGTCATCTGCTGGTCTTCTTATTTACATGGCTTTAGTAGA  180
             ||||||||||||||||||||||||||||||||| ||||| || || ||||||||||||||
Sbjct  927   AACCGTTGGATTGCTCAATGCGTCATCTGCTGGCCTTCTGATCTATATGGCTTTAGTAGA  986

Query  181   TCTTCTTGCTGCAGATTTTATGGGTGACAAGTTACAAGGCAGCATTAAGCTACAGATCAA  240
             |||||||||||||||||||||||||||||||||||||||||| |||||||||||||||||
Sbjct  987   TCTTCTTGCTGCAGATTTTATGGGTGACAAGTTACAAGGCAGTATTAAGCTACAGATCAA  1046

Query  241   GTCTTACATGGCTGTTCTTCTTGGTGCCGGTGGCATGTCTCTTATGGCCAAATGGGCCTA  300
             ||||||||||||||||||||||||||||||||||||||||||||||||||||||||||||
Sbjct  1047  GTCTTACATGGCTGTTCTTCTTGGTGCCGGTGGCATGTCTCTTATGGCCAAATGGGCCTA  1106

Query  301   AGATTTGTTACCTTGATTGTTTTAGGAAATAAGTTTTTGTGTGAATCCTCCCCCCttttt  360
             ||||| ||| |||||||||||||||||||||||||||||||||||||||||||   ||||
Sbjct  1107  AGATTCGTTTCCTTGATTGTTTTAGGAAATAAGTTTTTGTGTGAATCCTCCCC---TTTT  1163

Query  361   ttCTGTCCCTCCTTTCCTCCAATTTTAAAATTGTAATTTGGTATCCTTTTCATTTTCGTG  420
             |||  ||||||||||||||||||||||||||||||||||||||||||||||| |||||||
Sbjct  1164  TTCCCTCCCTCCTTTCCTCCAATTTTAAAATTGTAATTTGGTATCCTTTTCAATTTCGTG  1223

Query  421   ACTCATTGTGAGTTTCAACATATTGTGT  448
             |||||||||||| ||||| |||||||||
Sbjct  1224  ACTCATTGTGAGCTTCAAAATATTGTGT  1251
```

Arabidopsis thaliana putative zinc transporter 10 mRNA, complete cds

Sequence ID: ref|NM_102864.2| **Length: 1095** Number of Matches: 1

Range 1: 820 to 1091GenBankGraphics Next Match Previous Match

Alignment statistics for match #1

Score	Expect	Identities	Gaps	Strand
170 bits(188)	7e-41	**201/272(74%)**	0/272(0%)	Plus/Plus

```
Query  26    ATGGCATTCTTCTTCGCAGTAACAACCCCATTTGGTATAGCACTTGGGATAGCACTTTCA  85
             ||||| ||||| || || || || || || |||||  | |  || ||||| ||||| || 
Sbjct  820   ATGGCTTTCTTTTTTGCGGTGACGACGCCTTTTGGAGTGGTTCTAGGGATGGCACTATCT  879

Query  86    AGCACTTACGAGGAGAACAGTCCTCGGGCATTAATAACCGTTGGATTGCTCAATGCGTCA  145
             |  || ||| | ||||| || |||    |  | ||||| ||||| |||||||| || || 
Sbjct  880   AAAACATACAAAGAGAATAGCCCTGAATCGCTTATAACAGTTGGGTTGCTCAACGCTTCC  939

Query  146   TCTGCTGGTCTTCTTATTTACATGGCTTTAGTAGATCTTCTTGCTGCAGATTTTATGGGT  205
             || || || || || || |||||||||||||| || ||||| ||||| ||||||||||||
Sbjct  940   TCGGCAGGACTACTCATCTACATGGCTTTAGTTGACCTTCTAGCTGCCGATTTTATGGGT  999

Query  206   GACAAGTTACAAGGCAGCATTAAGCTACAGATCAAGTCTTACATGGCTGTTCTTCTTGGT  265
              | ||  | ||| | ||||| ||||| ||  | ||||| ||    || ||| | ||||||
Sbjct  1000  CAAAAAATGCAAAGGAGCATCAAGCTTCAATTAAAGTCATATGCTGCCGTTTTGCTTGGT  1059

Query  266   GCCGGTGGCATGTCTCTTATGGCCAAATGGGC  297
             || |||||||||||  | |||||||| |||||
Sbjct  1060  GCTGGTGGCATGTCCGTCATGGCCAAGTGGGC  1091
```

Arabidopsis thaliana Fe(2+) transport protein 1 mRNA, complete cds ; IRT1

Sequence ID: ref|NM_118089.3|Length: 1377Number of Matches: 1

Range 1: 796 to 1085GenBankGraphics Next Match Previous Match

Alignment statistics for match #1

Score	Expect	Identities	Gaps	Strand
163 bits(180)	1e-38	**210/290(72%)**	0/290(0%)	Plus/Plus

```
Query  12    TGAAGAAAGCAATAATGGCATTCTTCTTCGCAGTAACAACCCCATTTGGTATAGCACTTG  71
             ||||||||    | ||||| ||||| ||||| |||||||| ||||| || |||||  | |
Sbjct  796   TGAAGAAATTTGTTATGGCGTTCTTTTTCGCGGTAACAACACCATTCGGAATAGCGTTAG  855

Query  72    GGATAGCACTTTCAAGCACTTACGAGGAGAACAGTCCTCGGGCATTAATAACCGTTGGAT  131
             |||| || || ||||    |||| | || || || ||    || || || || |||||| 
Sbjct  856   GGATCGCTCTATCAACTGTTTACCAAGATAATAGCCCAAAAGCTTTGATCACGGTTGGAC  915

Query  132   TGCTCAATGCGTCATCTGCTGGTCTTCTTATTTACATGGCTTTAGTAGATCTTCTTGCTG  191
             | || ||||| |  || |||||  | || |||||||||||  | || |||||||| ||||
Sbjct  916   TTCTAAATGCATGCTCCGCTGGATTGCTCATTTACATGGCACTCGTGGATCTTCTAGCTG  975

Query  192   CAGATTTTATGGGTGACAAGTTACAAGGCAGCATTAAGCTACAGATCAAGTCTTACATGG  251
             | || || |||||    ||| | ||||| ||||| ||  | ||| |||||| ||  || |
Sbjct  976   CGGAGTTCATGGGACCTAAGCTTCAAGGTAGCATCAAAATGCAGTTCAAGTGTTTAATCG  1035

Query  252   CTGTTCTTCTTGGTGCCGGTGGCATGTCTCTTATGGCCAAATGGGCCTAA  301
             | | |||||| ||   |||||| |||||  |||| ||||||||||| |||
Sbjct  1036  CGGCTCTTCTCGGGTGCGGTGGAATGTCGATTATCGCCAAATGGGCTTAA  1085
```

ALIGNMENT OF AMINO ACID SEQUENCES

Alignment of predicted amino acid sequence FL-XIII-A9 from *N. tabacum* v. Xanthi (nucleotide sequence identified by SSH) with selected proteins from *Nicotiana* species and from *A. thaliana*.

iron transporter protein IRT1 [Nicotiana tabacum]
Sequence ID: dbj|BAF48330.1|Length: 355Number of Matches: 1
Range 1: 257 to 355GenPeptGraphics Next Match Previous Match
Alignment statistics for match #1

Score	Expect	Method	Identities	Positives	Gaps
196 bits(498)	2e-63	Compositional matrix adjust.	98/99(99%)	99/99(100%)	0/99(0%)

```
Query  1    YKFLKKAIMAFFFAVTTPFGIALGIALSSTYEENSPRALITVGLLNASSAGLLIYMALVD  60
            YKFLKKAIMAFFFA+TTPFGIALGIALSSTYEENSPRALITVGLLNASSAGLLIYMALVD
Sbjct  257  YKFLKKAIMAFFFAITTPFGIALGIALSSTYEENSPRALITVGLLNASSAGLLIYMALVD  316

Query  61   LLAADFMGDKLQGSIKLQIKSYMAVLLGAGGMSLMAKWA  99
            LLAADFMGDKLQGSIKLQIKSYMAVLLGAGGMSLMAKWA
Sbjct  317  LLAADFMGDKLQGSIKLQIKSYMAVLLGAGGMSLMAKWA  355
```

PREDICTED: probable zinc transporter 10 [Nicotiana sylvestris]
Sequence ID: ref|XP_009772860.1| **Length: 360** Number of Matches: 1
Range 1: 262 to 360GenPeptGraphics Next Match Previous Match
Alignment statistics for match #1

Score	Expect	Method	Identities	Positives	Gaps
196 bits(499)	5e-62	Compositional matrix adjust.	**99/99(100%)**	**99/99(100%)**	0/99(0%)

```
Query  1    YKFLKKAIMAFFFAVTTPFGIALGIALSSTYEENSPRALITVGLLNASSAGLLIYMALVD  60
            YKFLKKAIMAFFFAVTTPFGIALGIALSSTYEENSPRALITVGLLNASSAGLLIYMALVD
Sbjct  262  YKFLKKAIMAFFFAVTTPFGIALGIALSSTYEENSPRALITVGLLNASSAGLLIYMALVD  321

Query  61   LLAADFMGDKLQGSIKLQIKSYMAVLLGAGGMSLMAKWA  99
            LLAADFMGDKLQGSIKLQIKSYMAVLLGAGGMSLMAKWA
Sbjct  322  LLAADFMGDKLQGSIKLQIKSYMAVLLGAGGMSLMAKWA  360
```

PREDICTED: probable zinc transporter 10 [Nicotiana tomentosiformis]
Sequence ID: ref|XP_009611861.1| **Length: 251** Number of Matches: 2
Range 1: 153 to 251GenPeptGraphics Next Match Previous Match
Alignment statistics for match #1

Score	Expect	Method	Identities	Positives	Gaps
194 bits(493)	2e-62	Compositional matrix adjust.	**96/99(97%)**	**98/99(98%)**	0/99(0%)

```
Query  1    YKFLKKAIMAFFFAVTTPFGIALGIALSSTYEENSPRALITVGLLNASSAGLLIYMALVD  60
            YKFLKKAIMAFFF +TTPFGIALGIALSSTYEENSPRALITVGLLNASSAGLLIYMALVD
Sbjct  153  YKFLKKAIMAFFFTITTPFGIALGIALSSTYEENSPRALITVGLLNASSAGLLIYMALVD  212

Query  61   LLAADFMGDKLQGSIKLQIKSYMAVLLGAGGMSLMAKWA  99
```

```
           LLAADFMGDKLQGS+KLQIKSYMAVLLGAGGMSLMAKWA
Sbjct  213  LLAADFMGDKLQGSVKLQIKSYMAVLLGAGGMSLMAKWA  251
```

PREDICTED: fe(2+) transport protein 1-like [Nicotiana tomentosiformis]
Sequence ID: ref|XP_009604609.1| **Length: 355** Number of Matches: 1
Range 1: 257 to 355GenPeptGraphics Next Match Previous Match

Alignment statistics for match #1

Score	Expect	Method	Identities	Positives	Gaps
183 bits(464)	8e-57	Compositional matrix adjust.	**90/99(91%)**	**97/99(97%)**	0/99(0%)

```
Query  1    YKFLKKAIMAFFFAVTTPFGIALGIALSSTYEENSPRALITVGLLNASSAGLLIYMALVD  60
           YK LKKA+MAFFF+VTTPFGIALGIAL+ TY+ENSPRALITVGLLNASSAGLLIYMALVD
Sbjct  257  YKMLKKAVMAFFFSVTTPFGIALGIALAKTYQENSPRALITVGLLNASSAGLLIYMALVD  316

Query  61   LLAADFMGDKLQGSIKLQIKSYMAVLLGAGGMSLMAKWA  99
           LLAADFMGDKLQGSIKLQIK+++AVLLGAGGMSLMAKWA
Sbjct  317  LLAADFMGDKLQGSIKLQIKAFIAVLLGAGGMSLMAKWA  355
```

putative zinc transporter 10 [Arabidopsis thaliana] (AT1G31260 = ZIP10)
Sequence ID: ref|NP_174411.2| **Length: 364** Number of Matches: 1
Range 1: 266 to 364GenPeptGraphics Next Match Previous Match

Alignment statistics for match #1

Score	Expect	Method	Identities	Positives	Gaps
167 bits(424)	5e-51	Compositional matrix adjust.	**82/99(83%)**	92/99(92%)	0/99(0%)

```
Query  1    YKFLKKAIMAFFFAVTTPFGIALGIALSSTYEENSPRALITVGLLNASSAGLLIYMALVD  60
           Y ++KKA+MAFFFAVTTPFG+ LG+ALS TY+ENSP +LITVGLLNASSAGLLIYMALVD
Sbjct  266  YGWVKKAVMAFFFAVTTPFGVVLGMALSKTYKENSPESLITVGLLNASSAGLLIYMALVD  325

Query  61   LLAADFMGDKLQGSIKLQIKSYMAVLLGAGGMSLMAKWA  99
           LLAADFMG K+Q SIKLQ+KSY AVLLGAGGMS+MAKWA
Sbjct  326  LLAADFMGQKMQRSIKLQLKSYAAVLLGAGGMSVMAKWA  364
```

Fe(2+) transport protein 1 [Arabidopsis thaliana] (AT4G19690 = IRT1)
Sequence ID: ref|NP_567590.3| **Length: 347** Number of Matches: 1
Range 1: 249 to 347GenPeptGraphics Next Match Previous Match

Alignment statistics for match #1

Score	Expect	Method	Identities	Positives	Gaps
158 bits(399)	2e-47	Compositional matrix adjust.	**77/99(78%)**	88/99(88%)	0/99(0%)

```
Query  1    YKFLKKAIMAFFFAVTTPFGIALGIALSSTYEENSPRALITVGLLNASSAGLLIYMALVD  60
           Y  +KK +MAFFFAVTTPFGIALGIALS+ Y++NSP+ALITVGLLNA SAGLLIYMALVD
Sbjct  249  YTNMKKFVMAFFFAVTTPFGIALGIALSTVYQDNSPKALITVGLLNACSAGLLIYMALVD  308

Query  61   LLAADFMGDKLQGSIKLQIKSYMAVLLGAGGMSLMAKWA  99
           LLAA+FMG KLQGSIK+Q K  +A LLG GGMS++AKWA
Sbjct  309  LLAAEFMGPKLQGSIKMQFKCLIAALLGCGGMSIIAKWA  347
```

Alignment of predicted protein sequence NtIRT1 (AB263746.1 → BAF48330.1) with *A. thaliana* proteins. Order – according to decreasing scores.

putative zinc transporter 10 [Arabidopsis thaliana]

locus_tag="AT1G31260"

Sequence ID: ref|NP_174411.2|Length: 364Number of Matches: 1

Range 1: 14 to 364GenPeptGraphics Next Match Previous Match

Alignment statistics for match #1

Score	Expect	Method	Identities	Positives	Gaps
473 bits(1216)	6e-166	Compositional matrix adjust.	**235/351(67%)**	289/351(82%)	6/351(1%)

```
Query  11   IFILISI-FTPRALSVV-EDCGAEEDNSCVNKSKAFSLKIIAIVSILITSMIGVCLPLVT  68
            +F+L+SI   P ALS   +DC ++ + SC++K+KA  LK+++I SILITS+IGVCLP
Sbjct  14   LFLLLSISHFPGALSQSNKDCQSKSNYSCIDKNKALDLKLLSIFSILITSLIGVCLPFFA  73

Query  69   RSIPALSPERSLFVIVKAFAAGIILATGFMHVLPDSFDMLSSSCLKENPWHKFPFTGFVA  128
            RSIPA  PE+S F+IVK+FA+GIIL+TGFMHVLPDSF+MLSS CL +NPWHKFPF GFVA
Sbjct  74   RSIPAFQPEKSHFLIVKSFASGIILSTGFMHVLPDSFEMLSSPCLNDNPWHKFPFAGFVA  133

Query  129  MLSAIFTLAIDSMATSLYSKKNKAGVIPESQS-QDGDQEMGAVNAGNHVHSH---HHHGS  184
            M+SA+FTL +DS+ TS+++K  +  +  +  S + DQE+G V    HVHSH   H+
Sbjct  134  MMSAVFTLMVDSITTSVFTKSGRKDLRADVASVETPDQEIGHVQVHGHVHSHTLPHNLHG  193

Query  185  FSTKDGVDGAKLLRYRVIAMVLELGIIVHSIVIGLSLGASNNTCTIKGLVAALCFHQMFE  244
             + K+     +LLRYR++A+VLELGI+V SIVIGLS+G +NNTCTIKGLVAALCFHQMFE
Sbjct  194  ENDKELGSYLQLLRYRILAIVLELGIVVQSIVIGLSVGDTNNTCTIKGLVAALCFHQMFE  253

Query  245  GMGLGGCILQAEYKFLKKAIMAFFFAITTPFGIALGIALSSTYEENSPRALITVGLLNAS  304
            GMGLGGCILQAEY ++KKA+MAFFFA+TTPFG+ LG+ALS TY+ENSP +LITVGLLNAS
Sbjct  254  GMGLGGCILQAEYGWVKKAVMAFFFAVTTPFGVVLGMALSKTYKENSPESLITVGLLNAS  313

Query  305  SAGLLIYMALVDLLAADFMGDKLQGSIKLQIKSYMAVLLGAGGMSLMAKWA  355
            SAGLLIYMALVDLLAADFMG K+Q SIKLQ+KSY AVLLGAGGMS+MAKWA
Sbjct  314  SAGLLIYMALVDLLAADFMGQKMQRSIKLQLKSYAAVLLGAGGMSVMAKWA  364
```

Fe(2+) transport protein 1 [Arabidopsis thaliana]

locus_tag="AT4G19690"

Sequence ID: ref|NP_567590.3|Length: 347Number of Matches: 1

Range 1: 14 to 347GenPeptGraphics Next Match Previous Match

Alignment statistics for match #1

Score	Expect	Method	Identities	Positives	Gaps
447 bits(1151)	2e-156	Compositional matrix adjust.	**226/348(65%)**	271/348(77%)	14/348(4%)

```
Query  8    IAIIFILISIFTPRALSVVEDCGAEEDNSCVNKSKAFSLKIIAIVSILITSMIGVCLPLV  67
            + +IF+  +I +P   +  E+CG+E  N CVNK+KA  LK+IAI  ILI SMIGV  PL
Sbjct  14   LVLIFVSFAI-SPATSTAPEECGSESANPCVNKAKALPLKVIAIFVILIASMIGVGAPLF  72

Query  68   TRSIPALSPERSLFVIVKAFAAGIILATGFMHVLPDSFDMLSSSCLKENPWHKFPFTGFV  127
            +R++  L P+ ++F I+K FA+GIIL TGFMHVLPDSF+MLSS CL+ENPWHKFPF+GF+
Sbjct  73   SRNVSFLQPDGNIFTIIKCFASGIILGTGFMHVLPDSFEMLSSICLEENPWHKFPFSGFL  132


Query  128  AMLSAIFTLAIDSMATSLYSKKNKAGVIPESQSQDGDQEMGAVNAGNHVHSHHHHGSFST  187
            AMLS + TLAIDSMATSLY+ KN  G++P                 N V
Sbjct  133  AMLSGLITLAIDSMATSLYTSKNAVGIMPHGHGH-------GHGPANDVTLPIK------  179

Query  188  KDGVDGAKLLRYRVIAMVLELGIIVHSIVIGLSLGASNNTCTIKGLVAALCFHQMFEGMG  247
            +D    A+LLRYRVIAMVLELGIIVHS+VIGLSLGA+++TCTIKGL+AALCFHQMFEGMG
Sbjct  180  EDDSSNAQLLRYRVIAMVLELGIIVHSVVIGLSLGATSDTCTIKGLIAALCFHQMFEGMG  239
```

```
Query  248  LGGCILQAEYKFLKKAIMAFFFAITTPFGIALGIALSSTYEENSPRALITVGLLNASSAG  307
            LGGCILQAEY  +KK +MAFFFA+TTPFGIALGIALS+ Y++NSP+ALITVGLLNA SAG
Sbjct  240  LGGCILQAEYTNMKKFVMAFFFAVTTPFGIALGIALSTVYQDNSPKALITVGLLNACSAG  299

Query  308  LLIYMALVDLLAADFMGDKLQGSIKLQIKSYMAVLLGAGGMSLMAKWA  355
            LLIYMALVDLLAA+FMG KLQGSIK+Q K  +A LLG GGMS++AKWA
Sbjct  300  LLIYMALVDLLAAEFMGPKLQGSIKMQFKCLIAALLGCGGMSIIAKWA  347
```

Alignment of predicted protein sequence NtIRT1-like with NtIRT1 (AB263746.1 → BAF48330.1) , AtZIP10 (NM_102864.2 → NP_174411.2) and AtIRT1 (NM_118089.3 → NP_567590.3).
Blue background indicates AtIRT1 residues which are different from AtZIP10.
Green background indicates AtIRT1 residues which are different from NtIRT1-like.
Red background indicates NtIRT1 residue which is different from NtIRT1-like.
Eight transmembrane domains (TM I – TM VIII) are boxed. Predicted cytoplasmic loops and predicted extracellular regions are marked “cytop” and “extracel”, respectively (according to Rogers et al., 2000, PNAS 97: 12356-12360). AtIRT1 sequence is longer at the N-terminal end (eight amino acid, written in small letters) than in Rogers et al. (2000)

```
AtZIP10     MTKSHVIFSASIALFLLLSISHFPGALSQSNKDCQSKSNYSCIDKNKALDLKLLSIFSIL
NtIRT1      MA----CYKHNIAIIFIL-ISIFTPRALSVVEDCGAEEDNSCVNKSKAFSLKIIAIVSIL
IRT1-like   ------------------------------------------------------------
AtIRT1      masnsallMKTIFLVLIFVSFAISPATSTAPEECGSESANPCVNKAKALPLKVIAIFVIL
                                  extracelular

AtZIP10     ITSLIGVCLPFFARSIPAFQPEKSHFLIVKSFASGIILSTGFMHVLPDSFEMLSSPCLND
NtIRT1      ITSMIGVCLPLVTRSIPALSPERSLFVIVKAFAAGIILATGFMHVLPDSFDMLSSSCLKE
IRT1-like   ------------------------------------------------------------
AtIRT1      IASMIGVGAPLFSRNVSFLQPDGNIFTIIKCFASGIILGTGFMHVLPDSFEMLSSICLEE
              TM I              cytop           TM II           extracel

AtZIP10     NPWHKFPFAGFVAMMSAVFTLMVDSITTSVFTKSGRKDLRADVASVETPDQEIGHVQVHG
NtIRT1      NPWHKFPFTGFVAMLSAIFTLAIDSMATSLYSKKNKAGVIPESQS-QDGDQEMGAVNAGN
IRT1-like   ------------------------------------------------------------
AtIRT1      NPWHKFPFSGFLAMLSGLITLAIDSMATSLYTSKNAVGIMPHGHG-HGHGPAN-------
                        TM III                        cytop

AtZIP10     HVHSHTLPHNLHGENDKELGSYLQLLRYRILAIVLELGIVVQSIVIGLSVGDTNNTCTIK
NtIRT1      HVHSHHHHGSF---STKDGVDGAKLLRYRVIAMVLELGIIVHSIVIGLSLGASNNTCTIK
IRT1-like   ------------------------------------------------------------
AtIRT1      ------DVTLP---IKEDDSSNAQLLRYRVIAMVLELGIIVHSVVIGLSLGATSDTCTIK
                                                  TM IV           extracel.

AtZIP10     GLVAALCFHQMFEGMGLGGCILQAEYGWVKKAVMAFFFAVTTPFGVVLGMALSKTYKENS
NtIRT1      GLVAALCFHQMFEGMGLGGCILQAEYKFLKKAIMAFFFAITTPFGIALGIALSSTYEENS
IRT1-like   -------------------------YKFLKKAIMAFFFAVTTPFGIALGIALSSTYEENS
AtIRT1      GLIAALCFHQMFEGMGLGGCILQAEYTNMKKFVMAFFFAVTTPFGIALGIALSTVYQDNS
                       TM V           cytop         TM VI             extracel.

AtZIP10     PESLITVGLLNASSAGLLIYMALVDLLAADFMGQKMQRSIKLQLKSYAAVLLGAGGMSVM
NtIRT1      PRALITVGLLNASSAGLLIYMALVDLLAADFMGDKLQGSIKLQIKSYMAVLLGAGGMSLM
IRT1-like   PRALITVGLLNASSAGLLIYMALVDLLAADFMGDKLQGSIKLQIKSYMAVLLGAGGMSLM
AtIRT1      PKALITVGLLNACSAGLLIYMALVDLLAAEFMGPKLQGSIKMQFKCLIAALLGCGGMSII
                    TM VII                   cytop           TM VIII

AtZIP10     AKWA
NtIRT1      AKWA
IRT1-like   AKWA
AtIRT1      AKWA
              extracel
```

NAS

FL-XI-G1 744 bp

GTACAACAAGTGTGTGAATTATATGAACAGATCTCGAGATTGGAGAACCTTAGCCCTTCCAAAGATGTCAACATACTGTTTACAAAG
CTTGTTCACACGTGCATGCCCCCTAATCCCATTGATGTTTCAAAACTCTGTCAAAAAATTCAAGAAATTAGGTCTCATCTCATCAAA
CTATGTGGTGAAGCTGAAGGTCTTTTAGAGAGTCACTATTCCAAGATTCTTGGCTCTTTTGAAAACCCCCTTCACCATCTTGACATT
TTCCCATATTTTGACAATTACATCAAACTCAGCTTGCTTGAGTTCAACATCCTTACCAAAAATACTACAAAACCCCCAAACAAAATA
GCATTTATTGGATCAGGCCCTCTCCCTCTCACTTCTCTTGTTTTGGCTACTAAACATCTTACAACTACATACTTTCACAACTATGAT
ATTAGTTCTGAGGCTAATTCCCTGGCATCTCGTCTCGTGGCATCCGATCCTGACTTGTCTGATCGGATGACTTTTCACACGACGGAT
GTCATGGATGTAACGTGTGCCCTGAAGGACTACGATGTAGTCTTCTTGGCCGCGTTGGTTGGTATGGATAAGGAAGAGAAAGTTAAG
TTTGTCGATCATCTGGCTAAGTATATGGCTCCAGGAGCAACCCTGATGCTCAGGAGTGCACATGGTGCGCGCGCTTTTCTATATCCT
GTTCTTGATCCTCGTGATCTCAGAGGATTCGAGGTGCTTTCGGTGTAC

FL-XI-G1 translated

VQQVCELYEQISRLENLSPSKDVNILFTKLVHTCMPPNPIDVSKLCQKIQEIRSHLIKLCGEAEGLLESHYSKILGSFENPLHHLDI
FPYFDNYIKLSLLEFNILTKNTTKPPNKIAFIGSGPLPLTSLVLATKHLTTTYFHNYDISSEANSLASRLVASDPDLSDRMTFHTTD
VMDVTCALKDYDVVFLAALVGMDKEEKVKFVDHLAKYMAPGATLMLRSAHGARAFLYPVLDPRDLRGFEVLSVY

ALIGNMENT OF NUCLEOTIDE SEQUENCES

PREDICTED: Nicotiana tomentosiformis nicotianamine synthase (LOC104104973), mRNA

Sequence ID: reflXM_009613194.1l **Length: 1390** Number of Matches: 1

Range 1: 162 to 905GenBankGraphics Next Match Previous Match

Alignment statistics for match #1

Score	Expect	Identities	Gaps	Strand
1375 bits(744)	0.0	**744/744(100%)**	0/744(0%)	Plus/Plus

```
Query  1    GTACAACAAGTGTGTGAATTATATGAACAGATCTCGAGATTGGAGAACCTTAGCCCTTCC  60
            ||||||||||||||||||||||||||||||||||||||||||||||||||||||||||||
Sbjct  162  GTACAACAAGTGTGTGAATTATATGAACAGATCTCGAGATTGGAGAACCTTAGCCCTTCC  221

Query  61   AAAGATGTCAACATACTGTTTACAAAGCTTGTTCACACGTGCATGCCCCCTAATCCCATT  120
            ||||||||||||||||||||||||||||||||||||||||||||||||||||||||||||
Sbjct  222  AAAGATGTCAACATACTGTTTACAAAGCTTGTTCACACGTGCATGCCCCCTAATCCCATT  281

Query  121  GATGTTTCAAAACTCTGTCAAAAAATTCAAGAAATTAGGTCTCATCTCATCAAACTATGT  180
            ||||||||||||||||||||||||||||||||||||||||||||||||||||||||||||
Sbjct  282  GATGTTTCAAAACTCTGTCAAAAAATTCAAGAAATTAGGTCTCATCTCATCAAACTATGT  341

Query  181  GGTGAAGCTGAAGGTCTTTTAGAGAGTCACTATTCCAAGATTCTTGGCTCTTTTGAAAAC  240
            ||||||||||||||||||||||||||||||||||||||||||||||||||||||||||||
Sbjct  342  GGTGAAGCTGAAGGTCTTTTAGAGAGTCACTATTCCAAGATTCTTGGCTCTTTTGAAAAC  401

Query  241  CCCCTTCACCATCTTGACATTTTCCCATATTTTGACAATTACATCAAACTCAGCTTGCTT  300
            ||||||||||||||||||||||||||||||||||||||||||||||||||||||||||||
Sbjct  402  CCCCTTCACCATCTTGACATTTTCCCATATTTTGACAATTACATCAAACTCAGCTTGCTT  461

Query  301  GAGTTCAACATCCTTACCAAAAATACTACAAAACCCCCAAACAAAATAGCATTTATTGGA  360
            ||||||||||||||||||||||||||||||||||||||||||||||||||||||||||||
Sbjct  462  GAGTTCAACATCCTTACCAAAAATACTACAAAACCCCCAAACAAAATAGCATTTATTGGA  521

Query  361  TCAGGCCCTCTCCCTCTCACTTCTCTTGTTTTGGCTACTAAACATCTTACAACTACATAC  420
            ||||||||||||||||||||||||||||||||||||||||||||||||||||||||||||
Sbjct  522  TCAGGCCCTCTCCCTCTCACTTCTCTTGTTTTGGCTACTAAACATCTTACAACTACATAC  581

Query  421  TTTCACAACTATGATATTAGTTCTGAGGCTAATTCCCTGGCATCTCGTCTCGTGGCATCC  480
            ||||||||||||||||||||||||||||||||||||||||||||||||||||||||||||
Sbjct  582  TTTCACAACTATGATATTAGTTCTGAGGCTAATTCCCTGGCATCTCGTCTCGTGGCATCC  641

Query  481  GATCCTGACTTGTCTGATCGGATGACTTTTCACACGACGGATGTCATGGATGTAACGTGT  540
            ||||||||||||||||||||||||||||||||||||||||||||||||||||||||||||
Sbjct  642  GATCCTGACTTGTCTGATCGGATGACTTTTCACACGACGGATGTCATGGATGTAACGTGT  701

Query  541  GCCCTGAAGGACTACGATGTAGTCTTCTTGGCCGCGTTGGTTGGTATGGATAAGGAAGAG  600
            ||||||||||||||||||||||||||||||||||||||||||||||||||||||||||||
Sbjct  702  GCCCTGAAGGACTACGATGTAGTCTTCTTGGCCGCGTTGGTTGGTATGGATAAGGAAGAG  761

Query  601  AAAGTTAAGTTTGTCGATCATCTGGCTAAGTATATGGCTCCAGGAGCAACCCTGATGCTC  660
            ||||||||||||||||||||||||||||||||||||||||||||||||||||||||||||
Sbjct  762  AAAGTTAAGTTTGTCGATCATCTGGCTAAGTATATGGCTCCAGGAGCAACCCTGATGCTC  821
```

```
Query  661   AGGAGTGCACATGGTGCGCGCGCTTTTCTATATCCTGTTCTTGATCCTCGTGATCTCAGA  720
             ||||||||||||||||||||||||||||||||||||||||||||||||||||||||||||
Sbjct  822   AGGAGTGCACATGGTGCGCGCGCTTTTCTATATCCTGTTCTTGATCCTCGTGATCTCAGA  881

Query  721   GGATTCGAGGTGCTTTCGGTGTAC  744
             ||||||||||||||||||||||||
Sbjct  882   GGATTCGAGGTGCTTTCGGTGTAC  905
```

Nicotiana tabacum NtNAS1 gene for nicotianamine synthase, partial cds

Sequence ID: dbj|AB097697.1| **Length: 530** Number of Matches: 1

Range 1: 1 to 524GenBankGraphics Next Match Previous Match

Alignment statistics for match #1

Score	Expect	Identities	Gaps	Strand
941 bits(509)	0.0	**519/524(99%)**	0/524(0%)	Plus/Plus

```
Query  154   ATTAGGTCTCATCTCATCAAACTATGTGGTGAAGCTGAAGGTCTTTTAGAGAGTCACTAT  213
             ||||||||||||||||||||||| ||||||||||||||||||||||||||||||||||||
Sbjct  1     ATTAGGTCTCATCTCATCAAACTTTGTGGTGAAGCTGAAGGTCTTTTAGAGAGTCACTAT  60

Query  214   TCCAAGATTCTTGGCTCTTTTGAAAACCCCCTTCACCATCTTGACATTTTCCCATATTTT  273
             ||||||||||||||||||||||||||||||||||||||||||||||||||||||||||||
Sbjct  61    TCCAAGATTCTTGGCTCTTTTGAAAACCCCCTTCACCATCTTGACATTTTCCCATATTTT  120

Query  274   GACAATTACATCAAACTCAGCTTGCTTGAGTTCAACATCCTTACCAAAAATACTACAAAA  333
             ||||||||||||||||||||||||||||||||||||||||||||||||||||||||||||
Sbjct  121   GACAATTACATCAAACTCAGCTTGCTTGAGTTCAACATCCTTACCAAAAATACTACAAAA  180

Query  334   CCCCCAAACAAAATAGCATTTATTGGATCAGGCCCTCTCCCTCTCACTTCTCTTGTTTTG  393
             ||||||||||||||||||||||||||||||||||||||||||||||||||||||||||||
Sbjct  181   CCCCCAAACAAAATAGCATTTATTGGATCAGGCCCTCTCCCTCTCACTTCTCTTGTTTTG  240

Query  394   GCTACTAAACATCTTACAACTACATACTTTCACAACTATGATATTAGTTCTGAGGCTAAT  453
             ||||||||||||||||||||||||||||||||||||||||||||||||||||||||||||
Sbjct  241   GCTACTAAACATCTTACAACTACATACTTTCACAACTATGATATTAGTTCTGAGGCTAAT  300

Query  454   TCCCTGGCATCTCGTCTCGTGGCATCCGATCCTGACTTGTCTGATCGGATGACTTTTCAC  513
             ||||||||||||||||||||||||||||||||||||||||||||||||||||||||||||
Sbjct  301   TCCCTGGCATCTCGTCTCGTGGCATCCGATCCTGACTTGTCTGATCGGATGACTTTTCAC  360

Query  514   ACGACGGATGTCATGGATGTAACGTGTGCCCTGAAGGACTACGATGTAGTCTTCTTGGCC  573
             ||||||||||||||||||||||||||||||||||||||||||||||||||||||||||||
Sbjct  361   ACGACGGATGTCATGGATGTAACGTGTGCCCTGAAGGACTACGATGTAGTCTTCTTGGCC  420

Query  574   GCGTTGGTTGGTATGGATAAGGAAGAGAAAGTTAAGTTTGTCGATCATCTGGCTAAGTAT  633
             ||||||||||||||||||||||||||||||||||||||||||||||||||||||||||||
Sbjct  421   GCGTTGGTTGGTATGGATAAGGAAGAGAAAGTTAAGTTTGTCGATCATCTGGCTAAGTAT  480

Query  634   ATGGCTCCAGGAGCAACCCTGATGCTCAGGAGTGCACATGGTGC  677
             |||||||||||||||||||||||||| || |  |||||||||||
Sbjct  481   ATGGCTCCAGGAGCAACCCTGATGCTTAGAATGGCACATGGTGC  524
```

PREDICTED: Nicotiana sylvestris nicotianamine synthase (LOC104237053), mRNA

Sequence ID: ref|XM_009791128.1| **Length: 1437** Number of Matches: 1

Range 1: 170 to 913GenBankGraphics Next Match Previous Match

Alignment statistics for match #1

Score	Expect	Identities	Gaps	Strand
1225 bits(663)	0.0	**717/744(96%)**	0/744(0%)	Plus/Plus

```
Query  1     GTACAACAAGTGTGTGAATTATATGAACAGATCTCGAGATTGGAGAACCTTAGCCCTTCC  60
             ||||||||||||||||||||||||||||||||||||||||||||||||||||||||||||
Sbjct  170   GTACAACAAGTGTGTGAATTATATGAACAGATCTCGAGATTGGAGAACCTTAGCCCTTCC  229

Query  61    AAAGATGTCAACATACTGTTTACAAAGCTTGTTCACACGTGCATGCCCCCTAATCCCATT  120
             |||||||||||||||||||| ||||||||||| |||||||||||||| |||||||| |||
Sbjct  230   AAAGATGTCAACATACTGTTCACAAAGCTTGTCCACACGTGCATGCCTCCTAATCCTATT  289
```

```
Query  121   GATGTTTCAAAACTCTGTCAAAAAATTCAAGAAATTAGGTCTCATCTCATCAAACTATGT  180
             ||||| ||||||||||||||||||||||||||||||||||||||||||||||||||||||
Sbjct  290   GATGTCTCAAAACTCTGTCAAAAAATTCAAGAAATTAGGTCTCATCTCATCAAACTATGT  349

Query  181   GGTGAAGCTGAAGGTCTTTTAGAGAGTCACTATTCCAAGATTCTTGGCTCTTTTGAAAAC  240
             ||||||||||||||||||||||||||||||||||||||||||||||||||||||||||| 
Sbjct  350   GGTGAAGCTGAAGGTCTTTTAGAGAGTCACTATTCCAAGATTCTTGGCTCTTTTGAAAAT  409

Query  241   CCCCTTCACCATCTTGACATTTTCCCATATTTTGACAATTACATCAAACTCAGCTTGCTT  300
             || |||||||||||||| ||||| |||||||||||||||||||||||||| || ||||||
Sbjct  410   CCTCTTCACCATCTTGATATTTTTCCATATTTTGACAATTACATCAAACTTAGTTTGCTT  469

Query  301   GAGTTCAACATCCTTACCAAAAATACTACAAAACCCCCAAACAAAATAGCATTTATTGGA  360
             |||||||| ||| | ||||| |||||||||||| ||||||||||||| ||||||||||||
Sbjct  470   GAGTTCAATATCTTGACCAAGAATACTACAAAAACCCCAAACAAAATTGCATTTATTGGA  529

Query  361   TCAGGCCCTCTCCCTCTCACTTCTCTTGTTTTGGCTACTAAACATCTTACAACTACATAC  420
             ||||||||||||||||||||||| ||||||||||||||||||||||||||| |||| |||
Sbjct  530   TCAGGCCCTCTCCCTCTCACTTCACTTGTTTTGGCTACTAAACATCTTACATCTACTTAC  589


Query  421   TTTCACAACTATGATATTAGTTCTGAGGCTAATTCCCTGGCATCTCGTCTCGTGGCATCC  480
             ||||||||||| |||||||||||||||||||||||| |||||||||||||||||||||||
Sbjct  590   TTTCACAACTACGATATTAGTTCTGAGGCTAATTCCTTGGCATCTCGTCTCGTGGCATCC  649

Query  481   GATCCTGACTTGTCTGATCGGATGACTTTTCACACGACGGATGTCATGGATGTAACGTGT  540
             ||||||||||||||||||||||||||||||||||||||||||||||||||||||||||||
Sbjct  650   GATCCTGACTTGTCTGATCGGATGACTTTTCACACGACGGATGTCATGGATGTAACGTGT  709

Query  541   GCCCTGAAGGACTACGATGTAGTCTTCTTGGCCGCGTTGGTTGGTATGGATAAGGAAGAG  600
             ||||||||||||||||||||||||||||||||||||||||||||||||||||||||||||
Sbjct  710   GCCCTGAAGGACTACGATGTAGTCTTCTTGGCCGCGTTGGTTGGTATGGATAAGGAAGAG  769

Query  601   AAAGTTAAGTTTGTCGATCATCTGGCTAAGTATATGGCTCCAGGAGCAACCCTGATGCTC  660
             |||||||||||||||||||||||||||||||||||||||||||| |||||||||||||||
Sbjct  770   AAAGTTAAGTTTGTCGATCATCTGGCTAAGTATATGGCTCCAGGGGCAACCCTGATGCTC  829

Query  661   AGGAGTGCACATGGTGCGCGCGCTTTTCTATATCCTGTTCTTGATCCTCGTGATCTCAGA  720
             ||||||||||||||||||||||||||||| |||||||||||||||||| |||||||  ||
Sbjct  830   AGGAGTGCACATGGTGCGCGCGCTTTTCTGTATCCTGTTCTTGATCCTTGTGATCTACGA  889

Query  721   GGATTCGAGGTGCTTTCGGTGTAC  744
             ||||||||||||||||||||||||
Sbjct  890   GGATTCGAGGTGCTTTCGGTGTAC  913
```

Nicotiana tabacum nicotianamine synthase mRNA, complete cds

Sequence ID: gb|KJ001136.1| **Length: 954** Number of Matches: 1

Range 1: 28 to 771GenBankGraphics Next Match Previous Match

Alignment statistics for match #1

Score	Expect	Identities	Gaps	Strand
1214 bits(657)	0.0	**715/744(96%)**	0/744(0%)	Plus/Plus

```
Query  1     GTACAACAAGTGTGTGAATTATATGAACAGATCTCGAGATTGGAGAACCTTAGCCCTTCC  60
             ||||||||||||||||||||||||||||||||||||||||||||||||||||||||||||
Sbjct  28    GTACAACAAGTGTGTGAATTATATGAACAGATCTCGAGATTGGAGAACCTTAGCCCTTCC  87

Query  61    AAAGATGTCAACATACTGTTTACAAAGCTTGTTCACACGTGCATGCCCCCTAATCCCATT  120
             ||||||||||||| | |||| ||||||||||| |||||||||||||| |||||||| |||
Sbjct  88    AAAGATGTCAACACATTGTTCACAAAGCTTGTCCACACGTGCATGCCTCCTAATCCTATT  147

Query  121   GATGTTTCAAAACTCTGTCAAAAAATTCAAGAAATTAGGTCTCATCTCATCAAACTATGT  180
             ||||| ||||||||||||||||||||||||||||||||||||||||||||||||||||||
Sbjct  148   GATGTCTCAAAACTCTGTCAAAAAATTCAAGAAATTAGGTCTCATCTCATCAAACTATGT  207

Query  181   GGTGAAGCTGAAGGTCTTTTAGAGAGTCACTATTCCAAGATTCTTGGCTCTTTTGAAAAC  240
             ||||||||||||||||||||||||||||||||||||||||||||||||||||||||||| 
Sbjct  208   GGTGAAGCTGAAGGTCTTTTAGAGAGTCACTATTCCAAGATTCTTGGCTCTTTTGAAAAT  267

Query  241   CCCCTTCACCATCTTGACATTTTCCCATATTTTGACAATTACATCAAACTCAGCTTGCTT  300
             || |||||||||||||| ||||| |||||||||||||||||||||||||| || ||||||
Sbjct  268   CCTCTTCACCATCTTGATATTTTTCCATATTTTGACAATTACATCAAACTTAGTTTGCTT  327

Query  301   GAGTTCAACATCCTTACCAAAAATACTACAAAACCCCCAAACAAAATAGCATTTATTGGA  360
             |||||||| ||| | ||||| |||||||||||| ||||||||||||| ||||||||||||
Sbjct  328   GAGTTCAATATCTTGACCAAGAATACTACAAAAACCCCAAACAAAATTGCATTTATTGGA  387

Query  361   TCAGGCCCTCTCCCTCTCACTTCTCTTGTTTTGGCTACTAAACATCTTACAACTACATAC  420
```

```
                 ||||||||||||||||||||||| ||||||||||||||||||||||||||| |||| |||
Sbjct  388   TCAGGCCCTCTCCCTCTCACTTCACTTGTTTTGGCTACTAAACATCTTACATCTACTTAC  447

Query  421   TTTCACAACTATGATATTAGTTCTGAGGCTAATTCCCTGGCATCTCGTCTCGTGGCATCC  480
             |||||||||||||||||||||||||||||||||||| |||||||||||||||||||||||
Sbjct  448   TTTCACAACTATGATATTAGTTCTGAGGCTAATTCCTTGGCATCTCGTCTCGTGGCATCC  507

Query  481   GATCCTGACTTGTCTGATCGGATGACTTTTCACACGACGGATGTCATGGATGTAACGTGT  540
             ||||||||||||||||||||||||||||||||||||||||||||||||||||||||||||
Sbjct  508   GATCCTGACTTGTCTGATCGGATGACTTTTCACACGACGGATGTCATGGATGTAACGTGT  567

Query  541   GCCCTGAAGGACTACGATGTAGTCTTCTTGGCCGCGTTGGTTGGTATGGATAAGGAAGAG  600
             ||||||||||||||||||||||||||||||||||||||||||||||||||||||||||||
Sbjct  568   GCCCTGAAGGACTACGATGTAGTCTTCTTGGCCGCGTTGGTTGGTATGGATAAGGAAGAG  627

Query  601   AAAGTTAAGTTTGTCGATCATCTGGCTAAGTATATGGCTCCAGGAGCAACCCTGATGCTC  660
             |||||||||||||||||||||||||||||||||||||||||||| |||||||||||||||
Sbjct  628   AAAGTTAAGTTTGTCGATCATCTGGCTAAGTATATGGCTCCAGGGGCAACCCTGATGCTC  687


Query  661   AGGAGTGCACATGGTGCGCGCGCTTTTCTATATCCTGTTCTTGATCCTCGTGATCTCAGA  720
             ||||||||||||| ||||||||||||||| |||||||||||||||||| |||||||  ||
Sbjct  688   AGGAGTGCACATGATGCGCGCGCTTTTCTGTATCCTGTTCTTGATCCTTGTGATCTACGA  747

Query  721   GGATTCGAGGTGCTTTCGGTGTAC  744
             ||||||||||||||||||||||||
Sbjct  748   GGATTCGAGGTGCTTTCGGTGTAC  771
```

Nicotiana tabacum NtNAS2 gene for nicotianamine synthase, partial cds

Sequence ID: dbj|AB097698.1| **Length: 530** Number of Matches: 1

Range 1: 1 to 524GenBankGraphics Next Match Previous Match

Alignment statistics for match #1

Score	Expect	Identities	Gaps	Strand
846 bits(458)	0.0	**502/524(96%)**	0/524(0%)	Plus/Plus

```
Query  154   ATTAGGTCTCATCTCATCAAACTATGTGGTGAAGCTGAAGGTCTTTTAGAGAGTCACTAT  213
             ||||||||||||||||||||||| ||||||||||||||||||||||||||||||||||||
Sbjct  1     ATTAGGTCTCATCTCATCAAACTTTGTGGTGAAGCTGAAGGTCTTTTAGAGAGTCACTAT  60

Query  214   TCCAAGATTCTTGGCTCTTTTGAAAACCCCCTTCACCATCTTGACATTTTCCCATATTTT  273
             |||||||||||||||||||||||||| || |||||||||||||| ||||| |||||||||
Sbjct  61    TCCAAGATTCTTGGCTCTTTTGAAAATCCTCTTCACCATCTTGATATTTTTCCATATTTT  120

Query  274   GACAATTACATCAAACTCAGCTTGCTTGAGTTCAACATCCTTACCAAAAATACTACAAAA  333
             ||||||||||||||||| || |||||||||||||| ||| | ||||| ||||||||||||
Sbjct  121   GACAATTACATCAAACTTAGTTTGCTTGAGTTCAATATCTTGACCAAGAATACTACAAAA  180

Query  334   CCCCCAAACAAAATAGCATTTATTGGATCAGGCCCTCTCCCTCTCACTTCTCTTGTTTTG  393
              ||||||||||||| ||||||||||||||||||||||||||||||||||| |||||||||
Sbjct  181   ACCCCAAACAAAATTGCATTTATTGGATCAGGCCCTCTCCCTCTCACTTCACTTGTTTTG  240

Query  394   GCTACTAAACATCTTACAACTACATACTTTCACAACTATGATATTAGTTCTGAGGCTAAT  453
             |||||||||||||||||| |||| ||||||||||||||||||||||||||||||||||||
Sbjct  241   GCTACTAAACATCTTACATCTACTTACTTTCACAACTATGATATTAGTTCTGAGGCTAAT  300

Query  454   TCCCTGGCATCTCGTCTCGTGGCATCCGATCCTGACTTGTCTGATCGGATGACTTTTCAC  513
             ||| ||||||||||||||||||||||||||||||||||||||||||||||||||||||||
Sbjct  301   TCCTTGGCATCTCGTCTCGTGGCATCCGATCCTGACTTGTCTGATCGGATGACTTTTCAC  360

Query  514   ACGACGGATGTCATGGATGTAACGTGTGCCCTGAAGGACTACGATGTAGTCTTCTTGGCC  573
             ||||||||||||||||||||||||||||||||||||||||||||||||||||||||||||
Sbjct  361   ACGACGGATGTCATGGATGTAACGTGTGCCCTGAAGGACTACGATGTAGTCTTCTTGGCC  420

Query  574   GCGTTGGTTGGTATGGATAAGGAAGAGAAAGTTAAGTTTGTCGATCATCTGGCTAAGTAT  633
             ||||||||||||||||||||||||||||||||||||||||||||||||||||||||||||
Sbjct  421   GCGTTGGTTGGTATGGATAAGGAAGAGAAAGTTAAGTTTGTCGATCATCTGGCTAAGTAT  480

Query  634   ATGGCTCCAGGAGCAACCCTGATGCTCAGGAGTGCACATGGTGC  677
             ||||||||||| |||||||||||||| || |  |||||||||||
Sbjct  481   ATGGCTCCAGGGGCAACCCTGATGCTTAGAATGGCACATGGTGC  524
```

Arabidopsis thaliana nicotianamine synthase 4 mRNA, complete cds

Sequence ID: ref|NM_104521.2| **Length: 1144** Number of Matches: 1

Related Information

Range 1: 110 to 858GenBankGraphics Next Match Previous Match

Alignment statistics for match #1

Score	Expect	Identities	Gaps	Strand
221 bits(244)	8e-56	**511/758(67%)**	28/758(3%)	Plus/Plus

```
Query  5    AACAAG-TGTGTGAATTATATGAACAGATCTCGAGATTGGAGAACCTTAGCCCTTCCAAA  63
            |||||| | || ||  | || ||| |||||||||   | |||| ||| |  ||||   ||
Sbjct  110  AACAAGATCTGCGATCTTTACGAAAAGATCTCGAAGCTTGAGACCCTAAAGCCTTGTGAA  169

Query  64   GATGTCAACATACTGTTTACAAAGCTTGTTCACACGTGCATGCCCCCTAATCC---CATT  120
            |||||| |||  || || |   |||| ||   ||| |||||| || || || ||   |||
Sbjct  170  GATGTCGACACTCTCTTCAAGCAGCTCGTGTCCACATGCATACCACCAAACCCTAACATC  229

Query  121  GATGTTTCAAAACTCTGTCAAAAAATTCAAGAAATTAGGTCTCATCTCATCAAACTATGT  180
            || ||  | ||  | | | |||| || ||||| || || ||  | ||||||||| | |||
Sbjct  230  GACGTCACCAAGATGTCTGAAAACATCCAAGAGATGAGATCAAACCTCATCAAAATCTGT  289

Query  181  GGTGAAGCTGAAGGTCTTTTAGAGAGTCACTATTC--CAAGATTCTTGGC-TCTTTTGAA  237
            ||||| |||||||||   ||||||  |||||  ||  |||   ||||  | |||||||||
Sbjct  290  GGTGAGGCTGAAGGTTACTTAGAGCATCACTTCTCTTCAA---TCTTAACCTCTTTTGAA  346

Query  238  ---AACCCCCTTCACCATCTTGACATTTTCCCATATTTTGACAATTACATCAAACTCAGC  294
               ||||||||||| ||| |  |  |||| || || |   |||| ||| ||||||| |||
Sbjct  347  GATAACCCCCTTCATCATTTGAATCTTTTTCCTTACTACAACAACTACCTCAAACTAAGC  406

Query  295  TTGCTTGAGTTCAACATCCTTACCAAAAATACTACAAAACCC-------CCAAACAAAAT  347
              ||| |||||  |  ||||  | || |  ||  | ||||         ||||  |   |
Sbjct  407  AAGCTCGAGTTTGATCTCCT--CGAACAGAAC--CTAAACGGTTTTGTTCCAAGGACTGT  462

Query  348  AGCATTTATTGGATCAGGCCCTCTCCCTCTCACTTCTCTTGTTTTGGCTACTAAACATCT  407
            ||| || ||||| || || |||||||||||| |||||  | ||| | ||  ||   |||||
Sbjct  463  AGCTTTCATTGGCTCTGGTCCTCTCCCTCTTACTTCCGTCGTTCTTGCCTCTTCCCATCT  522


Query  408  TACA-ACTACATACTTTCACAACTATGATATTAGTTCTGAGGCTAATTCCCTGGCATCTC  466
             | | |||  || |||||| |||| ||| ||     |    || |||    | ||| |||
Sbjct  523  CAAAGACTCGAT-CTTTCATAACTTTGACATCGACCCATCAGCGAATATGGTAGCAGCTC  581

Query  467  GTCTCGTGGCATCCGATCCTGACTTGTCTGATCGGATGACTTTTCACACGACGGATGTCA  526
            || | ||  | || ||||||||  | ||| | || |||  ||| || ||    ||| | |
Sbjct  582  GTTTGGTTTCGTCTGATCCTGATCTTTCTCAACGTATGTTTTTCCATACTGTTGATATAA  641

Query  527  TGGATGTAA-CGTGTGCCCTGAAGGACTACGATGTAGTCTTCTTGGCCGCGTTGGTTGGT  585
            ||||||||| || | | | |||||| || ||| || || |||||||| ||  | || ||
Sbjct  642  TGGATGTAACCGAGAG-CTTGAAGGGCTTCGACGTTGTGTTCTTGGCTGCTCTTGTAGGG  700

Query  586  ATGGATAAGGAAGAGAAAGTTAAGTTTGTCGATCATCTGGCTAAGTATATGGCTCCAGGA  645
            ||||||||  | ||||| |||||| | ||||| ||||| |  ||  | ||| |||| ||
Sbjct  701  ATGGATAAAAAGGAGAAGGTTAAGGTGGTCGAGCATCTTGAGAAACACATGTCTCCTGGT  760

Query  646  GCAACCCTGATGCTCAGGAGTGCACATGGTGCGCGCGCTTTTCTATATCCTGTTCTTGAT  705
            ||    || ||||| || || || |||||  |  | |||||||| |||||  |  ||||
Sbjct  761  GCTTTGCTCATGCTGAGAAGCGCTCATGGACCTAGAGCTTTTCTCTATCCAATCGTTGAG  820

Query  706  CCTCGTGATCTCAGAGGATTCGAGGTGCTTTCGGTGTA  743
            ||| ||||||||  ||| ||||| ||  | ||||| ||
Sbjct  821  CCTTGTGATCTCGAAGGTTTCGAAGTTTTATCGGTTTA  858
```

ALIGNMENT OF AMINO ACID SEQUENCES

Alignment of predicted protein sequence FL-XI-G1 (in red) with NAS from *N.tomentosiformis* – NtomNAS (XM_009613194.1 → XP_009611489.1), NAS from *N.sylvestris* – NsylNAS (XM_009791128.1 → XP_009789430.1), NAS from *N.tabacum* – NtNAS1 (AB097697.1 → BAC77350.1), NtNAS2 (AB097698.1 → BAF76727.1) , NtNAS (KJ001136.1 → AHM22926.1), AtNAS1 (NM_120577.3 → NP_196114.1), AtNAS2 (NM_124990.1 → NP_200419.1) , AtNAS3 (NM_100794.3 → NP_172395.1) , AtNAS4 (NM_104521.2 → NP_176038.1)

Blue background indicates residues in tobacco NAS proteins which are different from FL-XI-G1.

Green background indicates residues in *A. thaliana* NAS proteins which are different from FL-XI-G1

```
FL-XI-G1     ---------VQQVCELYEQISRLENLSPSKDVNILFTKLVHTCMPPNP-IDVSKLC-QKI
NtomNAS      MVCPNSNPVVQQVCELYEQISRLENLSPSKDVNILFTKLVHTCMPPNP-IDVSKLC-QKI
NtNAS1       ------------------------------------------------------------
NtNAS        MVCPNSNPVVQQVCELYEQISRLENLSPSKDVNTLFTKLVHTCMPPNP-IDVSKLC-QKI
NsylNAS      MVCPNSNPVVQQVCELYEQISRLENLSPSKDVNILFTKLVHTCMPPNP-IDVSKLC-QKI
NtNAS2       ------------------------------------------------------------
AtNAS1       M-ACQNNLVVKQIIDLYDQISKLKSLKPSKNVDTLFGQLVSTCLPTDTNIDVTNMC-EEV
AtNAS2       M-ACENNLVVKQIMDLYNQISNLESLKPSKNVDTLFRQLVSTCLPTDTNIDVTEIHDEKV
AtNAS3       M-GCQDEQLVQTICDLYEKISKLESLKPSEDVNILFKQLVSTCIPPNPNIDVTKMC-DRV
AtNAS4       MGYCQDDQLVNKICDLYEKISKLETLKPCEDVDTLFKQLVSTCIPPNPNIDVTKMS-ENI


FL-XI-G1     QEIRSHLIKLCGEAEGLLESHYSKILGSFE---NPLHHLDIFPYFDNYIKLSLLEFNILT
NtomNAS      QEIRSHLIKLCGEAEGLLESHYSKILGSFE---NPLHHLDIFPYFDNYIKLSLLEFNILT
NtNAS1       --IRSHLIKLCGEAEGLLESHYSKILGSFE---NPLHHLDIFPYFDNYIKLSLLEFNILT
NtNAS        QEIRSHLIKLCGEAEGLLESHYSKILGSFE---NPLHHLDIFPYFDNYIKLSLLEFNILT
NsylNAS      QEIRSHLIKLCGEAEGLLESHYSKILGSFE---NPLHHLDIFPYFDNYIKLSLLEFNILT
NtNAS2       --IRSHLIKLCGEAEGLLESHYSKILGSFE---NPLHHLDIFPYFDNYIKLSLLEFNILT
AtNAS1       KDMRANLIKLCGEAEGYLEQHFSTILGSLQEDQNPLDHLHIFPYYSNYLKLGKLEFDLLS
AtNAS2       KDMRSHLIKLCGEAEGYLEQHFSAILGSFE--DNPLNHLHIFPYYNNYLKLGKLEFDLLS
AtNAS3       QEIRLNLIKICGLAEGHLENHFSSILTSYQ--DNPLHHLNIFPYYNNYLKLGKLEFDLLE
AtNAS4       QEMRSNLIKICGEAEGYLEHHFSSILTSFE--DNPLHHLNLFPYYNNYLKLSKLEFDLLE


FL-XI-G1     KNT-TKPPNKIAFIGSGPLPLTSLVLATKHLTTTYFHNYDISSEANSLASRLVASDPDLS
NtomNAS      KNT-TKPPNKIAFIGSGPLPLTSLVLATKHLTTTYFHNYDISSEANSLASRLVASDPDLS
NtNAS1       KNT-TKPPNKIAFIGSGPLPLTSLVLATKHLTTTYFHNYDISSEANSLASRLVASDPDLS
NtNAS        KNT-TKTPNKIAFIGSGPLPLTSLVLATKHLTSTYFHNYDISSEANSLASRLVASDPDLS
NsylNAS      KNT-TKTPNKIAFIGSGPLPLTSLVLATKHLTSTYFHNYDISSEANSLASRLVASDPDLS
NtNAS2       KNT-TKTPNKIAFIGSGPLPLTSLVLATKHLTSTYFHNYDISSEANSLASRLVASDPDLS
AtNAS1       QHS-SHVPTKIAFVGSGPMPLTSIVLAKFHLPNTTFHNFDIDSHANTLASNLVSRDPDLS
AtNAS2       QHT-THVPTKVAFIGSGPMPLTSIVLAKFHLPNTTFHNFDIDSHANTLASNLVSRDSDLS
AtNAS3       QNLNGFVPKSVAFIGSGPLPLTSIVLASFHLKDTIFHNFDIDPSANSLASLLVSSDPDIS
AtNAS4       QNLNGFVPRTVAFIGSGPLPLTSVVLASSHLKDSIFHNFDIDPSANMVAARLVSSDPDLS


FL-XI-G1     DRMTFHTTDVMDVTCALKDYDVVFLAALVGMDKEEKVKFVDHLAKYMAPGATLMLRSAHG
NtomNAS      DRMTFHTTDVMDVTCALKDYDVVFLAALVGMDKEEKVKFVDHLAKYMAPGATLMLRSAHG
NtNAS1       DRMTFHTTDVMDVTCALKDYDVVFLAALVGMDKEEKVKFVDHLAKYMAPGATLMLRMAHG
NtNAS        DRMTFHTTDVMDVTCALKDYDVVFLAALVGMDKEEKVKFVDHLAKYMAPGATLMLRSAHD
NsylNAS      DRMTFHTTDVMDVTCALKDYDVVFLAALVGMDKEEKVKFVDHLAKYMAPGATLMLRSAHG
NtNAS2       DRMTFHTTDVMDVTCALKDYDVVFLAALVGMDKEEKVKFVDHLAKYMAPGATLMLRMAHG
AtNAS1       KRMIFHTTDVLNATEGLDQYDVVFLAALVGMDKESKVKAIEHLEKHMAPGAVLMLRSAHA
AtNAS2       KRMIFHTTDVLNAKEGLDQYDVVFLAALVGMDKESKVKAIEHLEKHMAPGAVVMLRSAHG
AtNAS3       QRMFFHTVDIMDVTESLKSFDVVFLAALVGMNKEEKVKVIEHLQKHMAPGAVLMLRSAHG
AtNAS4       QRMFFHTVDIMDVTESLKGFDVVFLAALVGMDKKEKVKVVEHLEKHMSPGALLMLRSAHG


FL-XI-G1     ARAFLYPVLDPRDLRGFEVLSVY-------------------------------------
NtomNAS      ARAFLYPVLDPRDLRGFEVLSVYHPTDEVINSVVIARKLPLPCVQPLDGLGSYVLPSKCA
```

```
NtNAS1        AR----------------------------------------------------------
NtNAS         ARAFLYPVLDPCDLRGFEVLSVYHPTDEVINSVVIARKLPLPCVQPLDGLGSYVLPSKCA
NsylNAS       ARAFLYPVLDPCDLRGFEVLSVYHPTDEVINSVVIARKLPLPCVQPLDGLGSYVLPSKCA
NtNAS2        AR----------------------------------------------------------
AtNAS1        LRAFLYPIVDSSDLKGFQLLTIYHPTDDVVNSVVIARKLGGPT-TPGVNGTRGCMFMPCN
AtNAS2        LRAFLYPIVDSCDLKGFEVLTIYHPSDDVVNSVVIARKLGGSN-GARGSQIGRCVVMPCN
AtNAS3        PRAFLYPIVEPCDLQGFEVLSIYHPTDDVINSVVISKKHPVVSIGNVGGP-NSCLLKPCN
AtNAS4        PRAFLYPIVEPCDLEGFEVLSVYHPTDEVINSIVISRKLGEDANGVVHDHIDQASDLACN

FL-XI-G1      ------------------------------
NtomNAS       CAEIHAFNPLN--KINLIEEFALEE-----
NtNAS1        ------------------------------
NtNAS         CAEIHAFNPLN--KMNLIEEFALEE-----
NsylNAS       CAEIHAFNPLN--KMNLIEEFALEE-----
NtNAS2        ------------------------------
AtNAS1        CSKIHAIMNNRGK-KNMIEEFSAIE-----
AtNAS2        CSKVHAILNNRGMEKNLIEEYSAIE-----
AtNAS3        CSKTHAKMNKN---MM-IEEF-GAREEQLS
AtNAS4        CSKIHVIMNKK---KSIIEEFAGANEEQLT
```

VTL

X-VIII-G12 559 bp + poliA
GTACTCTCAACTAGACATAGAGCGAGCTCAAATGAAGAGAGACAAAACAACAGGAGGACAGAACCAAAAACATCAACGACAACAAGA
CGAAGGCAACAAGGAGCAGCTGCCAAATCCATTTCAGGCAGCCGTAGCCTCAGCTATTGCATTTTCACTGGGTGCCATTGTGCCAAT
TCTTGCTGCTGCATTTATAGCAGATCATAAGGTGAGGCTAGGTGTGATTGTGGCTGCAGTGAGCTTGGCATTGTTAGCATTTGGAGG
GATTGGTGCTTTTTTGGGCAGAAGTCCTATGCTGAAATCATGTGCCAGAGTTTTAATTGGTGGCTGGATGGCTATGGCCATTACCTT
TGGCCTTACCAAACTGATTGGTTCTACTGGCTTGGAGATTTGATTGGAACTTTATGCTTCCTGTCTCTTGTGCTAGTTTCAAGTTAT
CTGTTTAAGAAATTTCTGCTGCATTGTTGACTAAATTTCCTGACTAAGACTAGAATAAATGCAAGAAATATCTGTGTTATAATTCTG
CTGCGAATGCTTAAAAATGGTATTGCCGCGSTCTCTGAAAAAAAAAAAAAAAAAAAAAAAAA

X-VIII-G12 translated
YSQLDIERAQMKRDKTTGGQNQKHQRQQDEGNKEQLPNPFQAAVASAIAFSLGAIVPILAAAFIADHKVRLGVIVAAVSLALLAFGG
IGAFLGRSPMLKSCARVLIGGWMAMAITFGLTKLIGSTGLEI

ALIGNMENT OF NUCLEOTIDE SEQUENCES

PREDICTED: Nicotiana sylvestris vacuolar iron transporter homolog 4-like (LOC104222620), mRNA
Sequence ID: ref|XM_009773865.1| **Length: 981** Number of Matches: 1
Range 1: 467 to 981 GenBank Graphics Next Match Previous Match

Alignment statistics for match #1

Score	Expect	Identities	Gaps	Strand
939 bits(508)	0.0	**513/515(99%)**	1/515(0%)	Plus/Plus

```
Query  1    GTACTCTCAACTAGACATAGAGCGAGCTCAAATGAAGAGAGACAAAACAACAGGAGGACA  60
            ||||||||||||||||||||||||||||||||||||||||||||||||||||||||||||
Sbjct  467  GTACTCTCAACTAGACATAGAGCGAGCTCAAATGAAGAGAGACAAAACAACAGGAGGACA  526

Query  61   GAACCAAAAACATCAACGACAACAAGACGAAGGCAACAAGGAGCAGCTGCCAAATCCATT  120
            ||||||||||||||||||||||||||||||||||||||||||||||||||||||||||||
Sbjct  527  GAACCAAAAACATCAACGACAACAAGACGAAGGCAACAAGGAGCAGCTGCCAAATCCATT  586

Query  121  TCAGGCAGCCGTAGCCTCAGCTATTGCATTTTCACTGGGTGCCATTGTGCCAATTCTTGC  180
            ||||||||||||||||||||||||||||||||||||||||||||||||||||||||||||
Sbjct  587  TCAGGCAGCCGTAGCCTCAGCTATTGCATTTTCACTGGGTGCCATTGTGCCAATTCTTGC  646

Query  181  TGCTGCATTTATAGCAGATCATAAGGTGAGGCTAGGTGTGATTGTGGCTGCAGTGAGCTT  240
            ||||||||||||||||||||||||||||||||||||||||||||||||||||||||||||
Sbjct  647  TGCTGCATTTATAGCAGATCATAAGGTGAGGCTAGGTGTGATTGTGGCTGCAGTGAGCTT  706

Query  241  GGCATTGTTAGCATTTGGAGGGATTGGTGCTTTTTTGGGCAGAAGTCCTATGCTGAAATC  300
            ||||||||||||||||||||||||||||||||||||||||||||||||||||||||||||
Sbjct  707  GGCATTGTTAGCATTTGGAGGGATTGGTGCTTTTTTGGGCAGAAGTCCTATGCTGAAATC  766

Query  301  ATGTGCCAGAGTTTTAATTGGTGGCTGGATGGCTATGGCCATTACCTTTGGCCTTACCAA  360
            ||||||||||||||||||||||||||||||||||||||||||||||||||||||||||||
Sbjct  767  ATGTGCCAGAGTTTTAATTGGTGGCTGGATGGCTATGGCCATTACCTTTGGCCTTACCAA  826

Query  361  ACTGATTGGTTCTACTGGCTTGGAGATTTGATTGGAACTTTATGCTTCCTGTCTCTTGTG  420
            ||||||||||||||||||||||||||||||||||||||||||||||||||||||||||||
Sbjct  827  ACTGATTGGTTCTACTGGCTTGGAGATTTGATTGGAACTTTATGCTTCCTGTCTCTTGTG  886

Query  421  CTAGTTTCAAGTTATCTG-TTTAAGAAATTTCTGCTGCATTGTTGACTAAATTTCCTGAC  479
            |||||||||||||||||| |||||||||||||||||||||||||||||||||||||||||
Sbjct  887  CTAGTTTCAAGTTATCTGTTTTAAGAAATTTCTGCTGCATTGTTGACTAAATTTCCTGAC  946

Query  480  TAAGACTAGAATAAATGCAAGAAATATCTGTGTTA  514
            |||||||||||||||||||||||||||||| ||||
Sbjct  947  TAAGACTAGAATAAATGCAAGAAATATCTGCGTTA  981
```

PREDICTED: Nicotiana tomentosiformis vacuolar iron transporter homolog 4-like (LOC104084877), mRNA

Sequence ID: ref|XM_009588835.1| **Length: 820** Number of Matches: 1

Range 1: 273 to 820GenBankGraphics Next Match Previous Match

Alignment statistics for match #1

Score	Expect	Identities	Gaps	Strand
863 bits(467)	0.0	**521/548(95%)**	0/548(0%)	Plus/Plus

```
Query  1    GTACTCTCAACTAGACATAGAGCGAGCTCAAATGAAGAGAGACAAAACAACAGGAGGACA  60
            ||| ||||||||||||||||| | ||||||||||||||| ||||||||||||| ||| ||
Sbjct  273  GTATTCTCAACTAGACATAGAACTAGCTCAAATGAAGAGGGACAAAACAACAGAAGGGCA  332

Query  61   GAACCAAAAACATCAACGACAACAAGACGAAGGCAACAAGGAGCAGCTGCCAAATCCATT  120
            ||||||| ||||||||| ||||||||| ||||||||||||||||||||||||||||||||
Sbjct  333  GAACCAAGAACATCAACAACAACAAGAAGAAGGCAACAAGGAGCAGCTGCCAAATCCATT  392


Query  121  TCAGGCAGCCGTAGCCTCAGCTATTGCATTTTCACTGGGTGCCATTGTGCCAATTCTTGC  180
            |||||||||||||||||||||||||||||||||||| |||||||||||||||||||||||
Sbjct  393  TCAGGCAGCCGTAGCCTCAGCTATTGCATTTTCACTAGGTGCCATTGTGCCAATTCTTGC  452

Query  181  TGCTGCATTTATAGCAGATCATAAGGTGAGGCTAGGTGTGATTGTGGCTGCAGTGAGCTT  240
            |||||||||||||||| |||||||||||||||||| ||||||||||||||||||||| ||
Sbjct  453  TGCTGCATTTATAGCAAATCATAAGGTGAGGCTAGCTGTGATTGTGGCTGCAGTGAGTTT  512

Query  241  GGCATTGTTAGCATTTGGAGGGATTGGTGCTTTTTTGGGCAGAAGTCCTATGCTGAAATC  300
            |||||||||||||||||||||||||||||||||||||||||||||||| |||||||||||
Sbjct  513  GGCATTGTTAGCATTTGGAGGGATTGGTGCTTTTTTGGGCAGAAGTCCAATGCTGAAATC  572

Query  301  ATGTGCCAGAGTTTTAATTGGTGGCTGGATGGCTATGGCCATTACCTTTGGCCTTACCAA  360
            ||||||||||||||||||||||||||||||||| ||||||||||||||||||||||||||
Sbjct  573  ATGTGCCAGAGTTTTAATTGGTGGCTGGATGGCAATGGCCATTACCTTTGGCCTTACCAA  632

Query  361  ACTGATTGGTTCTACTGGCTTGGAGATTTGATTGGAACTTTATGCTTCCTGTCTCTTGTG  420
            ||||||||||||||||||| ||||||  ||||| |||||||||| |||||||  ||||| 
Sbjct  633  ACTGATTGGTTCTACTGGCATGGAGACGTGATTCGAACTTTATGGTTCCTGTTCCTTGTT  692

Query  421  CTAGTTTCAAGTTATCTGTTTAAGAAATTTCTGCTGCATTGTTGACTAAATTTCCTGACT  480
            |||||||||| |||||||||||||||||||||||||||| |||||||||||||||||||||
Sbjct  693  CTAGTTTCAAATTATCTGTTTAAGAAATTTCTGCTGCACTGTTGACTAAATTTCCTGACT  752

Query  481  AAGACTAGAATAAATGCAAGAAATATCTGTGTTATAATTCTGCTGCGAATGCTTAAAAAT  540
            ||||||||||||||||||||| |||||||||||||||||||||||||||||||||| |||
Sbjct  753  AAGACTAGAATAAATGCAAGACATATCTGTGTTATAATTCTGCTGCGAATGCTTAAGAAT  812

Query  541  GGTATTGC  548
            ||||||||
Sbjct  813  GGTATTGC  820
```

FS403146 normalized full-length tobacco cDNA library Nicotiana tabacum cDNA clone TBK02GR0007_2_B07 5', mRNA sequence.

Sequence ID: dbj|FS403146.1| **Length: 671** Number of Matches: 1

Range 1: 337 to 671GenBankGraphics Next Match Previous Match

Alignment statistics for match #1

Score	Expect	Identities	Gaps	Strand
444 bits(240)	2e-123	**306/337(91%)**	7/337(2%)	Plus/Plus

```
Query  1    GTACTCTCAACTAGACATAGAGCGAGCTCAAATGAAGAGAGACAAAACAACAGGAGGACA  60
            ||||||||||||||||||||||  ||||||||||||||||||||||||||||||||||||
Sbjct  337  GTACTCTCAACTAGACATAGAGTTAGCTCAAATGAAGAGAGACAAAACAACAGGAGGACA  396

Query  61   GAACCAAAAACATCAACGACAACAAGACGAAG---GCAACAAGGAGCAGCTGCCAAATCC  117
            |||| || |||||||||  ||| |||  ||||   | |||||||| ||||||||||||||
Sbjct  397  GAACAAAGAACATCAACAGCAAGAAGTAGAAGAAAGTAACAAGGAACAGCTGCCAAATCC  456

Query  118  ATTTCAGGCAGCCGTAGCCTC-AGCTATTGCATTTTCACTGGGTGCCATTGTGCCAATTC  176
            ||||||||||||||  ||||| || ||||||||||||| ||||||| |||||||||||||
Sbjct  457  ATTTCAGGCAGCCGGTGCCTCGAG-TATTGCATTTTCATTGGGTGCTATTGTGCCAATTC  515

Query  177  TTGCTGCTGCATTTATAGCAGATCATAAGGTGAGGCTAGGTGTGATTGTGGCTGCAGTGA  236
```

```
                |||| |||||||||||||||||||||||||||||||||| ||||||||||||||||||||
Sbjct  516  TTGCAGCTGCATTTATAGCAGATCATAAGGTGAGGCTAGCTGTGATTGTGGCTGCAGTGA  575

Query  237  GCTTGGCATTGTTAGCATTTGGAGGGATTGGTGCTTTTTTGGGCAGAAGTCCTATGCTGA  296
            |||| ||||||||||||||||||||  ||||||||||| ||||||||||||||||| | |
Sbjct  576  GCTTAGCATTGTTAGCATTTGGAGGAGTTGGTGCTTTTCTGGGCAGAAGTCCTATGGTCA  635

Query  297  AATCATGTGCCAGAGTTTTAATTGGTGGCT-GGATGG  332
            |||| ||||||||||||| ||||||||||| ||||||
Sbjct  636  AATCTTGTGCCAGAGTTT-AATTGGTGGCTTGGATGG  671
```

PREDICTED: Nicotiana sylvestris vacuolar iron transporter homolog 4-like (LOC104222621), mRNA

Sequence ID: ref|XM_009773866.1| **Length: 1099** Number of Matches: 1

Range 1: 453 to 932GenBankGraphics Next Match Previous Match

Alignment statistics for match #1

Score	Expect	Identities	Gaps	Strand
518 bits(280)	5e-145	**419/485(86%)**	14/485(2%)	Plus/Plus

```
Query  1    GTACTCTCAACTAGACATAGAGCGAGCTCAAATGAAGAGAGACAAAACAACAGGAGGACA  60
            ||||||||||||||||||||||  ||||||||||||||||||||||||||||||||||||
Sbjct  453  GTACTCTCAACTAGACATAGAGTTAGCTCAAATGAAGAGAGACAAAACAACAGGAGGACA  512

Query  61   GAACCAAAAACATCAACGACAA-CA--AGACGAAGGCAACAAGGAGCAGCTGCCAAATCC  117
            |||| || |||||||||  |||  |  ||| ||| | |||||||| ||||||||||||||
Sbjct  513  GAACAAAGAACATCAACAGCAAGAAGTAGAAGAAAGTAACAAGGAACAGCTGCCAAATCC  572

Query  118  ATTTCAGGCAGCCGTAGCCTC-AGCTATTGCATTTTCACTGGGTGCCATTGTGCCAATTC  176
            ||||||||||||||  ||||| || ||||||||||||| ||||||| |||||||||||||
Sbjct  573  ATTTCAGGCAGCCGGTGCCTCGAG-TATTGCATTTTCATTGGGTGCTATTGTGCCAATTC  631

Query  177  TTGCTGCTGCATTTATAGCAGATCATAAGGTGAGGCTAGGTGTGATTGTGGCTGCAGTGA  236
            |||| |||||||||||||||||||||||||||||||||| ||||||||||||||||||||
Sbjct  632  TTGCAGCTGCATTTATAGCAGATCATAAGGTGAGGCTAGCTGTGATTGTGGCTGCAGTGA  691

Query  237  GCTTGGCATTGTTAGCATTTGGAGGGATTGGTGCTTTTTTGGGCAGAAGTCCTATGCTGA  296
            |||| ||||||||||||||||||||  ||||||||||| ||||||||||||||||| | |
Sbjct  692  GCTTAGCATTGTTAGCATTTGGAGGAGTTGGTGCTTTTCTGGGCAGAAGTCCTATGGTCA  751

Query  297  AATCATGTGCCAGAGTTTTAATTGGTGGCTGGATGGCTATGGCCATTACCTTTGGCCTTA  356
            |||| |||||||||||||||||||||||||||||||||||||||||||| |||||||| |
Sbjct  752  AATCTTGTGCCAGAGTTTTAATTGGTGGCTGGATGGCTATGGCCATTACGTTTGGCCTCA  811

Query  357  CCAAACTGATTGGTTCTACTGGCTTGGAGATTTGATTGGAACTTTATGCTTCCTGT-CTC  415
            | | | ||||||| ||||  || ||||| || |||| || ||| |||| ||| ||| | |
Sbjct  812  CTAGATTGATTGGCTCTATGGGATTGGAAATGTGATGGGGACTCTATG-TTC-TGTTCCC  869

Query  416  T-TG-T-GCTAGTTTCAAG-TTATCTGTTTAAGAAATTTCTGCTGCATTGTTGACTAAAT  471
            | |  | |||| ||| |   ||||||| | ||||||||  || ||||  ||  | |||||
Sbjct  870  TGTCCTAGCTAATTT-AGTTTTATCTGCTCAAGAAATTGATGTTGCAC-GTCAAATAAAT  927

Query  472  TTCCT  476
            |||||
Sbjct  928  TTCCT  932
```

Arabidopsis thaliana vacuolar iron transporter homolog 2.1 mRNA, complete cds

Sequence ID: ref|NM_113425.1| **Length: 870** Number of Matches: 1

Related Information

Range 1: 388 to 690GenBankGraphics Next Match Previous Match

Alignment statistics for match #1

Score	Expect	Identities	Gaps	Strand
111 bits(122)	7e-23	**207/303(68%)**	1/303(0%)	Plus/Plus

```
Query  72   ATCAACGA-CAACAAGACGAAGGCAACAAGGAGCAGCTGCCAAATCCATTTCAGGCAGCC  130
            ||| |||| ||| | || || |  || || ||   |||||||||||||   || |||||
Sbjct  388  ATCGACGAACAAGAGGAGGAGGAGAAGAAAGAAAGGCTGCCAAATCCAGGGCAAGCAGCA  447

Query  131  GTAGCCTCAGCTATTGCATTTTCACTGGGTGCCATTGTGCCAATTCTTGCTGCTGCATTT  190
             | || || ||  | || |||||| |||||||     ||||  |||| |  |||| |||
Sbjct  448  ATTGCATCGGCGTTAGCGTTTTCAGTGGGTGCAGCAATGCCGCTTCTGGGCGCTGTATTC  507
```

```
Query  191  ATAGCAGATCATAAGGTGAGGCTAGGTGTGATTGTGGCTGCAGTGAGCTTGGCATTGTTA  250
            ||||   |||||||||| ||  | |  ||| | | || ||  |  | | | |||||| |
Sbjct  508  ATAGAGAATCATAAGGTAAGAATGGTGGTGGTAGCGGTTGTGGCCACCATAGCATTGGTT  567

Query  251  GCATTTGGAGGGATTGGTGCTTTTTTGGGCAGAAGTCCTATGCTGAAATCATGTGCCAGA  310
            |  ||||||| || ||||||  | ||||| |  |    | || | || ||  | | |||
Sbjct  568  GTGTTTGGAGTGACTGGTGCGGTCTTGGGAAAGACAAGTGTGGTTAAGTCGAGCGTCAGG  627

Query  311  GTTTTAATTGGTGGCTGGATGGCTATGGCCATTACCTTTGGCCTTACCAAACTGATTGGT  370
            ||  | |||||||| ||||||||||||||  |||||||||| || |||||  | |||||
Sbjct  628  GTGGTGATTGGTGGTTGGATGGCTATGGCTCTTACCTTTGGTCTCACCAAGTTCATTGGC  687

Query  371  TCT  373
            |||
Sbjct  688  TCT  690
```

ALIGNMENT OF AMINO ACID SEQUENCES

Alignment of predicted amino acid sequence X-VIII-G12 from *N. tabacum* v. Xanthi (nucleotide sequence identified by SSH) with selected proteins from *Nicotiana* species and from *A. thaliana*.

PREDICTED: vacuolar iron transporter homolog 4-like [Nicotiana sylvestris]
Sequence ID: ref|XP_009772167.1| **Length: 218** Number of Matches: 1
Range 1: 90 to 218GenPeptGraphics Next Match Previous Match

Alignment statistics for match #1

Score	Expect	Method	Identities	Positives	Gaps
261 bits(666)	2e-88	Compositional matrix adjust.	**129/129(100%)**	**129/129(100%)**	0/129(0%)

```
Query  1    YSQLDIERAQMKRDKTTGGQNQKHQRQQDEGNKEQLPNPFQAAVASAIAFSLGAIVPILA  60
            YSQLDIERAQMKRDKTTGGQNQKHQRQQDEGNKEQLPNPFQAAVASAIAFSLGAIVPILA
Sbjct  90   YSQLDIERAQMKRDKTTGGQNQKHQRQQDEGNKEQLPNPFQAAVASAIAFSLGAIVPILA  149

Query  61   AAFIADHKVRLGVIVAAVSLALLAFGGIGAFLGRSPMLKSCARVLIGGWMAMAITFGLTK  120
            AAFIADHKVRLGVIVAAVSLALLAFGGIGAFLGRSPMLKSCARVLIGGWMAMAITFGLTK
Sbjct  150  AAFIADHKVRLGVIVAAVSLALLAFGGIGAFLGRSPMLKSCARVLIGGWMAMAITFGLTK  209

Query  121  LIGSTGLEI  129
            LIGSTGLEI
Sbjct  210  LIGSTGLEI  218
```

PREDICTED: vacuolar iron transporter homolog 4-like [Nicotiana sylvestris]
Sequence ID: ref|XP_009772168.1| **Length: 219** Number of Matches: 1
Range 1: 90 to 219GenPeptGraphics Next Match Previous Match

Alignment statistics for match #1

Score	Expect	Method	Identities	Positives	Gaps
231 bits(589)	9e-77	Compositional matrix adjust.	**114/130(88%)**	**124/130(95%)**	1/130(0%)

```
Query  1    YSQLDIERAQMKRDKTTGGQNQKHQRQQ-DEGNKEQLPNPFQAAVASAIAFSLGAIVPIL  59
            YSQLDIE AQMKRDKTTGGQN++HQ+Q+ +E NKEQLPNPFQAA AS+IAFSLGAIVPIL
Sbjct  90   YSQLDIELAQMKRDKTTGGQNKEHQQQEVEESNKEQLPNPFQAAGASSIAFSLGAIVPIL  149

Query  60   AAAFIADHKVRLGVIVAAVSLALLAFGGIGAFLGRSPMLKSCARVLIGGWMAMAITFGLT  119
            AAAFIADHKVRL VIVAAVSLALLAFGG+GAFLGRSPM+KSCARVLIGGWMAMAITFGLT
Sbjct  150  AAAFIADHKVRLAVIVAAVSLALLAFGGVGAFLGRSPMVKSCARVLIGGWMAMAITFGLT  209

Query  120  KLIGSTGLEI  129
            +LIGS GLE+
Sbjct  210  RLIGSMGLEM  219
```

PREDICTED: vacuolar iron transporter homolog 4-like [Nicotiana tomentosiformis]
Sequence ID: reflXP_009587130.1l **Length: 220** Number of Matches: 1
Range 1: 92 to 219GenPeptGraphics Next Match Previous Match

Alignment statistics for match #1

Score	Expect	Method	Identities	Positives	Gaps
211 bits(538)	4e-69	Compositional matrix adjust.	**120/128(94%)**	**125/128(97%)**	0/128(0%)

```
Query  1    YSQLDIERAQMKRDKTTGGQNQKHQRQQDEGNKEQLPNPFQAAVASAIAFSLGAIVPILA  60
            YSQLDIE AQMKRDKTT GQNQ+HQ+QQ+EGNKEQLPNPFQAAVASAIAFSLGAIVPILA
Sbjct  92   YSQLDIELAQMKRDKTTEGQNQEHQQQQEEGNKEQLPNPFQAAVASAIAFSLGAIVPILA  151

Query  61   AAFIADHKVRLGVIVAAVSLALLAFGGIGAFLGRSPMLKSCARVLIGGWMAMAITFGLTK  120
            AAFIA+HKVRL VIVAAVSLALLAFGGIGAFLGRSPMLKSCARVLIGGWMAMAITFGLTK
Sbjct  152  AAFIANHKVRLAVIVAAVSLALLAFGGIGAFLGRSPMLKSCARVLIGGWMAMAITFGLTK  211

Query  121  LIGSTGLE  128
            LIGSTG+E
Sbjct  212  LIGSTGME  219
```

PREDICTED: vacuolar iron transporter homolog 4-like [Nicotiana sylvestris]
Sequence ID: reflXP_009772166.1l **Length: 218** Number of Matches: 1
Range 1: 92 to 218GenPeptGraphics Next Match Previous Match

Alignment statistics for match #1

Score	Expect	Method	Identities	Positives	Gaps
172 bits(435)	1e-53	Compositional matrix adjust.	**92/129(71%)**	**109/129(84%)**	2/129(1%)

```
Query  1    YSQLDIERAQMKRDKTTGGQNQKHQRQQDEGNKEQLPNPFQAAVASAIAFSLGAIVPILA  60
            YSQLDIE AQ+KR+ T  GQN  H+ Q +E  KE LPNPF AA+ASA+AFSLG I+PILA
Sbjct  92   YSQLDIELAQIKRENTIRGQN--HEEQGEEDKKEGLPNPFLAAIASAVAFSLGGIIPILA  149

Query  61   AAFIADHKVRLGVIVAAVSLALLAFGGIGAFLGRSPMLKSCARVLIGGWMAMAITFGLTK  120
            A FI++HKVR+ V+VAAVSLAL  FGG+GA LGR+P ++SCARVLIGGWMAMAITFGLTK
Sbjct  150  AGFISNHKVRMAVVVAAVSLALFVFGGVGAVLGRTPAVRSCARVLIGGWMAMAITFGLTK  209

Query  121  LIGSTGLEI  129
            LIGS G+E+
Sbjct  210  LIGSAGMEM  218
```

PREDICTED: vacuolar iron transporter homolog 1-like [Nicotiana tomentosiformis]
Sequence ID: reflXP_009618045.1l**Length: 317** Number of Matches: 1
Range 1: 195 to 317GenPeptGraphics Next Match Previous Match

Alignment statistics for match #1

Score	Expect	Method	Identities	Positives	Gaps
156 bits(394)	2e-46	Compositional matrix adjust.	**83/127(65%)**	**106/127(83%)**	4/127(3%)

```
Query  1    YSQLDIERAQMKRDKTTGGQNQKHQRQQDEGNKEQLPNPFQAAVASAIAFSLGAIVPILA  60
            YSQLDIE AQMKR+K       +K    +D+  K+ LPNP QAA ASA+AFS+GA+VP+LA
Sbjct  195  YSQLDIEVAQMKREKRRNANREK----EDDDAKDSLPNPAQAAAASALAFSVGAMVPLLA  250

Query  61   AAFIADHKVRLGVIVAAVSLALLAFGGIGAFLGRSPMLKSCARVLIGGWMAMAITFGLTK  120
            A+FI D+KVR+GV+VAAV+LAL+ FG +GA LG++P++KS ARVL+GGW+AMAITFGLTK
Sbjct  251  ASFIRDYKVRIGVVVAAVTLALMVFGWLGAALGKAPVVKSSARVLLGGWLAMAITFGLTK  310

Query  121  LIGSTGL  127
```

```
              LIGS+GL
Sbjct  311  LIGSSGL  317
```

PREDICTED: vacuolar iron transporter homolog 1-like [Nicotiana sylvestris]
Sequence ID: ref|XP_009780590.1| **Length: 218** Number of Matches: 1
Range 1: 97 to 216GenPeptGraphics Next Match Previous Match

Alignment statistics for match #1

Score	Expect	Method	Identities	Positives	Gaps
142 bits(358)	4e-42	Compositional matrix adjust.	**76/127(60%)**	**97/127(76%)**	7/127(5%)

```
Query  1    YSQLDIERAQMKRDKTTGGQNQKHQRQQDEGNKEQLPNPFQAAVASAIAFSLGAIVPILA  60
            YSQ DIE +QMKR      QN     + +E  K+ LP+P QAA ASA AF++GAIVP+LA
Sbjct  97   YSQYDIEMSQMKR------QNNSSTHELEE-KKKNLPSPLQAAAASAFAFAIGAIVPLLA  149

Query  61   AAFIADHKVRLGVIVAAVSLALLAFGGIGAFLGRSPMLKSCARVLIGGWMAMAITFGLTK  120
            AAF+ ++ +RLG++V AVS ALL FGG+GA+LG +P+LKS  RVLIGGW++M ITFGLTK
Sbjct  150  AAFVKNYHIRLGIVVTAVSFALLVFGGLGAYLGNAPLLKSSLRVLIGGWLSMGITFGLTK  209

Query  121  LIGSTGL  127
            L+G TGL
Sbjct  210  LVGVTGL  216
```

putative nodulin-like21 protein [Arabidopsis thaliana] (AT3G43660)
Sequence ID: ref|NP_189952.1| **Length: 198** Number of Matches: 1
Range 1: 84 to 198GenPeptGraphics Next Match Previous Match

Alignment statistics for match #1

Score	Expect	Method	Identities	Positives	Gaps
157 bits(397)	3e-48	Compositional matrix adjust.	**80/127(63%)**	**103/127(81%)**	12/127(9%)

```
Query  1    YSQLDIERAQMKRDKTTGGQNQKHQRQQDEGNKEQLPNPFQAAVASAIAFSLGAIVPILA  60
            YSQ DIE AQMKR+   +GG+ +K          E+LP+P QAA+ASA+AF+LGAIVP+LA
Sbjct  84   YSQYDIEVAQMKRE--SGGETKK----------EKLPSPTQAAIASALAFTLGAIVPLLA  131

Query  61   AAFIADHKVRLGVIVAAVSLALLAFGGIGAFLGRSPMLKSCARVLIGGWMAMAITFGLTK  120
            AAF+ ++KVR+GVIVAAV+LAL+ FG +GA LG++P++KS  RVLIGGW+AMAITFG TK
Sbjct  132  AAFVKEYKVRIGVIVAAVTLALVMFGWLGAVLGKAPVVKSLVRVLIGGWLAMAITFGFTK  191

Query  121  LIGSTGL  127
            L+GS GL
Sbjct  192  LVGSHGL  198
```

vacuolar iron transporter homolog 1 [Arabidopsis thaliana] (AT1G21140)
Sequence ID: ref|NP_173538.2| **Length: 200** Number of Matches: 1
Range 1: 86 to 200GenPeptGraphics Next Match Previous Match

Alignment statistics for match #1

Score	Expect	Method	Identities	Positives	Gaps
136 bits(343)	4e-40	Compositional matrix adjust.	**80/127(63%)**	**99/127(77%)**	12/127(9%)

```
Query  1    YSQLDIERAQMKRDKTTGGQNQKHQRQQDEGNKEQLPNPFQAAVASAIAFSLGAIVPILA  60
            YSQ DIE AQMKR+   GGQ +K          E+LP+P QAA ASA+AFSLGAIVP++A
Sbjct  86   YSQYDIEVAQMKRE--NGGQVEK----------EKLPSPMQAAAASALAFSLGAIVPLMA  133

Query  61   AAFIADHKVRLGVIVAAVSLALLAFGGIGAFLGRSPMLKSCARVLIGGWMAMAITFGLTK  120
            AAF+ D+ VR+G IVAAV+LAL+ FG +GA LG++P+ KS ARVLIGGW+AMA+TFGLTK
Sbjct  134  AAFVKDYHVRIGAIVAAVTLALVMFGWLGAVLGKAPVFKSSARVLIGGWLAMAVTFGLTK  193

Query  121  LIGSTGL  127
            LIG+  L
Sbjct  194  LIGTHSL  200
```
